# Searching for Effective Treatments in HFpEF: Implications for Modeling the Disease in Rodents

**DOI:** 10.3390/ph16101449

**Published:** 2023-10-12

**Authors:** Magdalena Jasińska-Stroschein

**Affiliations:** Department of Biopharmacy, Medical University of Lodz, 90-419 Lodz, Poland; magdalena.jasinska-stroschein@umed.lodz.pl

**Keywords:** preserved left ventricular function, heart failure, treatment, meta-research, preclinical model, rodents

## Abstract

Background: While the prevalence of heart failure with preserved ejection fraction (HFpEF) has increased over the last two decades, there still remains a lack of effective treatment. A key therapeutic challenge is posed by the absence of animal models that accurately replicate the complexities of HFpEF. The present review summarizes the effects of a wide spectrum of therapeutic agents on HF. Methods: Two online databases were searched for studies; in total, 194 experimental protocols were analyzed following the PRISMA protocol. Results: A diverse range of models has been proposed for studying therapeutic interventions for HFpEF, with most being based on pressure overload and systemic hypertension. They have been used to evaluate more than 150 different substances including ARNIs, ARBs, HMGR inhibitors, SGLT-2 inhibitors and incretins. Existing preclinical studies have primarily focused on LV diastolic performance, and this has been significantly improved by a wide spectrum of candidate therapeutic agents. Few experiments have investigated the normalization of pulmonary congestion, exercise capacity, animal mortality, or certain molecular hallmarks of heart disease. Conclusions: The development of comprehensive preclinical HFpEF models, with multi-organ system phenotyping and physiologic stress-based functional testing, is needed for more successful translation of preclinical research to clinical trials.

## 1. Introduction

Heart failure with preserved ejection fraction (HFpEF) has recently been recognized as a complex clinical syndrome characterized by alterations in various organ systems. Currently, HFpEF affects nearly five percent of the general population aged ≥60 years and accounts for approximately half of the total HF hospitalizations [1]. Moreover, the prognosis for HFpEF remains poor, with mortality rates comparable to HF with reduced ejection fraction (HFrEF) [2]. To date, no treatment has been shown to reduce clinical events including cardiovascular (CV) and all-cause mortality in HFpEF [3].

HFpEF presents a major challenge for translational and therapeutic preclinical and clinical research. It is characterized by a “preserved” (i.e., 50%) left ventricular (LV) ejection fraction (LVEF), abnormal LV filling, and elevated filling pressures. The pathophysiology of the HFpEF disease is multifactorial. Despite “normal” ejection fraction, HFpEF is characterized by diastolic function, characterized by impaired LV relaxation and/or increase in LV stiffness, cardiac reserves, systemic and pulmonary vascular function, renal function, oxygen carrying capacity, and peripheral oxygen extraction [4] Such heterogeneity has not carried over to preclinical investigation, where currently available animal models have primarily focused on diastolic function [5,6]. In a recent paper, the efficacy of models created to induce heart failure by the most common comorbidities associated with human HFpEF (e.g., systemic hypertension and pressure overload, diabetic cardiomyopathy, as well as aging) has been described and compared quantitatively according to data from more than 500 experimental protocols [7]. In addition to the heterogeneous pathophysiological background, other disadvantages of using murine models to study the HFpEF phenotype include a relatively short-term induction of heart failure and a greater chance of progression from HFpEF to HFrEF over longer experimental periods compared to human disease.

Treatments for HFpEF have been qualitatively summarized and discussed in a previous review [8] emphasizing the need for a particular model to yield reproducible and repeatable data, which can be extended to reflect human disease. However, little pooled quantitative evidence was available to compare the effect of such interventions.

The present review summarizes the effects of a wide spectrum of therapeutic agents on heart failure. LVEF remains a key consideration when identifying appropriate animal models of HFpEF, and special attention has been paid to its potential influence on the efficacy of candidate therapeutic interventions. More specifically, the following questions were addressed: (a) What treatment goals for HF were achieved in relation to individual potential candidates to treat the disease? (b) What animal models were chosen to evaluate these effects? (c) Could these experimental approaches mimic human HFpEF? (d) Could they provide the reproducible results given by individual medicine agents? (e) What were the factors that could influence the therapeutic efficacy observed in a particular animal model? These may well play an important role in identifying critical obstacles to therapeutic innovation in HFpEF based on preclinical investigation.

## 2. Results

### 2.1. Selection of Studies

**Figure 1:**
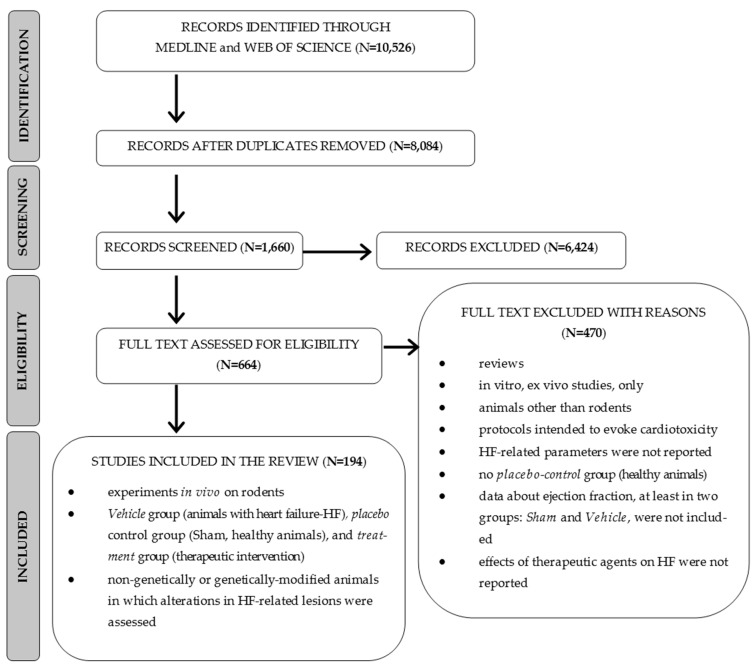
PRISMA flowchart of the study selection process.

In total, 10,526 articles were searched, and 664 papers were found to be potentially relevant to the review question. Of these, all protocols reporting the ejection fraction values for at least the Vehicle and Sham groups were included in the final analysis (N = 194 studies and 239 interventions), as presented in Figure 1 [9,10,11,12,13,14,15,16,17,18,19,20,21,22,23,24,25,26,27,28,29,30,31,32,33,34,35,36,37,38,39,40,41,42,43,44,45,46,47,48,49,50,51,52,53,54,55,56,57,58,59,60,61,62,63,64,65,66,67,68,69,70,71,72,73,74,75,76,77,78,79,80,81,82,83,84,85,86,87,88,89,90,91,92,93,94,95,96,97,98,99,100,101,102,103,104,105,106,107,108,109,110,111,112,113,114,115,116,117,118,119,120,121,122,123,124,125,126,127,128,129,130,131,132,133,134,135,136,137,138,139,140,141,142,143,144,145,146,147,148,149,150,151,152,153,154,155,156,157,158,159,160,161,162,163,164,165,166,167,168,169,170,171,172,173,174,175,176,177,178,179,180,181,182,183,184,185,186,187,188,189,190,191,192,193,194,195,196,197,198,199,200,201,202]. The total number of animals was 18,046. More detailed information about the reviewed studies is provided in the Supplementary Material S1 (Table S1).

### 2.2. Quality Assessments

**Figure 2:**
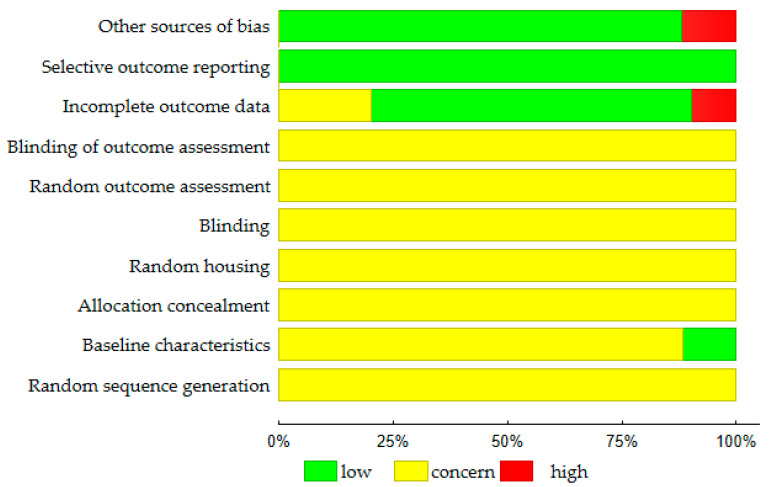
Results of the risk of bias assessment (N = 194 studies) according to the SYRCLE Risk of Bias strategy [203].

The evaluation of risk of bias is summarized in Figure 2. In 62.6 percent of the experimental protocols, the animals were randomly allocated to the respective groups; however, no methods were specified (unclear risk of bias). In the remaining studies, it was not reported whether the randomization process was carried out (unclear risk of bias). In 59 out of 194 papers (30.4%), it was stated that the outcome assessment was blinded. This concerned mainly histomorphometric and statistical analyses. However, the studies did not provide any detailed information about the procedure used to blind researchers from knowing which procedure (e.g., induction of heart failure, therapeutic intervention) was attributed to each animal (unclear risk of bias). Twenty seven of the 194 papers (13.9%) gave the baseline characteristics of the animal subjects (low risk of bias). Also, 137 of the 194 papers (70.6%) reported the number of animals per each experimental group (before and at the end of the study) (low risk of bias). However, 19 articles gave no information on the number of subjects that began and/or that finished the study (high risk of bias). Another potential source of bias (23 trials) was that the different parameters were evaluated in the control (Sham, Vehicle) and therapeutic group (high risk of bias).

The results of the publication bias analysis are presented in Table S2. In the analysis, the animals were allocated to several subgroups according to the therapeutic agent and parameter associated with HF. A non-significant Egger (*p* > 0.05) test result and low possibility of missing studies (trim and fill procedure) seem to indicate the absence of publication bias across the majority of studies.

### 2.3. Animal Models and Therapeutic Agents

**Figure 3:**
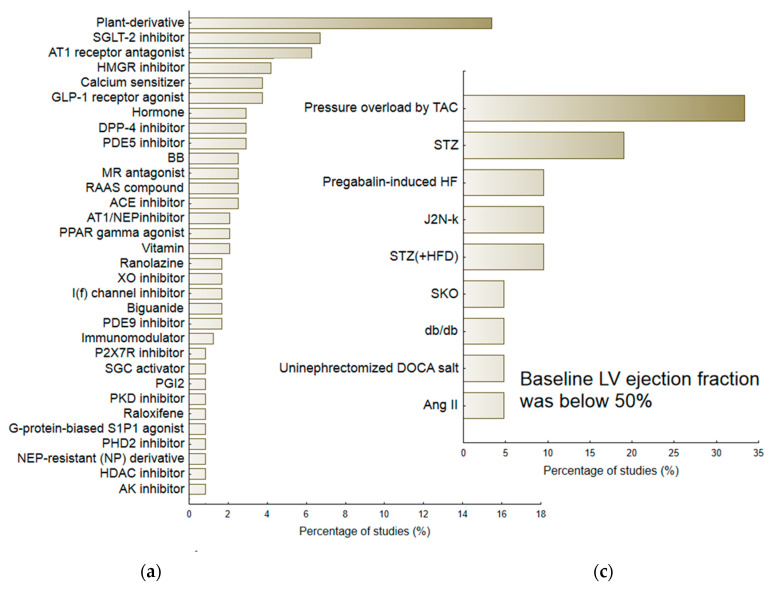

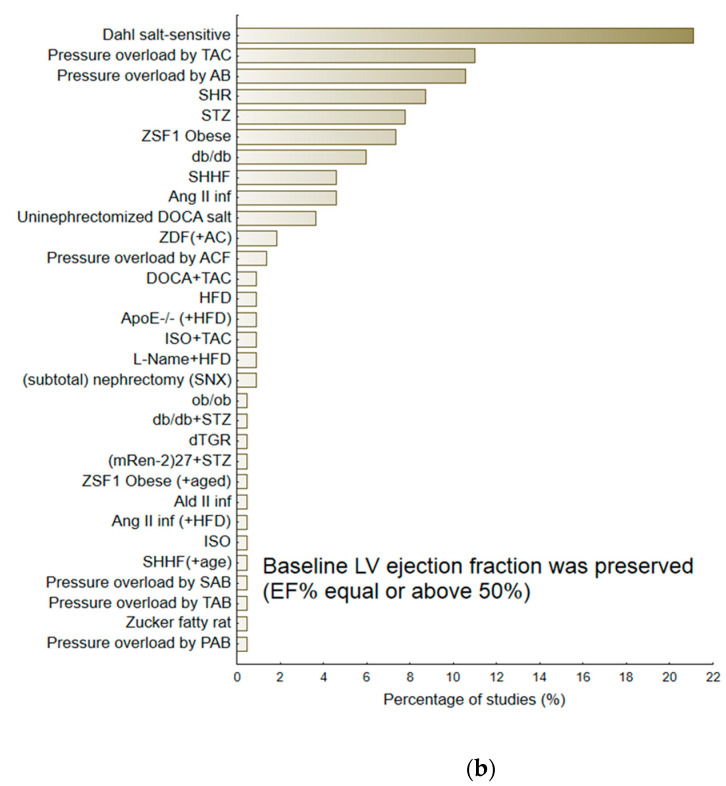
The percentage of reviewed experimental protocols used to evaluate the therapeutic efficacy of pharmacological agents according to the therapeutic group/pharmacological class (**a**) and according to the particular animal model of heart failure (**b**,**c**). The minimal percentage of interventions was 0.5.

The review included a total of 194 studies and 239 interventions. The term intervention indicated a separate comparison between the Vehicle group (subjects with HF receiving placebo) and healthy animals, or between the Treatment group (subjects with HF receiving therapeutic agent) and Vehicle [7]. The majority of interventions used male animals (N = 174/239; 72.8%). These included 152 different substances that were classified into 71 pharmacological/therapeutic groups (Figure 3a). For the purposes of further analyses, the most numerous (found in above 1% of studies) and homogenous groups of medicine agents were considered, viz. Angiotensin Converting Enzyme (ACE) inhibitors (ACEI), Angiotensin II Receptor Type 1 (AT-1) antagonists (ARB), beta-blockers (BB), biguanides, calcium sensitizers, Dipeptidyl Peptidase-4 (DPP-4) inhibitors, Glucagon-Like Peptide 1 (GLP-1) receptor agonists, HMG-CoA Reductase (HMGR) inhibitors, I(f) current inhibitors, MR—Mineralocorticoid-Receptor (MR) antagonists, Neprilysin (NEP) inhibitor/AT1 receptor antagonists (ARNI), Phosphodiesterase (PDE) type 3, 5, 9 inhibitors, Peroxisome Proliferator-Activated Receptor (PPAR) gamma agonists, Sodium-glucose co-transporter-2 (SGLT-2) inhibitors and Xanthine Oxidase (XO) inhibitors. Data about the less homogenous but most numerous group of plant-derivatives were also included in calculations.

Of the 239 interventions, 218 (81.3%) examining various animal models of heart failure gave baseline values of ejection fraction equal to or above 50%. In general, the baseline LVEF in this subgroup of animals was 67.69 (95%CI 66.08; 69.29). The second subgroup included animals with a mean LVEF below 50%, i.e., 41.58 (95%CI 39.16; 44.00). Figure 3b,c demonstrate the percentage of particular HF models. The largest group of HF-models used for evaluating candidate therapeutic agents, i.e., with preserved LVEF (≥50%), included those that were based on pressure-induced overload by transverse aortic constriction (TAC) or aortic banding (AB) (N = 47/218), Dahl-salt sensitive subjects (N = 46 interventions; 21.1%), SHR subjects (N = 19; 8.7%), animals exposed to streptozotocin (STZ) (N = 17, 7.8%), or ZFF1 Obese rats (N = 16; 7.3%). In contrast, the HF-models with reduced LVEF (<50%) included ones based on pressure induced overload by transverse aortic constriction (TAC) (N = 7; 33.3%) or STZ-induced HF (N = 4; 19.0%). More detailed information regarding particular animal models of HF is provided in the Supplementary Material. Table S3 demonstrates their potential to deteriorate the majority of heart failure-linked parameters, particularly diastolic dysfunction: Active diastolic relaxation (d*P*/d*t*_min_, tau, and E/A), passive stiffness (LVEDP, end-diastolic stiffness), left ventricular end-diastolic dimension (LVEDd), pulmonary edema/lung congestion, as well as LV structure (concentric hypertrophy, LVH), and fibrosis.

**Figure 4:**
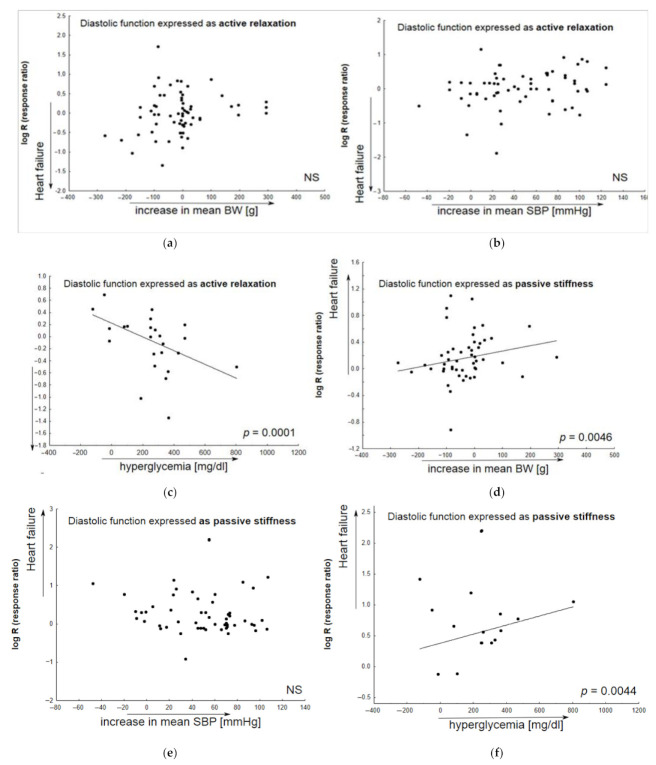

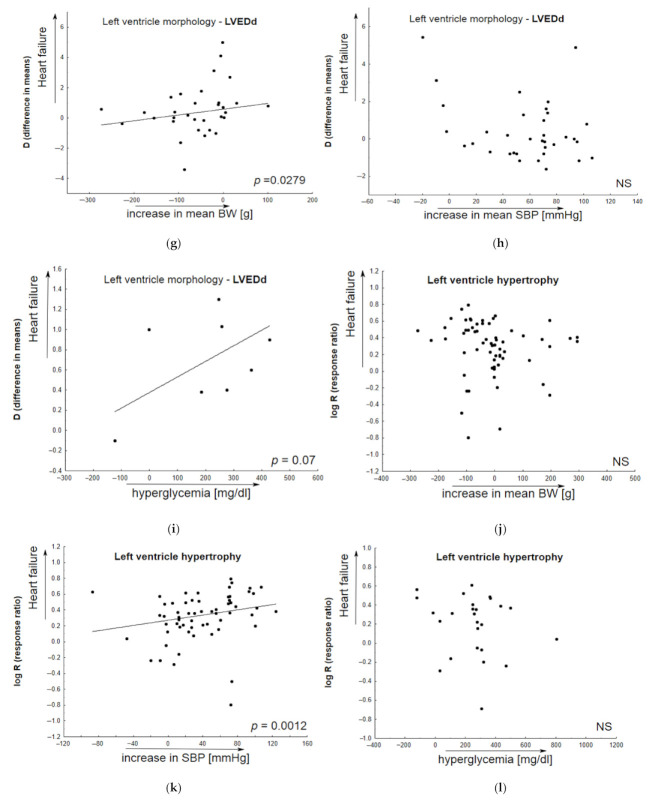
The contribution of comorbid conditions to HF development indicated by individual animal models. Any alterations in parameters associated with heart failure (Vehicle vs. healthy animals) are given as functions of the baseline body weight (systolic blood pressure, glycemia) in bubble plots; meta-regression lines have been fitted to indicate effect size. Overall active diastolic function (active relaxation) did not significantly correlate with body weight gain (**a**), or alterations in systolic blood pressure (**b**); however, it significantly decreased in animals with elevated glucose level (**c**). In addition, a decrease in passive diastolic function significantly correlated with BW gain (**d**) and higher glucose levels (**f**), but not with elevated systolic blood pressure (**e**). An increase in left ventricular end-diastolic value significantly correlated with BW gain (**g**) and higher glucose levels (**i**), but not with elevated systolic blood pressure (**h**). In HF models, LV hypertrophy insignificantly correlated with animal BW gain (**j**) or increased glucose levels (**l**), and more significantly, with SBP elevation (**k**) (N = 74 studies). Changes in BW (SBP, glycemia) were calculated as differences in the mean values of the parameter between animals with HF and healthy subjects. The overall diastolic active function was defined by such parameters as d*P*/d*t*_min_, E/A, tau; passive stiffness—by LVEDP and EDPVR slope or LVEDP/LVDd ratio.

Figure 4 presents the results of a meta-regression analysis of the worsening of particular parameters, featuring the development of heart failure as a function of body weight gain, elevated systolic blood pressure or increased glycemia. The changes in particular parameters (active diastolic relaxation: d*P*/d*t*_min_, tau, and E/A; passive stiffness: LVEDP, end-diastolic stiffness; LVEDd; LV hypertrophy) were calculated to differentiate between animals with heart failure receiving placebo (Vehicle) and healthy animals.

In most cases, the models proposed for the assessments of therapeutic agents for HF met the criteria to develop the disease based on the most common comorbidities associated with human HFpEF, viz. hyperglycemia and obesity.

### 2.4. Efficacy of Treatments for HF

Table 1 summarizes the impact of the most common medical interventions according to therapeutic group/or pharmacological class based on the normalization of heart failure; special attention is paid to parameters related to HFpEF: Active diastolic relaxation (d*P*/d*t*_min_, tau, and E/A), passive stiffness (LVEDP, end-diastolic stiffness), left ventricular end-diastolic dimension (LVEDd), pulmonary edema/lung congestion, as well as LV structure (concentric hypertrophy, LVH), and fibrosis, as well as systolic function (d*P*/d*t*_max_, LVSP). Other studies have also found the tested agents to have beneficial effects on glucose oxidation, inflammation, oxidative stress, apoptosis and mitochondrial function, as well as renal function, systolic pressure and exercise capacity, all of which are hallmarks of the deleterious functional and structural consequences of heart failure.

Table 2 presents the changes in the ejection fraction (LVEF%) observed in subjects with HF receiving individual medical intervention, the results of animal survival, and incidences of arrhythmias. The treated animals with HF were compared with their HF counterparts receiving placebos according to the particular drug, drug class, and animal model. Significant heterogeneity between particular animal models within a single pharmacological class/therapeutic group was defined as a Q measure (*p* < 0.05).

The median duration of particular drug administration was six weeks (IQR = 4; 10). A meta-regression analysis of the relationship between treatment duration and resultant efficacy of several therapeutic agents is presented in Figure S1 (Supplementary Material). The analysis considered the normalization of the following HF-related parameters: overall active diastolic function, passive stiffness, left ventricular end-diastolic dimension, pulmonary edema/lung congestion, left ventricle hypertrophy, or fibrosis.

The analyses mentioned in this Section 2.4 comprised experimental protocols in which baseline LVEF was equal to or above 50%.

### 2.5. The Resultant Efficacy of Treatments for HF in Relation to the Baseline LVEF

**Figure 5:**
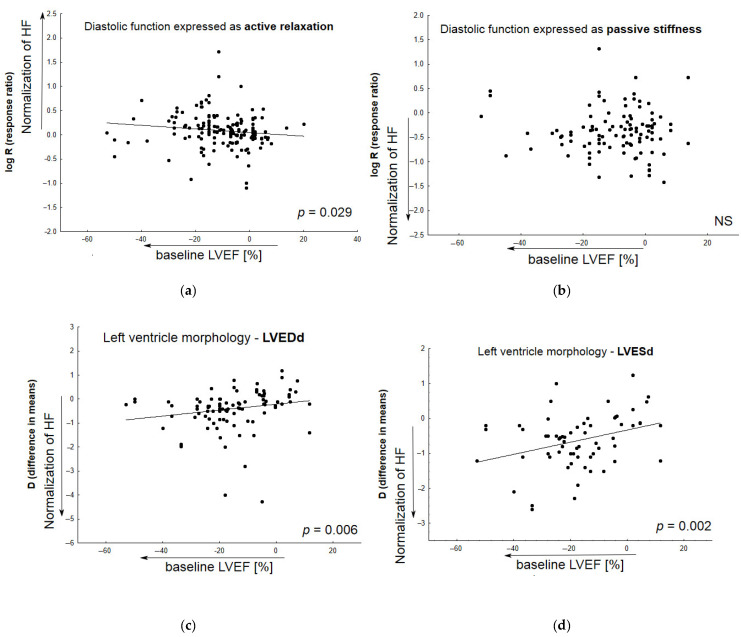

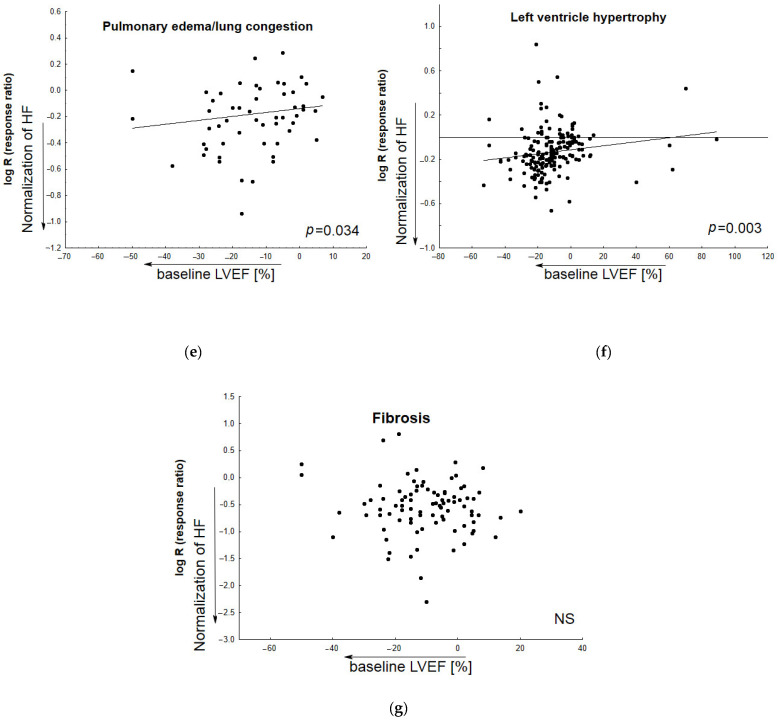
The relationship between improvement in particular HF-related parameters by tested medicine agents and baseline LVEF values in Vehicle (animals with induced heart failure). Meta-regression lines have been fitted to indicate effect size. The differences in overall passive diastolic, and fibrosis levels between animals with HF treated with pharmacological agents and those receiving placebo correlated slightly, but non-significantly, with the following: decreased baseline ejection fraction (**b**,**g**). The impact of treatment on the normalization of active diastolic function (**a**), left ventricular end-diastolic value (**c**), left ventricular end-systolic value (**d**), pulmonary edema/lung congestion (**e**), left ventricle hypertrophy (**f**) was correlated significantly with decreased baseline ejection fraction—LVEF%. (N = 131 studies). The overall diastolic active function was defined by such parameters as d*P*/d*t*_min_, E/A, tau; and passive stiffness—by LVEDP and EDPVR slope or LVEDP/LVDd ratio. NS—non significant.

Figure 5 demonstrates the relationship between the normalization of particular HF-related parameters by candidate therapeutic agents and baseline values of ejection fraction in Vehicle (animals with heart failure receiving placebo). The meta-regression analysis was performed based on inter alia active diastolic relaxation (d*P*/d*t*_min_, tau, and E/A), passive stiffness (LVEDP, end-diastolic stiffness), left ventricular end-diastolic dimension (LVEDd), pulmonary edema/lung congestion, as well as LV structure (concentric hypertrophy, LVH), and fibrosis. In most cases, animals with lower baseline LVEF values tended to respond better to the tested medication.

The potential of various models to develop HF, as indicated by baseline ejection fraction values, was analyzed based on comparisons of Vehicle animal subgroups, i.e., <50% LVEF and ≥50% LVEF. Similar subgroup analyses were performed to evaluate the chance for normalization of particular HF-lesions with regard to baseline LVEF%. Significant heterogeneity between the subgroups of animals was defined as a Q measure (*p* < 0.05). The analysis comprised the same list of HF-related parameters given above (Table S4).

## 3. Discussion

The present paper compares the efficacy of medical interventions tested in several rodent models of heart failure (HF). A wide spectrum of protocols were evaluated. These included pressure overload (transverse aortic constriction—TAC, ascending aorta constriction—ACC) and systemic hypertensive models (Dahl-salt sensitive, SHR, SHHF, subjects infused with aldosterone or angiotensin II, or receiving DOCA pellets), type 1 or 2 diabetes mellitus (STZ-treated rats or mice, and those with mutated leptin *Lep^ob/ob^* [ob/ob] or receptor for leptin *Lepr^db^* [db/db]), as well as metabolic syndrome models (ZSF1 Obese). These models were used for the evaluation of more than 150 different substances, predominantly SGLT-2 inhibitors, ARB, HMGR inhibitors, calcium sensitizers, DPP-4 inhibitors, and GLP-1 receptor agonists.

While this present systematic review indicates that a wide spectrum of therapies have positive effects on the normalization of cardiac performance, clinical observations indicate that many substances, inter alia BBs, ACEIs, ARB, MRAs, and ARNI, have negative or neutral effects on HFpEF. These agents generally failed to improve their primary outcomes (pre-specified) in their respective cardiovascular outcome trials, though some have shown potential improvements regarding secondary outcomes [3,204]. The following paragraphs discuss key considerations in the unsuccessful translation of effective animal-model-based interventions into clinical trials.

### 3.1. The Selection of Animal Models for Pre-Clinical Evaluations of Candidate Drugs

Recent findings have allowed the design of algorithms for multidimensional modeling of HFpEF in preclinical studies. According to this approach, the reviewed protocols were intended to assess their potential to develop an ejection fraction equal to or higher than 50%, diastolic dysfunction; exercise intolerance; pulmonary edema/congestion, and concentric cardiac hypertrophy [6]. While all models described in the reviewed experimental protocols evoked diastolic dysfunction, their potential to exacerbate particular HF-related parameters varied. The most comprehensive negative effect on the disease (↑passive stiffness: diastolic stiffness and LVEDP, ↑LVEDd) was observed in ZSF1 Obese, Dahl-salt sensitive rats and subjects receiving an infusion of angiotensin II; however, these did not evoke systolic dysfunction. The majority of rodent models developed LV hypertrophy, as evidenced by increased left ventricle mass with concentric lesions, as well as fibrosis.

In addition, alterations in lung mass were reported only in a quarter of all experimental protocols. Pulmonary edema/lung congestion were noted in ZSF1 Obese, Dahl salt-sensitive, SHR subjects, or those infused with angiotensin, or DOCA salt; however, this observation has not been confirmed in other protocols due to lack of data. Even fewer experiments included an assessment of the deterioration of exercise capacity. This phenomenon made it difficult to assess the degree to which a particular model used for further evaluations of effective treatments for HF could reflect human HFpEF.

In more than 20% of all reviewed protocols, Dahl-salt sensitive subjects exposed to a high salt diet (8% NaCl) were used to evaluate a large number of pharmacological agents (e.g., ACEI, ARNI, MRA, calcium sensitizers, incretins, SGLT-2 inhibitors or plant-derivatives). In addition, previous findings [6,7] indicate that ZSF1 Obese rat and DOCA-salt models, or those based on angiotensin II or aldosterone infusions, are able to mimic human HFpEF. These approaches have proven to induce diastolic dysfunction (increased EDPVR) with pulmonary congestion, LV hypertrophy, fibrosis and reduced exercise tolerance while preserving systolic function [205]. More recently, ZSF1 Obese rats were used for preclinical investigations of ARNI, ACEI, PDE-5 inhibitors, SGLT-2 inhibitors; this model made up 7% of the reviewed protocols. Animal models based on infusions of DOCA salt, aldosterone, or angiotensin II were considered by only a small number of authors.

### 3.2. Treatment Goals in Pre- and Clinical Studies

Treatment goals for patients with heart failure (HF) focus on reducing HF symptoms, improving functional capacity, enhancing quality of life, delaying the progression of the disease and reducing cardiovascular mortality. The preclinical research has mainly focused on the normalization of hemodynamic and echocardiographic parameters. Overall, the candidate therapeutic agents included in the current review improved diastolic function. The most comprehensive effect was revealed for NEP inhibitor/AT1 receptor antagonists (↓passive stiffness, ↓LVEDd). A lesser effect was noted for incretin-based medications (GLP-1 receptor agonists, and DPP-4 inhibitors), biguanides, statins and various plant-derived components. The majority of therapeutic agents normalized left ventricle hypotrophy and fibrotic lesions. While AT1/NEP inhibitors, DPP-4 inhibitors, HMGR inhibitors, MR antagonists, or PPAR-gamma agonists were all found to significantly normalize pulmonary edema/lung congestion, this parameter was assessed in only 49/194 (25%) papers.

Among the reviewed models, only SGLT-2 and PDE inhibitors demonstrated a beneficial impact on animal exercise capacity in isolated studies. The latter feature seems to be important when considering translational research in this area. While many clinical studies of cardiovascular diseases, including HFpEF, have incorporated exercise testing to quantify impairments in physiologic reserves, the protocols for preclinical studies included only assessments of resting cardiac function. Some authors emphasize the need for combining cardiac imaging or hemodynamic monitoring with exercise testing. This approach would provide an insight into the physiological reserves of the animals, and such human HFpEF symptoms as dyspnea on exertion [206]. Similarly, exercise intolerance has been recognized as a characteristic symptom of pulmonary hypertension (PH), and numerous animal exercise protocols have been established for testing novel therapies for PH. The practical implications of adaptation of exercise testing for preclinical settings in PH have been discussed more recently [207].

An analysis of mortality, a key outcome in clinical trials of effective treatments for HFpEF, was performed only in 29 papers (14.9%). Even fewer concerned the incidences of ventricular arrhythmias that could contribute to sudden death as the most common mode of death in subjects with HFpEF. Candidate drugs (e.g., SGLT-2, incretins, ivabradine, AT1 receptor antagonists) improved the survival of Dahl-sensitive rats, or subjects with HF caused by pressure overload by AAB (TAC). These approaches revealed significantly poorer survival compared with diabetic (obese) subjects, but did not reflect human HFpEF due to the transition from compensated hypertrophy to congestive heart failure [7]. On the other hand, the impact of individual drugs on animal mortality does not seem to be related with any increase in ejection fraction; this result is in line with clinical observations, where the left ventricular ejection fraction was found to be a poor indicator of prognosis for patients with HFpEF [208].

### 3.3. Comorbid Conditions

The presence of pathologic lesions in human HFrEF is largely driven by a primary defect in systolic function. Such dysfunction can be induced in animals with clinically relevant interventions, e.g., by coronary artery ligation [209]. In contrast, HFpEF has a heterogeneous pathophysiological background, including a number of comorbidities, and a single cardiac or systemic defect can fail to mimic systemic phenotypes seen in HFpEF. For this reason, the identification and evaluation of novel therapeutic approaches can be unsuccessful. The present analysis demonstrates the diverse potential of certain animal models to develop diastolic dysfunction by the most common comorbidities associated with human HFpEF. More precisely, disease worsening was most clearly demonstrated by HF parameters as LVEDP and diastolic stiffness (passive diastolic dysfunction), left ventricular end-diastolic dimension, and active relaxation (tau, E/A, d*P*/d*t*_min_)—in relation to BW gain and/or higher glucose levels. In addition, elevated systolic blood pressure was found to have a significant impact on LV hypertrophy. These phenomena concerned ZSF1 Obese animals that manifested a significant increase in body weight (by ≈206 g as compared to healthy animals), and hyperglycemia (by ≈248 mg/dL as compared to healthy animals). The potential of the model to develop a disease as a result of these conditions appears to confirm its appropriacy for evaluating drug efficacy. However, only a relatively small number of experimental protocols have been performed on this model in order to identify candidate intervention for HFpEF (<10% of the reviewed papers), and this can be seen as a limitation of the study.

### 3.4. The Role of the LVEF Parameter

As discussed above, when developing HFpEF in animals, the individual experimental protocol should result in the preservation of an ejection fraction of at least 50%. As such, papers that did not report data about LV ejection fraction (N = 111) were excluded from the analysis. More than 80% of experimental approaches displayed baseline ejection fraction values of at least 50%. These animal models were consequently included in the final analysis of the potential therapeutic efficacy of medicinal agents on HFpEF.

Interestingly, in the Vehicle group, it was possible to differentiate animals according to their potential to develop the disease and the degree to which they responded to tested agents by assuming a threshold baseline ejection fraction of 50%: for the majority of parameters, EF < 50% was associated with the development of more deleterious diseases. As a consequence, at lower baseline LVEF% values, the candidate therapeutic agents demonstrated greater disease normalization with regard to various aspects of HFpEF, including active relaxation, left ventricular end-diastolic dimension, pulmonary edema/lung congestion, or left ventricle hypertrophy. In a particular model, in untreated subjects with HF, preserving LVEF seems to favor the acquisition of reproducible and repeatable results for individual medicines.

### 3.5. Molecular Aspects of HFpEF Models

Animal models provide a valuable insight into the underlying molecular mechanisms of the disease. This phenomenon might concern HFpEF, a multisystem disorder that also involves the kidneys, skeletal muscle, adipose tissue, and immune and inflammatory signaling [210]. Activated inflammatory cascades and endothelial dysfunction have been found to promote various features of HFpEF, such as cardiomyocyte stiffening and myocardial fibrosis [211]. As summarized in Table 1, the most comprehensive beneficial impact on the subcellular hallmarks in the experimental rodent models was attributed to SGLT-2 inhibitors, sartans, incretins and HMG-CoA reductase inhibitors. These medications decreased heart failure-associated glucotoxicity and ROS generation by the vascular endothelium, and enhanced endothelial NO synthase (eNOS) activity, and thus nitric oxide (NO) bioavailability. The candidate drugs for HF demonstrated various anti-inflammatory effects, including decreased macrophage infiltration, and reduced expression of E-selectin, NF-kB and pro-inflammatory cytokines. As described previously, progressive deterioration in heart function is associated with an ongoing process of apoptosis, and these changes could be significantly modulated by candidate therapeutic agents. The above effects were reported in a small number of papers, but attributed to different HF models. Nevertheless, it is possible that HFpEF modeling may represent a unique approach for evaluating the deleterious functional and structural consequences of the disease at the molecular level, and for identifying potential specific drug therapies.

### 3.6. Study Limitations

As demonstrated above, individual pharmacological agents had different effects on LV ejection fraction, the most commonly reported parameter, according to particular animal models. However, due to the limited number of studies addressing particular drugs (pharmacological class), the variety of HF models used in the experiments, as well as the wide spectrum of the reported parameters, it was not possible to make similar comparisons to those made for LVEF%. Such comparisons might include the influence of an individual therapeutic agent (class) on the normalization of a particular parameter associated with diastolic dysfunction, according to the animal model. This phenomenon is one of the main limitations of the present study.

Another limitation is that, due to the limited number of experiments, our analyses did not include any evaluation of the potential advantages of two-hit approaches with regard to therapeutic interventions, e.g., high-fat diet plus ApoE−/−; Ang II infusion; L-NAME; or STZ, or combinations such as ZDF plus aortic constriction or Zucker fatty rat plus 6%NaCl. This requires further study. In addition, the efficacy of combined treatments for HF were not studied in these models for the same reason.

## 4. Materials and Methods

### 4.1. Article Search and Data Extraction

The PubMed and Web of Science databases were searched for papers published from January 1992 to May 2022. The search encompassed experimental protocols investigating the influence of medicinal agents on the improvement of heart failure, with special attention paid to heart failure with preserved ejection fraction, in animal models of the disease. Only protocols specifying LVEF were included, as this has been used to classify subjects into HF with preserved ejection fraction in a range of preclinical and clinical settings (Supplementary Material S2). A comprehensive search strategy was applied based on the general characteristics of the animal model of heart failure, its potential to develop features of HFpEF, and the therapeutic efficacy of the tested agents. Papers were excluded based on the following criteria: experimental protocols not including Sham (healthy animals); subjects other than rodents; protocols intended to induce acute heart failure or cardiotoxicity, or tested agents administered acutely; lack of relevant data (e.g., values of LV ejection fraction (%), number of animals). In addition, no reviews, in silico, in vitro, ex vivo or clinical studies were considered.

The recorded data included, according to the PICO acronym, animal age at the start of experiment, sex, species, background strain, characteristics of HF model (*population*); drug, route of administration, dosage, treatment duration (*therapeutic intervention*); placebo control, other medicine agent (*comparator*); and *outcomes*. The extracted *outcome* data included body weight (BW) gain (loss), left ventricle (LV) weight, LV weight/BW, LV weight/tibia length (TL), lung weight, lung weight/BW, lung weight/tibia length (TL), lung wet/dry ratio. The laboratory parameters included blood glucose (GLU), insulin (INS), total cholesterol (TC) and triglyceride (TG) level. The following hemodynamic data were recorded: systolic blood pressure (SBP), left ventricular systolic pressure (LVSP), left ventricular end-diastolic pressure (LVEDP) and time needed for relaxation of 50% maximal left ventricular pressure to baseline (tau). Echocardiographic parameters included left ventricular ejection fraction (LVEF), left ventricular end-diastolic diameter (LVEDd), left ventricular end-systolic diameter (LVESd), ratio of E-wave to A-wave (E/A), maximal rate of pressure rise (d*P*/d*t*_max_) and pressure fall (d*P*/d*t*_min_). The degree of LV fibrosis (percentage of collagen fraction, fibrosis area, interstitial or perivascular fibrosis) were extracted as histopathological data, as described above [7].

The outcome measure was extracted as mean with standard deviation, or standard error of the mean, and number of subjects per group. All studies compared the changes in hemodynamic or electrocardiographic parameters, according to LV function and structure, between one cohort of animals featuring heart failure (Vehicle), another cohort of healthy animals (Sham) and another cohort receiving particular therapeutic agents (Treatment). The literature was searched by independent reviewers (MJ-S, AK), who extracted the data and assessed the risk of bias. Any disagreements were solved by discussion until consensus was reached.

### 4.2. Quality Assessments

The risk of bias was assessed using the SYstematic Review Centre for Laboratory animal Experimentation (SYRCLE) risk of bias tool for animal studies [203]. An Egger’s weighted regression and Duval and Tweedie ‘trim and fill’ procedure were performed to evaluate publication bias across the individual studies. Further analyses in subgroups were performed according to selected variables (drug, HF model, etc.) in at least five studies per model.

### 4.3. Data Synthesis

The effect size for the Vehicle group was calculated with a 95% confidence interval (CI). Changes in the mean response (X) given by the Vehicle group (animals with HF receiving placebo, X_HF+placebo_), and healthy animals (X_healthy_) were expressed

as difference in means (D):

D = X_HF+placebo_ − X_healthy_, (1)

or were calculated as response ratio (R):

R = X_HF+placebo_/X_healthy_ (2)

The effect size for the Treatment group was calculated with a 95% confidence interval (CI). Any differences in the mean response (X) given by animals with HF receiving therapeutic agents (Treatment, X_HF+drug_) and the Vehicle group (animals with HF receiving placebo, X_HF+placebo_) were expressed

as difference in means (D):

D = X_HF+drug_ − X_HF+placebo_, (3)

or were calculated as response ratio (R):

R = X_HF+drug_/X_HF+placebo_ (4)

Every comparison between Vehicle vs. Sham, or Treatment vs. Vehicle was regarded as a separate ‘intervention’.

In most cases, changes in echocardiographic, hemodynamic parameters, as well as in animal body weight or laboratory parameters were analyzed using Formula (1) for the Vehicle group, or Formula (3) for the Treatment group. An increase in D (difference in means), indicates a rise in the value of a particular, HF-related, parameter in the Vehicle group as compared to healthy subjects (*worsening of the disease*), while a decrease in D indicates a reduced value of a particular parameter in the Treatment group as compared to the Vehicle group (*normalization of the disease*).

Some of these components were used to define ↑LV mass, fibrosis, overall diastolic function, or passive stiffness. Then, any differences in these parameters between Vehicle (subjects with HF) and healthy rodents were calculated using Formula (2), and differences between Vehicle and Treatment group were calculated using Formula (4). A response ratio (R) of 1 denotes no dissimilarities between Vehicle and healthy subjects, or between the Treatment group and Vehicle group. Values R > 1 (R < 1) designate an increase (or a reduction) in a particular parameter in a respective HF model [7]. As the primary aim of the analysis was related to the effects of particular treatments for HF, the comparative analyses of publication bias were performed according to the Formulas (3) and (4).

Meta-regression and subgroup analyses were performed where possible to evaluate the influence of particular variables (e.g., procedure to introduce HF, animal species, medicine agent/pharmacological class, treatment duration) on the outcome. The statistical analyses were performed using a random-effects model (STATISTICA 13.1). Heterogeneity between two (or more) animal subgroups was indicated by a statistically significant Cochran’s Q score. A significant result was indicted by a *p*-value < 0.05.

## 5. Conclusions

There remains a need for comprehensive preclinical HFpEF models employing physiological stress-based functional testing approaches that can mimic human diseases characterized by a multi-organ phenotype; this need is particularly pressing considering the growing incidence of HFpEF in aging populations and the lack of targeted therapies. Existing preclinical studies in HFpEF have identified a wide spectrum of candidate agents that have been found to be effective against LV diastolic performance. Their activity has been evaluated in a variety of experimental protocols, most of which are based on pressure overload or systemic hypertension, with a smaller number examining type 1 or 2 diabetes mellitus, or obesity; however, some of these models could fail to reflect human disease.

In contrast, fewer studies have assessed pulmonary congestion, exercise capacity, arrhythmia, and animal mortality, as well as certain molecular hallmarks such as inflammation, oxidative stress and endothelial dysfunction. This gap may partially explain why traditional medical agents have been found to demonstrate negative or neutral effects in clinical trials, despite having positive outcomes in experimental studies. Other potential explanations may be:

(a) Experimental approaches are characterized with varying degrees of effectiveness regarding their ability to develop the disease by the most common comorbidities associated with human HFpEF (hypertension, hyperglycemia, and/or obesity). The ZSF1 model is able to develop disease by a similar set of conditions to humans; as such, it appears suitable for identifying candidate medications.
(b) The minority of experiments concerned drug-related normalization of pulmonary congestion, and even fewer entailed the improvement of animal exercise capacity. Both features represent treatment goals for patients with HF, and this phenomenon can make it difficult to comprehensively evaluate effective treatments for human HFpEF.
(c) The baseline ejection fraction can play a key role in determining the extent to which particular medicine agents normalized the disease according to diastolic dysfunction, LV hypertrophy, or pulmonary congestion. In a particular model, preserving LVEF seems to favor the acquisition of reproducible and repeatable results for individual medicines.
(d) The mortality associated with a candidate agent can be determined based on the likelihood of an individual experimental model to worsen animal survival. This should be taken into consideration in studies comparing different therapeutic agents.
(e) Animal models of HFpEF might represent a promising approach for evaluating the deleterious functional and structural consequences of the disease at the molecular level, and for identifying future specific drug therapies. However, these effects were reported in only a small number of papers.

## Figures and Tables

**Table 1:** The summary of the impact of the most common agents according to the therapeutic groups/pharmacological classes on HF in animal models as evaluated in the reviewed study protocols, with special attention to the systolic function (d*P*/d*t*_max_, LVSP, LVEF), active diastolic relaxation (d*P*/d*t*_min_, tau, E/A), passive stiffness (LVEDP, end-diastolic stiffness), left ventricular end-diastolic dimension (LVEDd), LV structure (concentric hypertrophy, LVH), fibrosis, or pulmonary edema/lung congestion. The model most often used in research on an individual group was bolded.

Pharmacologic Class/Therapeutic Group	Active Substance	Species	Animal Model *	Systolic Function	Diastolic Function		LVH	LVEDd	Fibrosis	Pulmonary Edema/Lung Congestion	Other Parameters		Refs
					Active Relaxation	Passive Stiffness (LVEDP, Diastolic Stiff)					Hemodynamics, Renal Function, Exercise	Biochemical Markers	
ACE inhibitor	Captopril, Benazepril, Temocapril	rat	Dahl salt sensitive, ZSF1 Obese	↔LVEF [%]; ↑d*P*/d*t*_max_	↑E/A; ↑d*P*/d*t*_min_	↓		↔	↓		↓SBP; ↑Ccr	↓NT-proBNP	[17,85,137,138]
AT1 receptor antagonist (ARB)	Irbesartan, Losartan, Olmesartan, Telmisartan, Valsartan	mouse/ rat	Ang II, Dahl salt sensitive, pressure-overload by AAB (TAC), SHR, STZ, ZSF1 Obese	↑LVEF [%]; ↑LVSP	↑d*P*/d*t*_min_	↔	↓	↔	↓		↓SBP; ↓Cr	↓NT-proBNP (BNP); ↓Infl; ↑MF; ↓FA; ↓Apoptosis	[15,17,21,30,70,85,86,96,149,160,164,170,197,200]
Beta-blocker (BB)	Bisoprolol, BRL 37,344, Metoprolol, Nebivolol, Propranolol	mouse/ rat	Ang II, Dahl salt sensitive, pressure-overload by AAB (TAC)	↑LVEF [%]	↑E/A; ↑tau	↓	↓*p* = 0.0957		↓	↓ *p* = 0.0792	↓SBP; ↑Cr		[59,81,82,127]
MR antagonist (MRA)	Eplerenone, Finerenone, Spironolactone	mouse/ rat	Dahl salt sensitive, pressure overload by TAC, SHR, SHHF aged, SHHF cp/cp obese	↑LVEF [%]	↑E/A		↓	↔	↓	↓		↓BNP; ↓Infl	[28,58,118,123,184]
NEP inhibitor/AT1 receptor antagonist (ARNI)	Sacubitril/Valsartan	mouse/ rat	Pressure-overload by AB (TAC), SHR, ZSF1 Obese	↑LVEF [%]		↓	↓	↓	↓	↓		↑Endothelium	[24,122,140,149]
Biguanide	Metformin	mouse/ rat	Ang II, db/db, pressure overload by ACF, STZ + HFD	↑LVEF [%]	↑tau	↔	↓	↓	↓	↓ *p* = 0.0776		↓GLU; ↓Apoptosis	[20,151,154,188]
DPP-4 inhibitor	Saxagliptin, Sitagliptin, Teneligliptin	mouse/rat/	Dahl salt sensitive, db/db, Isoproterenol-induced HF	↑LVEF [%]; ↑d*P*/d*t*_max_; ↔LVSP	↑E/A; ↑d*P*/d*t*_min_ ↑tau	↓	↓	↔	↓	↓		↓Infl; ↓OX; ↑Endothelium	[45,61,73,179]
GLP-1 receptor agonist	Exendin-4, GLP-1, Liraglutide	rat/ hamster	Dahl salt sensitive, pressure-overload by AAB, STZ, Zucker fatty rat	↑LVEF [%]; ↑LVSP	↑E/A *p* = 0.073	↓	↓	↓	↔	↔		↓Infl; ↓OX; ↓NT-proBNP; ↓Apoptosis	[18,119,120,145,148,196,200]
PPAR-gamma agonist	Ciglitazone, Pioglitazone, Rosiglita-zone	mouse/ rat	Pressure-overload by AAB (ACF), ZDF + TAC	↔LVEF [%]; ↔LVSP; ↑ d*P*/d*t*_max_ *p* = 0.06		↔		↓		↓	↓SBP	↑Endothelium	[50,52,64,79]
SGLT-2 inhibitor	Dapagliflozin, Empagliflozin	mouse/ rat	Ang II, Dahl salt sensitive, db/db (ob/ob), L-Name + HFD, pressure-overload by TAC, SHHF, SHR, STZ (+HFD), ZSF1 Obese	↑LVEF [%]	↑d*P*/d*t*_min_	↔	↓	↔	↓	↔	↓SBP; ↑Exercise	↑Endothelium; ↓OX;↓Infl; ↑GLU; ↓BNP	[10,16,25,27,60,62,69,79,88,91,110,126,154,158,178,191]
Calcium sensitizer	Levosi mendan, OR-1986	rat	Dahl salt sensitive	↑LVEF [%]			↔		↓		↓SBP	↓BNP; ↓Apoptosis	[21,102,103]
HMGR inhibitor	Atorvastatin, Pravastatin, Rosuvastatin	mouse/ rat	Dahl salt sensitive, pressure overload by AAB (TAC), SHHF, SHR	↑LVEF [%]; ↔LVSP; ↑d*P*/d*t*_max_		↓	↓	↓	↔	↓		↓Infl; ↑Endothelium; ↓OX; ↓Apoptosis; ↓NT-proBNP	[12,31,54,94,100,131,193]
I(f) current inhibitor	Ivabradin	mouse/ rat	Dahl salt sensitive, pressure overload by TAC	↑LVEF [%]	↑d*P*/d*t*_min_	↓	↓ *p* = 0.052	↓	↔	↓		↓Infl; ↓Apoptosis	[73,135,194]
Immunomodulators	5-azacytidine, Carvacrol, FTY-720, ITF2357/Givinostat, MCC950, PCI34051, Rituximab	mouse	Dahl salt sensi-tive, db/db + STZ, DOCA salt, TAC	↑LVEF [%]	↑d*P*/d*t*_min_	↓	↓	↓				↓BNP; ↓Infl	[66,76,97,108,136,155,194,196]
PDE3-i, PDE5-i, PDE9-i	Cilostazol, Sildenafil, Vardenafil, PF-4449613, CRD-733	mouse/ rat	Ang II(+HFD), db/db, DOCA salt (+TAC), ISO + TAC, pressure overload by TAC, ZSF1, ZDF	↔LVEF [%]; ↑d*P*/d*t*_max_		↓	↔		↓	↓ *p* = 0.0786	↑Exercise	↓OX; ↓Apoptosis; ↓Infl	[55,56,89,111,113,133,134,163,183]
Plant-derivatives	See below #	mouse/ rat	Dahl salt sensitive, pressure overload by AAB (TAC), db/db (+STZ), L-Name + HFD, SHHF, SHR, STZ	↑LVEF [%]; ↔LVSP	↑d*P*/d*t*_min_	↓	↓	↓	↓			↓Apoptosis; ↑Endothelium; ↑MF; ↓OX	[13,32,33,34,35,36,49,67,80,84,92,98,101,135,152,171,173,180,181,182,185,188,201]
XO inhibitor	Allopurinol, Febuxostat, Oxypurinol	mouse/ rat	Pressure overload by ACF (TAC)	↔LVEF [%]		↓		↓				↓OX	[51,114,175,176]

*—baseline mean ejection fraction was equal or higher than 50% in heart failure group (Vehicle); AAB—Abdominal Aortic Banding; AB—Aortic Banding; ACE—Angiotensin Converting Enzyme; ACF—Aortocaval Fistula; Ang—Angiotensin; AT1—Angiotensin II Receptor Type 1; Ccr—creatinine clearance; Cr—serum creatinine; DOCA—Deoxycorticosterone Acetate; d*P*/d*t*_max_—maximal rate of pressure rise; d*P*/d*t*_min_—maximal rate of pressure fall; DPP4—Dipeptidyl Peptidase-4; E/A—ratio of E-wave to A-wave; EF—ejection fraction; Endothelium—endothelium function; FA—fatty acid oxidation; GLP-1—Glucagon-Like Peptide 1; GLU—glucose oxidation; HFD—high fat diet; HMGR—(HMG-CoA) Reductase; Infl—Inflammation as macrophage infiltration, expression of E-selectin, NF-kB activity, or expression of pro-inflammatory cytokines; L-NAME—L-NG-Nitro arginine methyl ester; LVEDd—left ventricular end-diastolic dimension; LVEDP—left ventricular end-diastolic pressure; LVH—left ventricular hypertrophy; LVSP—left ventricular systolic pressure; MF—mitochondrial function as activation of AMPK signaling; MR—Mineralocorticoid-Receptor; NEP—Neprilysin; OX—oxidative stress; PDE—Phosphodiesterase; PPAR—Peroxisome Proliferator-Activated Receptor; RAAS—Renin-Angiotensin-Aldosterone system; SBP—systolic blood pressure; SGLT2—Sodium-glucose co-transporter-2; SHHF—Spontaneously Hypertensive Heart Failure; SHR—Spontaneously Hypertensive Rat; stiff—stiffness; STZ—Streptozotocin; TAC—Transverse Aortic Constriction; tau—time needed for relaxation of 50% maximal left ventricular pressure to baseline; XO—Xanthine Oxidase; ZDF—Zucker Diabetic Fatty; #—3′,4′-Dihydroxyflavonol, Apocynin, Carvacrol, Cinnamaldehyde, Cucurbitacin B, Dihydromyricetin, Epigallocatechin-3 gallate, Gallic acid, Gnetol, Icariside II, Luteolin, Moringa oleifera seed powder, Nobiletin, Nuanxin, Oleanolic acid, Paeonol, Pterostilbene, Puerarin, Qiliqiangxin, QiShenYiQi (QSYQ), Quercetin, Resveratrol, Sanggenon C, Thymoquinone, Xiao-Qing-Long-Tang; ↓ (↑)—statistically significant decrease (increase) in particular parameter according to the results of meta-analysis except for section: Other parameters.

**Table 2:** The alterations in left ventricular ejection fraction (LVEF%), arrhythmias and mortality of subjects with HF receiving candidate therapeutic agents, per drug, drug class and animal model.

Pharmacologic Class/Therapeutic Group	Active Substance	Animal Model *	Effect	Diff in Means (95%CI)	Comparison **	Arrhythmia; Mortality [Refs]
ACE inhibitor	Captopril, Benazepril, Temocapril	Dahl salt sensitive	↔	4.28 (−5.39; 13.96); NS	-	
AT1 receptor antagonist (ARB)	Irbesartan, Losartan, Olmesartan, Telmisartan, Valsartan	Ang II	↑	3.00 (0.52; 5.48); *p* < 0.0001	Q = 126.88; df = 5; *p* < 0.0001	
		Dahl salt sensitive	↔	−1.52 (−4.24; 1.18); NS		↓M [21,86]
		Pressure−overload by AAB (TAC)	↑	12.72 (7.86; 17.58); *p* < 0.0001		
		SHR	↔	1.01 (−2.86; 4.87); NS		
		STZ	↓	−6.70 (−9.44; −3.95); *p* < 0.0001		
		ZSF1 Obese	↑	11.00 (8.76; 13.23); *p* < 0.0001		
Beta-blocker (BB)	Bisoprolol, BRL 37344, Metoprolol, Nebivolol, Propranolol	Ang II	↑	9.9 (8.23; 11.57); *p* < 0.0001	Q = 76.04; df = 2; *p* < 0.0001	
		Dahl salt sensitive	↔	5.79 (−0.75; 21.94); NS		↓M [81,127]
		Pressure-overload by AAB (TAC)	↔	1.00 (−0.13; 2.13); NS		
MR antagonist (MRA)	Eplerenone, Finerenone, Spironolacto ne	Dahl salt sensitive	↔	−2.45 (−5.49; 0.59); NS	Q = 24.96; df = 2; *p* < 0.0001	
		SHR	↑	2.00 (1.43; 2.57); *p* < 0.0001		
		SHHF aged (cp/cp obese)	↑	10.15 (6.25; 14.06); *p* < 0.0001		↓M [183]
NEP inhibitor/AT1 receptor antagonist (ARNI)	Sacubitril/Valsartan	Pressure-overload by AB (TAC)	↔	10.50 (−8.12; 29.12); NS	Q = 4.10; df = 2; NS	↓M [24]
		SHR	↔	−1.00 (−9.16; 7.16); NS		↓A [149]
		ZSF1 Obese	↑	8.48 (3.79; 13.18); *p* = 0.0004		
Biguanide	Metformin	Ang II inf	↑	10.00 (7.27; 12.72); *p* < 0.0001	Q = 136.59; df = 3; *p* < 0.0001	
		db/db	↑	15.00 (13.04; 16.96); *p* < 0.0001		
		Pressure overload by ACF	↑	6.2 (3.25; 9.15); *p* < 0.0001		
		STZ + HFD	↑	26.00 (23.74; 28.26); *p* < 0.0001		
DPP-4 inhibitor	Saxagliptin, Sitagliptin, Teneligliptin	Dahl salt sensitive	↔	−0.95 (−2.73; 0.83); NS	Q = 353.67; df = 2; *p* < 0.0001	↓M [45]
		db/db	↑	19.9 (14.05; 25.75); *p* < 0.0001		
		Isoproterenol-induced HF	↑	18.7 (17.66; 19.74); *p* < 0.0001		
GLP-1 receptor agonist	Exendin-4, GLP-1, Liraglutide	Dahl salt sensitive	↔	0.01 (−1.76; 1.75); NS	Q = 21.24; df = 3; *p* < 0.0001	↔M [119]
		Pressure-overload by AAB	↔	7.08 (−2.32; 16.49); NS		↓M [120]
		STZ	↑	5.32 (3.57; 7.07); *p* < 0.0001		↓A [195]
		Zucker fatty rat	↔	1.13 (−0.35; 2.61); NS		
PPAR-gamma agonist	Ciglitazone, Pioglitazone, Rosiglitazone	Pressure-overload by AAB (ACF)	↔	9.18 (−4.53; 22.90); NS	Q = 1.48; df = 1; NS	
		ZDF + TAC	↔	−0.22 (−6.69; 6.24); NS		
SGLT-2 inhibitor	Dapagliflozin, Empagliflozin	Ang II	↓	−2.98 (−4.69; −1.27); *p* = 0.0007	Q = 1013.1; df = 8; *p* < 0.0001	
		Dahl salt sensitive	↔	−0.57 (−1.75; 0.60); NS		
		db/db (ob/ob)	↑	5.83 (0.01; 11.67); *p* = 0.049		
		L−Name + HFD	↓	−2.00 (−3.95; −0.04); *p* = 0.045		
		Pressure-overload by TAC	↑	7.92 (3.73; 15.23); *p* = 0.035		↓M [91]
		SHHF	↔	0.01 (−2.27; 2.27): NS		
		SHR	↔	−1.00 (−4.99; 2.99): NS		
		STZ (+HFD)	↑	13.19 (2.02; 24.37); *p* = 0.03		
		ZSF1 Obese	↔	5.00 (−6.31; 16.32); NS		
Calcium sensitizer	Levosime ndan, OR-1986	Dahl salt sensitive	↑	7.58 (5.46; 9.70); *p* < 0.0001	-	↓M [102,103]
I(f) current inhibitor	Ivabradin	Dahl salt sensitive	↑	15.9 (9.31; 22.49); *p* < 0.0001	Q = 9.18; df = 1; *p* = 0.002	↓M [21,81]
		Pressure overload by TAC	↔	2.4 (−3.32; 8.13); NS		
Immunomodulators	FTY-720, MCC950	Pressure overload by TAC	↔	21.66 (−1.49; 44.81); *p* = 0.06	-	[97,194]
HMGR inhibitor	Atorvastatin, Pravastatin, Rosuvastatin	Dahl salt sensitive	↔	−1.00 (−7.03; 5.03); NS	Q = 32.28; df = 3; *p* < 0.0001	
		Pressure overload by AAB (TAC)	↑	18.82 (14.53; 23.10); *p* < 0.0001		
		SHHF	↔	4.00 (−2.93; 10.93); NS		
		SHR	↔	7.67 (−0.08; 15.43); NS		
PDE3-i, PDE5-i, PDE9-i	Cilostazol, CRD-733, PF-4449613, Sildenafil, Vardenafil,	Ang II inf (+HFD)	↔	1.00 (−2.73; 4.73); NS	Q = 65.22; df = 5; *p* < 0.0001	
		DOCA salt (+TAC)	↓	−8.5 (−11.44; −5.56): *p* < 0.0001		
		ISO + TAC	↑	10.32 (6.74; 13.91): *p* < 0.0001		
		Pressure overload by TAC	↔	6.40 (−14.87; 27.67); NS		
		ZDF	↔	−2.00 (−5.14; 1.14): NS		
		ZSF1 Obese	↔	0.01 (−7.07; 7.06): NS		
Plant-derivatives	As listed below#	Dahl salt sensitive	↑	9.28 (4.60; 13.96); *p* < 0.0001	Q = 45.25; df = 6; *p* < 0.0001	↓M (RES) [119]
		db/db (+STZ)	↑	8.78 (4.95; 12.62); *p* < 0.0001		
		L-Name+ HFD	↔	2.51 (−6.97; 12.00); NS		
		Pressure overload by AAB (TAC)	↓	−2.00 (−3.96; −0.04); *p* = 0.046		
		SHHF	↑	4.62 (2.41; 6.83); *p* < 0.0001		
		SHR	↔	3.14 (−9.10; 15.38); NS		
		STZ	↑	6.96 (2.16; 11.77); *p* = 0.0044		
XO inhibitor	Allopurinol, Febuxostat, Oxypurinol	Pressure overload by ACF (TAC)	↔	3.97 (−3.73; 11.66); NS	-	↑M (ALLO) [177]

*—mean ejection fraction was equal or higher than 50% in heart failure group (Vehicle); **—statistically significant Q measure (*p* < 0.05) indicates the difference among particular models within a single pharmacological class (therapeutic group). AAB—Abdominal Aortic Banding; AB—Aortic Banding; ACE—Angiotensin Converting Enzyme; ACF—Aortocaval Fistula; Ang—Angiotensin; AT1—Angiotensin II Receptor Type 1; DOCA—Deoxycorticosterone Acetate; d*P*/d*t*_max_—maximal rate of pressure rise; d*P*/d*t*_min_—maximal rate of pressure fall; DPP4—Dipeptidyl Peptidase-4; E/A—ratio of E-wave to A-wave; EF—ejection fraction; GLP-1—Glucagon-Like Peptide 1; HFD—high fat diet; HMGR—(HMG-CoA) Reductase; L-NAME—L-NG-Nitro arginine methyl ester; LVEDd—left ventricular end-diastolic dimension; LVEDP—left ventricular end-diastolic pressure; LVH—left ventricular hypertrophy; LVSP—left ventricular systolic pressure; MR—Mineralocorticoid-Receptor; NEP—Neprilysin; NS—non-significant; PDE—Phosphodiesterase; PPAR—Peroxisome Proliferator-Activated Receptor; RAAS—Renin-Angiotensin-Aldosterone system; SGLT2—Sodium-glucose co-transporter-2; SHHF—Spontaneously Hypertensive Heart Failure; SHR—Spontaneously Hypertensive Rat; stiff—stiffness; STZ—Streptozotocin; TAC—Transverse Aortic Constriction; tau—time needed for relaxation of 50% maximal left ventricular pressure to baseline; XO—Xanthine Oxidase; ZDF—Zucker Diabetic Fatty; #—3′,4′-Dihydroxyflavonol, Apocynin, Carvacrol, Cinnamaldehyde, Cucurbitacin B, Dihydromyricetin, Epigallocatechin-3 gallate, Gallic acid, Gnetol, Icariside II, Luteolin, Moringa oleifera seed powder, Nobiletin, Nuanxin, Oleanolic acid, Paeonol, Pterostilbene, Puerarin, Qiliqiangxin, QiShenYiQi (QSYQ), Quercetin, Resveratrol, Sanggenon C, Thymoquinone, Xiao-Qing-Long-Tang; A—arrhythmia; ALLO—allopurinol; M—mortality; RES—resveratrol; ↑(↓, ↔)—alterations in systolic function expressed as LVEF; the data about animal survival were provided according to analysis of Kaplan–Meyer curves that were presented in individual papers.

## Data Availability

Data is contained within the article and Supplementary Material.

## Supplementary Materials

The following supporting information can be downloaded at: https://www.mdpi.com/article/10.3390/ph16101449/s1, Supplementary Material S1: Study characteristics report and additional results. Figure S1: The relationship between improvement in particular HF-related parameters- by tested medicine agents and drug administration period. Meta-regression lines have been fitted to indicate effect size. No significant correlation between length of medicine agent administration and alterations in particular parameters in treated animals with HF as compared to the Vehicle (animals with HF reviving placebo) was not denoted. Overall active diastolic function (**a**); passive stiffness (**b**); left ventricular end-diastolic dimension (**c**); pulmonary edema/lung congestion (**d**); left ventricle hypertrophy (**e**); fibrosis (**f**) (N = 74 studies). Mean ejection fraction was equal or higher than 50% in heart failure group (Vehicle); Table S1: Study characteristics report; Table S2: Results from publication bias funnel plot and ‘trim and fill analysis’; Table S3: Detailed characteristics of animal models used for experimental protocols to evaluate efficacy of particular medicine agent for HF. Increased (D > 0, or R > 1) values for comparison Vehicle vs. Sham indicate worsening (W) of HF in relation to healthy subjects (Sham); decreased (D < 0, or R < 1) values—indicate weaker potential of animal model to promote disease development, according to particular parameter. D—difference in means; R—response ratio; Ⱦ—mean EF in Vehicle group. * studies where baseline mean ejection fraction was equal or higher than 50% were considered, only; Table S4: The impact of baseline ejection fraction in Vehicle animals on the worsening/normalization of HF-related parameters in subjects with HF receiving placebo (Vehicle) and medicinal agents (Treatment). Increased (D > 0, or R > 1) values for comparison: Vehicle vs. Sham indicate worsening (W) of HF in relation to healthy subjects (Sham); decreased (D < 0, or R < 1) values for comparison Treatment vs. Vehicle indicate more normalization (N) of the disease in relation to HF subjects receiving placebo (Vehicle). For the majority of parameters, except from active relaxation, fibrosis and LVSP, the development of more deleterious disease was accompanied by the baseline LVEF below threshold of 50%; similarly, the medicinal agents normalized the disease more in subjects with LVEF < 50%. Statistically significant Q measure (*p* < 0.05) indicates the difference between groups (i.e., <50% vs. ≥50%). Table S5: PRISMA 2020 Checklist. Supplementary Material S2: Search Criteria.

## References

[1] Van Riet E.E., Hoes A.W., Wagenaar K.P., Limburg A., Landman M.A., Rutten F.H. (2016). Epidemiology of heart failure: The prevalence of heart failure and ventricular dysfunction in older adults over time. A systematic review. Eur. J. Heart Fail..

[2] Owan T.E., Hodge D.O., Herges R.M., Jacobsen S.J., Roger V.L., Redfield M.M. (2006). Trends in prevalence and outcome of heart failure with preserved ejection fraction. N. Engl. J. Med..

[3] Del Buono M.G., Iannaccone G., Scacciavillani R., Carbone S., Camilli M., Niccoli G., Borlaug B.A., Lavie C.J., Arena R., Crea F. (2020). Heart failure with preserved ejection fraction diagnosis and treatment: An updated review of the evidence. Prog. Cardiovasc. Dis..

[4] Borlaug B.A. (2014). The pathophysiology of heart failure with preserved ejection fraction. Nat. Rev. Cardiol..

[5] Conceição G., Heinonen I., Lourenço A.P., Duncker D.J., Falcão-Pires I. (2016). Animal models of heart failure with preserved ejection fraction. Neth. Heart J..

[6] Valero-Muñoz M., Backman W., Sam F. (2017). Murine Models of Heart Failure with Preserved Ejection Fraction: A “Fishing Expedition”. JACC Basic Transl. Sci..

[7] Jasińska-Stroschein M. (2022). Searching for an experimental rodent model of heart failure with preserved ejection fraction: Revisited. Biomed. Pharmacother..

[8] Barandiarán Aizpurua A., Schroen B., van Bilsen M., van Empel V. (2018). Targeted HFpEF therapy based on matchmaking of human and animal models. Am. J. Physiol.-Heart Circ. Physiol..

[9] Abdellatif M., Trummer-Herbst V., Koser F., Durand S., Adão R., Vasques-Nóvoa F., Freundt J.K., Voglhuber J., Pricolo M.R., Kasa M. (2021). Nicotinamide for the treatment of heart failure with preserved ejection fraction. Sci. Transl. Med..

[10] Abdurrachim D., Teo X.Q., Woo C.C., Chan W.X., Lalic J., Lam C.S.P., Lee P.T.H. (2019). Empagliflozin reduces myocardial ketone utilization while preserving glucose utilization in diabetic hypertensive heart disease: A hyperpolarized 13 C magnetic resonance spectroscopy study. Diabetes Obes. Metab..

[11] Adams V., Schauer A., Augstein A., Kirchhoff V., Draskowski R., Jannasch A., Goto K., Lyall G., Männel A., Barthel P. (2022). Targeting MuRF1 by small molecules in a HFpEF rat model improves myocardial diastolic function and skeletal muscle contractility. J. Cachexia Sarcopenia Muscle.

[12] Akahori H., Tsujino T., Naito Y., Matsumoto M., Sasaki N., Iwasaku T., Eguchi A., Sawada H., Hirotani S., Masuyama T. (2014). Atorvastatin ameliorates cardiac fibrosis and improves left ventricular diastolic function in hypertensive diastolic heart failure model rats. J. Hypertens..

[13] Akinwumi B.C., Raj P., Lee D.I., Acosta C., Yu L., Thomas S.M., Nagabhushanam K., Majeed M., Davies N.M., Netticadan T. (2017). Disparate Effects of Stilbenoid Polyphenols on Hypertrophic Cardiomyocytes In Vitro vs. in the Spontaneously Hypertensive Heart Failure Rat. Molecules.

[14] Altara R., da Silva G.J.J., Frisk M., Spelta F., Zouein F.A., Louch W.E., Booz G.W., Cataliotti A. (2020). Cardioprotective Effects of the Novel Compound Vastiras in a Preclinical Model of End-Organ Damage. Hypertension.

[15] Aroor A.R., Mummidi S., Lopez-Alvarenga J.C., Das N., Habibi J., Jia G., Lastra G., Chandrasekar B., DeMarco V.G. (2021). Sacubitril/valsartan inhibits obesity-associated diastolic dysfunction through suppression of ventricular-vascular stiffness. Cardiovasc. Diabetol..

[16] Asensio Lopez M.D.C., Lax A., Hernandez Vicente A., Saura Guillen E., Hernandez-Martinez A., Fernandez Del Palacio M.J., Bayes-Genis A., Pascual Figal D.A. (2020). Empagliflozin improves post-infarction cardiac remodeling through GTP enzyme cyclohydrolase 1 and irrespective of diabetes status. Sci. Rep..

[17] Awwad Z.M., El-Ganainy S.O., ElMallah A.I., Khattab M.M., El-Khatib A.S. (2019). Telmisartan and captopril ameliorate pregabalin-induced heart failure in rats. Toxicology.

[18] Bai X.J., Hao J.T., Zheng R.H., Yan C.P., Wang J., Yang C.H., Zhang W.F., Zhao Z.Q. (2021). Glucagon-Like Peptide-1 Analog Liraglutide Attenuates Pressure-Overload Induced Cardiac Hypertrophy and Apoptosis through Activating ATP Sensitive Potassium Channels. Cardiovasc. Drugs Ther..

[19] Bartoli F., Bailey M.A., Rode B., Mateo P., Antigny F., Bedouet K., Gerbaud P., Gosain R., Plante J., Norman K. (2020). Orai1 Channel Inhibition Preserves Left Ventricular Systolic Function and Normal Ca^2+^ Handling After Pressure Overload. Circulation.

[20] Benes J., Kazdova L., Drahota Z., Houstek J., Medrikova D., Kopecky J., Kovarova N., Vrbacky M., Sedmera D., Strnad H. (2011). Effect of metformin therapy on cardiac function and survival in a volume-overload model of heart failure in rats. Clin. Sci..

[21] Biala A., Finckenberg P., Korpi A., Loytainen M., Martonen E., Levijoki J., Mervaala E. (2011). Cardiovascular effects of the combination of levosimendan and valsartan in hypertensive Dahl/Rapp rats. J. Physiol. Pharmacol..

[22] Bryson T.D., Pandrangi T.S., Khan S.Z., Xu J., Pavlov T.S., Ortiz P.A., Peterson E., Harding P. (2020). The deleterious role of the prostaglandin E2 EP3 receptor in angiotensin II hypertension. Am. J. Physiol. Heart Circ. Physiol..

[23] Bugyei-Twum A., Ford C., Civitarese R., Seegobin J., Advani S.L., Desjardins J.F., Kabir G., Zhang Y., Mitchell M., Switzer J. (2018). Sirtuin 1 activation attenuates cardiac fibrosis in a rodent pressure overload model by modifying Smad2/3 transactivation. Cardiovasc. Res..

[24] Burke R.M., Lighthouse J.K., Mickelsen D.M., Small E.M. (2019). Sacubitril/Valsartan Decreases Cardiac Fibrosis in Left Ventricle Pressure Overload by Restoring PKG Signaling in Cardiac Fibroblasts. Circ. Heart Fail..

[25] Byrne N.J., Matsumura N., Maayah Z.H., Ferdaoussi M., Takahara S., Darwesh A.M., Levasseur J.L., Jahng J.W.S., Vos D., Parajuli N. (2020). Empagliflozin Blunts Worsening Cardiac Dysfunction Associated With Reduced NLRP3 (Nucleotide-Binding Domain-Like Receptor Protein 3) Inflammasome Activation in Heart Failure. Circ. Heart Fail..

[26] Cao H.J., Fang J., Zhang Y.L., Zou L.X., Han X., Yang J., Yan X., Li P.B., Wang H.X., Guo S.-B. (2019). Genetic ablation and pharmacological inhibition of immunosubunit β5i attenuates cardiac remodeling in deoxycorticosterone-acetate (DOCA)-salt hypertensive mice. J. Mol. Cell. Cardiol..

[27] Cappetta D., De Angelis A., Ciuffreda L.P., Coppini R., Cozzolino A., Miccichè A., Dell’Aversana C., D’Amario D., Cianflone E., Scavone C. (2020). Amelioration of diastolic dysfunction by dapagliflozin in a non-diabetic model involves coronary endothelium. Pharmacol. Res..

[28] Cezar M.D., Damatto R.L., Pagan L.U., Lima A.R., Martinez P.F., Bonomo C., Rosa C.M., Campos D.H., Cicogna A.C., Gomes M.J. (2015). Early Spironolactone Treatment Attenuates Heart Failure Development by Improving Myocardial Function and Reducing Fibrosis in Spontaneously Hypertensive Rats. Cell. Physiol. Biochem..

[29] Chan V., Hoey A., Brown L. (2006). Improved cardiovascular function with aminoguanidine in DOCA-salt hypertensive rats. Br. J. Pharmacol..

[30] Chang D., Xu T.T., Zhang S.J., Cai Y., Min S.D., Zhao Z., Lu C.Q., Wang Y.C., Ju S. (2021). Telmisartan ameliorates cardiac fibrosis and diastolic function in cardiorenal heart failure with preserved ejection fraction. Exp. Biol. Med..

[31] Chang S.A., Kim Y.J., Lee H.W., Kim D.H., Kim H.K., Chang H.J., Sohn D.W., Oh B.H., Park Y.B. (2009). Effect of rosuvastatin on cardiac remodeling. function. and progression to heart failure in hypertensive heart with established left ventricular hypertrophy. Hypertension.

[32] Chang X., Zhang T., Wang J., Liu Y., Yan P., Meng Q., Yin Y., Wang S. (2021). SIRT5-Related Desuccinylation Modification Contributes to Quercetin-Induced Protection against Heart Failure and High-Glucose-Prompted Cardiomyocytes Injured through Regulation of Mitochondrial Quality Surveillance. Oxid. Med. Cell. Longev..

[33] Chen C., Zou L.X., Lin Q.Y., Yan X., Bi H.L., Xie X., Wang S., Wang Q.S., Zhang Y.L., Li H.H. (2019). Resveratrol as a new inhibitor of immunoproteasome prevents PTEN degradation and attenuates cardiac hypertrophy after pressure overload. Redox Biol..

[34] Chen F., Wu J.L., Fu G.S., Mou Y., Hu S.J. (2015). Chronic treatment with qiliqiangxin ameliorates aortic endothelial cell dysfunction in diabetic rats. J. Cardiovasc. Pharmacol. Ther..

[35] Chen H., Zhuo C., Zu A., Yuan S., Zhang H., Zhao J., Zheng L. (2022). Thymoquinone ameliorates pressure overload-induced cardiac hypertrophy by activating the AMPK signalling pathway. J. Cell. Mol. Med..

[36] Chen X., Zhang Z., Zhang X., Jia Z., Liu J., Chen X., Xu A., Liang X., Li G. (2022). Paeonol attenuates heart failure induced by transverse aortic constriction via ERK1/2 signalling. Pharm. Biol..

[37] Chen Y., Wang L., Huang S., Ke J., Wang Q., Zhou Z., Chang W. (2021). Lutein attenuates angiotensin II-induced cardiac remodeling by inhibiting AP-1/IL-11 signaling. Redox. Biol..

[38] Chi L., Belardinelli L., Zeng A., Hirakawa R., Rajamani S., Ling H., Dhalla A.K. (2016). Inhibition of late Na+ current. a novel target to improve diastolic function and electrical abnormalities in Dahl salt-sensitive rats. Am. J. Physiol. Heart Circ. Physiol..

[39] Chinnakkannu P., Reese C., Gaspar J.A., Panneerselvam S., Pleasant-Jenkins D., Mukherjee R., Baicu C., Tourkina E., Hoffman S., Kuppuswamy D. (2018). Suppression of angiotensin II-induced pathological changes in heart and kidney by the caveolin-1 scaffolding domain peptide. PLoS ONE.

[40] Choudhary R., Palm-Leis A., Scott R.C., Guleria R.S., Rachut E., Baker K.M., Pan J. (2008). All-trans retinoic acid prevents development of cardiac remodeling in aortic banded rats by inhibiting the renin-angiotensin system. Am. J. Physiol. Heart Circ. Physiol..

[41] Davila A., Tian Y., Czikora I., Li J., Su H., Huo Y., Patel V., Robinson V., Kapuku G., Weintraub N. (2019). Adenosine Kinase Inhibition Augments Conducted Vasodilation and Prevents Left Ventricle Diastolic Dysfunction in Heart Failure With Preserved Ejection Fraction. Circ. Heart Fail..

[42] De Angelis A., Cappetta D., Piegari E., Rinaldi B., Ciuffreda L.P., Esposito G., Ferraiolo F.A., Rivellino A., Russo R., Donniacuo M. (2016). Long-term administration of ranolazine attenuates diastolic dysfunction and adverse myocardial remodeling in a model of heart failure with preserved ejection fraction. Int. J. Cardiol..

[43] Duda M.K., O’Shea K.M., Tintinu A., Xu W., Khairallah R.J., Barrows B.R., Chess D.J., Azimzadeh A.M., Harris W.S., Sharov V.G. (2009). Fish oil. but not flaxseed oil. decreases inflammation and prevents pressure overload-induced cardiac dysfunction. Cardiovasc. Res..

[44] Dulce R.A., Kanashiro-Takeuchi R.M., Takeuchi L.M., Salerno A.G., Wanschel A.C.B.A., Kulandavelu S., Balkan W., Zuttion M.S.S.R., Cai R., Schally A.V. (2022). Synthetic growth hormone-releasing hormone agonist ameliorates the myocardial pathophysiology characteristic of HFpEF. Cardiovasc. Res..

[45] Esposito G., Cappetta D., Russo R., Rivellino A., Ciuffreda L.P., Roviezzo F., Piegari E., Berrino L., Rossi F., De Angelis A. (2017). Sitagliptin reduces inflammation. fibrosis and preserves diastolic function in a rat model of heart failure with preserved ejection fraction. Br. J. Pharmacol..

[46] Evaristi M.F., Poirier B., Chénedé X., Lefebvre A.M., Roccon A., Gillot F., Beeské S., Corbier A., Pruniaux-Harnist M.P., Janiak P. (2022). A G-protein-biased S1P1 agonist. SAR247799. improved LVH and diastolic function in a rat model of metabolic syndrome. PLoS ONE.

[47] Fenning A., Harrison G., Rose’meyer R., Hoey A., Brown L. (2005). l-Arginine attenuates cardiovascular impairment in DOCA-salt hypertensive rats. Am. J. Physiol. Heart Circ. Physiol..

[48] Ge Q., Zhao L., Liu C., Ren X., Yu Y.H., Pan C., Hu Z. (2020). LCZ696. an Angiotensin Receptor-Neprilysin inhibitor. Improves Cardiac Hypertrophy and Fibrosis and Cardiac Lymphatic Remodeling in Transverse Aortic Constriction Model Mice. BioMed Res. Int..

[49] Gimenes R., Gimenes C., Rosa C.M., Xavier N.P., Campos D.H.S., Fernandes A.A.H., Cezar M.D.M., Guirado G.N., Pagan L.U., Chaer I.D. (2018). Influence of apocynin on cardiac remodeling in rats with streptozotocin-induced diabetes mellitus. Cardiovasc. Diabetol..

[50] Giri S.R., Bhoi B., Jain M.R., Gatne M.M. (2016). Cardioprotective role of peroxisome proliferator-activated receptor-γ agonist. rosiglitazone in a unique murine model of diabetic cardiopathy. Life Sci..

[51] Gladden J.D., Zelickson B.R., Guichard J.L., Ahmed M.I., Yancey D.M., Ballinger S., Shanmugam M., Babu G.J., Johnson M.S., Darley-Usmar V. (2013). Xanthine oxidase inhibition preserves left ventricular systolic but not diastolic function in cardiac volume overload. Am. J. Physiol. Heart Circ. Physiol..

[52] Goltsman I., Khoury E.E., Aronson D., Nativ O., Feuerstein G.Z., Winaver J., Abassi Z. (2019). Rosiglitazone treatment restores renal responsiveness to atrial natriuretic peptide in rats with congestive heart failure. J. Cell. Mol. Med..

[53] Gómez-Garre D., González-Rubio M.L., Muñoz-Pacheco P., Caro-Vadillo A., Aragoncillo P., Fernández-Cruz A. (2010). Rosuvastatin added to standard heart failure therapy improves cardiac remodelling in heart failure rats with preserved ejection fraction. Eur. J. Heart Fail..

[54] Gómez-Hurtado N., Domínguez-Rodríguez A., Mateo P., Fernández-Velasco M., Val-Blasco A., Aizpún R., Sabourin J., Gómez A.M., Benitah J.P., Delgado C. (2017). Beneficial effects of leptin treatment in a setting of cardiac dysfunction induced by transverse aortic constriction in mouse. J. Physiol..

[55] Gong W., Yan M., Chen J., Chaugai S., Chen C., Wang D. (2014). Chronic inhibition of cyclic guanosine monophosphate-specific phosphodiesterase 5 prevented cardiac fibrosis through inhibition of transforming growth factor β-induced Smad signaling. Front. Med..

[56] Gong W., Duan Q., Cai Z., Chen C., Ni L., Yan M., Wang X., Cianflone K., Wang D.W. (2013). Chronic inhibition of cGMP-specific phosphodiesterase 5 suppresses endoplasmic reticulum stress in heart failure. Br. J. Pharmacol..

[57] Gonzalez L., Novoa U., Moya J., Gabrielli L., Jalil J.E., García L., Chiong M., Lavandero S., Ocaranza M.P. (2018). Angiotensin-(1-9) reduces cardiovascular and renal inflammation in experimental renin-independent hypertension. Biochem. Pharmacol..

[58] Grune J., Benz V., Brix S., Salatzki J., Blumrich A., Höft B., Klopfleisch R., Foryst-Ludwig A., Kolkhof P., Kintscher U. (2016). Steroidal and Nonsteroidal Mineralocorticoid Receptor Antagonists Cause Differential Cardiac Gene Expression in Pressure Overload-induced Cardiac Hypertrophy. J. Cardiovasc. Pharmacol..

[59] Guan X., Guan X., Lu C., Shang B., Zhao Y., Meng Y., Zhang Z. (2020). Nebivolol combined with tetrahydrobiopterin affects diastolic function in spontaneously hypertensive rats via the nitric oxide/cyclic guanosine monophosphate signalling pathway. BMC Pharmacol. Toxicol..

[60] Habibi J., Aroor A.R., Sowers J.R., Jia G., Hayden M.R., Garro M., Barron B., Mayoux E., Rector R.S., Whaley-Connell A. (2017). Sodium glucose transporter 2 (SGLT2) inhibition with empagliflozin improves cardiac diastolic function in a female rodent model of diabetes. Cardiovasc. Diabetol..

[61] Hamdani N., Hervent A.S., Vandekerckhove L., Matheeussen V., Demolder M., Baerts L., De Meester I., Linke W.A., Paulus W.J., De Keulenaer G.W. (2014). Left ventricular diastolic dysfunction and myocardial stiffness in diabetic mice is attenuated by inhibition of dipeptidyl peptidase 4. Cardiovasc. Res..

[62] Hammoudi N., Jeong D., Singh R., Farhat A., Komajda M., Mayoux E., Hajjar R., Lebeche D. (2017). Empagliflozin Improves Left Ventricular Diastolic Dysfunction in a Genetic Model of Type 2 Diabetes. Cardiovasc. Drugs Ther..

[63] Han X., Peng C., Huang L., Luo X., Mao Q., Wu S., Zhang H. (2022). EGCG prevents pressure overload-induced myocardial remodeling by downregulating overexpression of HDAC5 in mice. Int. J. Mol. Med..

[64] Henderson B.C., Sen U., Reynolds C., Moshal K.S., Ovechkin A., Tyagi N., Kartha G.K., Rodriguez W.E., Tyagi S.C. (2007). Reversal of systemic hypertension-associated cardiac remodeling in chronic pressure overload myocardium by ciglitazone. Int. J. Biol. Sci..

[65] Horvath O., Ordog K., Bruszt K., Deres L., Gallyas F., Sumegi B., Toth K., Halmosi R. (2021). BGP-15 Protects against Heart Failure by Enhanced Mitochondrial Biogenesis and Decreased Fibrotic Remodelling in Spontaneously Hypertensive Rats. Oxid. Med. Cell. Longev..

[66] Hou N., Mai Y., Qiu X., Yuan W., Li Y., Luo C., Liu Y., Zhang G., Zhao G., Luo J.D. (2019). Carvacrol Attenuates Diabetic Cardiomyopathy by Modulating the PI3K/AKT/GLUT4 Pathway in Diabetic Mice. Front. Pharmacol..

[67] Huang J.P., Huang S.S., Deng J.Y., Chang C.C., Day Y.J., Hung L.M. (2010). Insulin and resveratrol act synergistically preventing cardiac dysfunction in diabetes, but the advantage of resveratrol in diabetics with acute heart attack is antagonized by insulin. Free Radic. Biol. Med..

[68] Huang S., Wang W., Li L., Wang T., Zhao Y., Lin Y., Huang W., Wang Y., Huang Z. (2021). P2X7 Receptor Deficiency Ameliorates STZ-induced Cardiac Damage and Remodeling Through PKCβ and ERK. Front. Cell. Dev. Biol..

[69] Huang Y., Zhang K., Jiang M., Ni J., Chen J., Li L., Deng J., Zhu Y., Mao J., Gao X. (2020). Regulation of energy metabolism by combination therapy attenuates cardiac metabolic remodeling in heart failure. Int. J. Biol. Sci..

[70] Huang Y., Zhang K., Liu M., Su J., Qin X., Wang X., Zhang J., Li S., Fan G. (2021). An herbal preparation ameliorates heart failure with preserved ejection fraction by alleviating microvascular endothelial inflammation and activating NO-cGMP-PKG pathway. Phytomedicine.

[71] Huc T., Drapala A., Gawrys M., Konop M., Bielinska K., Zaorska E., Samborowska E., Wyczalkowska-Tomasik A., Pączek L., Dadlez M. (2018). Chronic, low-dose TMAO treatment reduces diastolic dysfunction and heart fibrosis in hypertensive rats. Am. J. Physiol. Heart Circ. Physiol..

[72] Huo S., Shi W., Ma H., Yan D., Luo P., Guo J., Li C., Lin J., Zhang C., Li S. (2021). Alleviation of Inflammation and Oxidative Stress in Pressure Overload-Induced Cardiac Remodeling and Heart Failure via IL-6/STAT3 Inhibition by Raloxifene. Oxid. Med. Cell. Longev..

[73] Ikeda J., Kimoto N., Kitayama T., Kunori S. (2016). Cardiac DPP-4 inhibition by saxagliptin ameliorates isoproterenol-induced myocardial remodeling and cardiac diastolic dysfunction in rats. J. Pharmacol. Sci..

[74] Jackson M.R., Cox K.D., Baugh S.D.P., Wakeen L., Rashad A.A., Lam P.Y.S., Polyak B., Jorns M.S. (2022). Discovery of a first-in-class inhibitor of sulfide:quinone oxidoreductase that protects against adverse cardiac remodelling and heart failure. Cardiovasc. Res..

[75] Jeong E.M., Monasky M.M., Gu L., Taglieri D.M., Patel B.G., Liu H., Wang Q., Greener I., Dudley S.C., Solaro R.J. (2013). Tetrahydrobiopterin improves diastolic dysfunction by reversing changes in myofilament properties. J. Mol. Cell. Cardiol..

[76] Jeong M.Y., Lin Y.H., Wennersten S.A., Demos-Davies K.M., Cavasin M.A., Mahaffey J.H., Monzani V., Saripalli C., Mascagni P., Reece T.B. (2018). Histone deacetylase activity governs diastolic dysfunction through a nongenomic mechanism. Sci. Transl. Med..

[77] Jiang X., Yang F., Ou D., Huang L., Li H., Lang M. (2022). MCC950 ameliorates ventricular arrhythmia vulnerability induced by heart failure. Bioengineered..

[78] Johnson J.A., West J., Maynard K.B., Hemnes A.R. (2011). ACE2 improves right ventricular function in a pressure overload model. PLoS ONE.

[79] Joubert M., Jagu B., Montaigne D., Marechal X., Tesse A., Ayer A., Dollet L., Le May C., Toumaniantz G., Manrique A. (2017). The Sodium-Glucose Cotransporter 2 inhibitor Dapagliflozin Prevents Cardiomyopathy in a Diabetic Lipodystrophic Mouse Model. Diabetes.

[80] Juric D., Wojciechowski P., Das D.K., Netticadan T. (2007). Prevention of concentric hypertrophy and diastolic impairment in aortic-banded rats treated with resveratrol. Am. J. Physiol. Heart Circ. Physiol..

[81] Kakehi K., Iwanaga Y., Watanabe H., Sonobe T., Akiyama T., Shimizu S., Yamamoto H., Miyazaki S. (2019). Modulation of Sympathetic Activity and Innervation With Chronic Ivabradine and β-Blocker Therapies: Analysis of Hypertensive Rats with Heart Failure. J. Cardiovasc. Pharmacol. Ther..

[82] Kamiya M., Asai K., Maejima Y., Shirakabe A., Murai K., Noma S., Komiyama H., Sato N., Mizuno K., Shimizu W. (2021). β3-Adrenergic Receptor Agonist Prevents Diastolic Dysfunction in an Angiotensin II-Induced Cardiomyopathy Mouse Model. J. Pharmacol. Exp. Ther..

[83] Katare R.G., Caporali A., Oikawa A., Meloni M., Emanueli C., Madeddu P. (2010). Vitamin B1 analog benfotiamine prevents diabetes-induced diastolic dysfunction and heart failure through Akt/Pim-1-mediated survival pathway. Circ. Heart Fail..

[84] Khong F.L., Zhang Y., Edgley A.J., Qi W., Connelly K.A., Woodman O.L., Krum H., Kelly D.J. (2011). 3′,4′-Dihydroxyflavonol antioxidant attenuates diastolic dysfunction and cardiac remodeling in streptozotocin-induced diabetic m(Ren2)27 rats. PLoS ONE.

[85] Kim S., Yoshiyama M., Izumi Y., Kawano H., Kimoto M., Zhan Y., Iwao H. (2001). Effects of combination of ACE inhibitor and angiotensin receptor blocker on cardiac remodeling, cardiac function, and survival in rat heart failure. Circulation.

[86] Kim-Mitsuyama S., Izumi Y., Izumiya Y., Yoshida K., Yoshiyama M., Iwao H. (2004). Additive beneficial effects of the combination of a calcium channel blocker and an angiotensin blocker on a hypertensive rat-heart failure model. Hypertens. Res..

[87] Lapinskas T., Kelle S., Grune J., Foryst-Ludwig A., Meyborg H., Jeuthe S., Wellnhofer E., Elsanhoury A., Pieske B., Gebker R. (2020). Serelaxin Improves Regional Myocardial Function in Experimental Heart Failure: An In Vivo Cardiac Magnetic Resonance Study. J. Am. Heart. Assoc..

[88] Lee H.C., Shiou Y.L., Jhuo S.J., Chang C.Y., Liu P.L., Jhuang W.J., Dai Z.K., Chen W.Y., Chen Y.F., Lee A.S. (2019). The sodium-glucose co-transporter 2 inhibitor empagliflozin attenuates cardiac fibrosis and improves ventricular hemodynamics in hypertensive heart failure rats. Cardiovasc. Diabetol..

[89] Leite S., Moreira-Costa L., Cerqueira R., Sousa-Mendes C., Angélico-Gonçalves A., Fontoura D., Vasques-Nóvoa F., Leite-Moreira A.F., Lourenço A.P. (2021). Chronic Sildenafil Therapy in the ZSF1 Obese Rat Model of Metabolic Syndrome and Heart Failure With Preserved Ejection Fraction. J. Cardiovasc. Pharmacol. Ther..

[90] Li L., Luo W., Qian Y., Zhu W., Qian J., Li J., Jin Y., Xu X., Liang G. (2019). Luteolin protects against diabetic cardiomyopathy by inhibiting NF-κB-mediated inflammation and activating the Nrf2-mediated antioxidant responses. Phytomedicine.

[91] Li X., Lu Q., Qiu Y., do Carmo J.M., Wang Z., da Silva A.A., Mouton A., Omoto A.C.M., Hall M.E., Li J. (2021). Direct Cardiac Actions of the Sodium Glucose Co-Transporter 2 inhibitor Empagliflozin Improve Myocardial Oxidative Phosphorylation and Attenuate Pressure-Overload Heart Failure. J. Am. Heart Assoc..

[92] Li Z., Wang Y., Jiang Y., Ma D., Jiang P., Zhou G., Yang J., Dong F., Zhao H., Zhang Y. (2020). Xiao-Qing-Long-Tang Maintains Cardiac Function during Heart Failure with Reduced Ejection Fraction in Salt-Sensitive Rats by Regulating the Imbalance of Cardiac Sympathetic Innervation. Evid.-Based Complement. Alternat. Med..

[93] Liao H.H., Zhang N., Feng H., Zhang N., Ma Z.G., Yang Z., Yuan Y., Bian Z.Y., Tang Q.Z. (2015). Oleanolic acid alleviated pressure overload-induced cardiac remodeling. Mol. Cell. Biochem..

[94] Liao Y., Zhao H., Ogai A., Kato H., Asakura M., Kim J., Asanuma H., Minamino T., Takashima S., Kitakaze M. (2008). Atorvastatin slows the progression of cardiac remodeling in mice with pressure overload and inhibits epidermal growth factor receptor activation. Hypertens. Res..

[95] Liu L., Wang W., Meng X., Gao J., Wu H., Wang P., Wu W., Wang L., Ma L., Zhang W. (2010). Left ventricular hypertrophy induced by abdominal aortic banding and its prevention by angiotensin receptor blocker telmisartan--a proteomic analysis. J. Physiol. Biochem..

[96] Liu L., Zhao W., Liu J., Gan Y., Liu L., Tian J. (2018). Epigallocatechin-3 gallate prevents pressure overload-induced heart failure by up-regulating SERCA2a via histone acetylation modification in mice. PLoS ONE.

[97] Liu W., Zi M., Tsui H., Chowdhury S.K., Zeef L., Meng Q.J., Travis M., Prehar S., Berry A., Hanley N.A. (2013). A novel immunomodulatory, FTY-720 reverses existing cardiac hypertrophy and fibrosis from pressure overload by targeting NFAT (nuclear factor of activated T-cells) signaling and periostin. Circ. Heart Fail..

[98] Liu X.Y., Liao H.H., Feng H., Zhang N., Yang J.J., Li W.J., Chen S., Deng W., Tang Q.Z. (2018). Icariside II attenuates cardiac remodeling via AMPKα2/mTORC1 in vivo and in vitro. J. Pharmacol. Sci..

[99] Liu Y., Li S., Zhang Z., Lv Z., Jiang H., Tan X., Liu F. (2017). Effects of valproic acid on sympathetic activity and left ventricularmyocardial remodelling in rats during pressure overload. Turk. J. Med. Sci..

[100] Loch D., Chan V., Hoey A., Brown L. (2009). Rosuvastatin attenuates heart failure and cardiac remodelling in the ageing spontaneously hypertensive rat. Basic Clin. Pharmacol. Toxicol..

[101] Lou T., Ma J., Xie Y., Yao G., Fan Y., Ma S., Zou X. (2021). Nuanxin capsule enhances cardiac function by inhibiting oxidative stress-induced mitochondrial dependent apoptosis through AMPK/JNK signaling pathway. Biomed. Pharmacother..

[102] Louhelainen M., Merasto S., Finckenberg P., Vahtola E., Kaheinen P., Leskinen H., Levijoki J., Pollesello P., Haikala H., Mervaala E.M. (2009). Effects of calcium sensitizer OR-1986 on a cardiovascular mortality and myocardial remodelling in hypertensive Dahl/Rapp rats. J. Physiol. Pharmacol..

[103] Louhelainen M., Vahtola E., Kaheinen P., Leskinen H., Merasto S., Kytö V., Finckenberg P., Colucci W.S., Levijoki J., Pollesello P. (2007). Effects of levosimendan on cardiac remodeling and cardiomyocyte apoptosis in hypertensive Dahl/Rapp rats. Br. J. Pharmacol..

[104] Lovelock J.D., Monasky M.M., Jeong E.M., Lardin H.A., Liu H., Patel B.G., Taglieri D.M., Gu L., Kumar P., Pokhrel N. (2012). Ranolazine improves cardiac diastolic dysfunction through modulation of myofilament calcium sensitivity. Circ. Res..

[105] Lu J., Pontré B., Pickup S., Choong S.Y., Li M., Xu H., Gamble G.D., Phillips A.R., Cowan B.R., Young A.A. (2013). Treatment with a copper-selective chelator causes substantive improvement in cardiac function of diabetic rats with left-ventricular impairment. Cardiovasc. Diabetol..

[106] Luk F.S., Kim R.Y., Li K., Ching D., Wong D.K., Joshi S.K., Imhof I., Honbo N., Hoover H., Zhu B.Q. (2016). Immunosuppression With FTY720 Reverses Cardiac Dysfunction in Hypomorphic ApoE Mice Deficient in SR-BI Expression That Survive Myocardial Infarction Caused by Coronary Atherosclerosis. J. Cardiovasc. Pharmacol..

[107] Ma S., Feng J., Lin X., Liu J., Tang Y., Nie S., Gong J., Wang L. (2021). Nicotinamide Riboside Alleviates Cardiac Dysfunction and Remodeling in Pressure Overload Cardiac Hypertrophy. Oxid. Med. Cell. Longev..

[108] Ma X.L., Lin Q.Y., Wang L., Xie X., Zhang Y.L., Li H.H. (2019). Rituximab prevents and reverses cardiac remodeling by depressing B cell function in mice. Biomed. Pharmacother..

[109] Ma Y., Huang H., Jiang J., Wu L., Lin C., Tang A., Dai G., He J., Chen Y. (2016). AVE 0991 attenuates cardiac hypertrophy through reducing oxidative stress. Biochem. Biophys. Res. Commun..

[110] Madonna R., Doria V., Minnucci I., Pucci A., Pierdomenico D.S., De Caterina R. (2020). Empagliflozin reduces the senescence of cardiac stromal cells and improves cardiac function in a murine model of diabetes. J. Cell. Mol. Med..

[111] Mátyás C., Németh B.T., Oláh A., Török M., Ruppert M., Kellermayer D., Barta B.A., Szabó G., Kökény G., Horváth E.M. (2017). Prevention of the development of heart failure with preserved ejection fraction by the phosphodiesterase-5A inhibitor vardenafil in rats with type 2 diabetes. Eur. J. Heart Fail..

[112] Methatham T., Tomida S., Kimura N., Imai Y., Aizawa K. (2021). Inhibition of the canonical Wnt signaling pathway by a β-catenin/CBP inhibitor prevents heart failure by ameliorating cardiac hypertrophy and fibrosis. Sci. Rep..

[113] Methawasin M., Strom J., Borkowski T., Hourani Z., Runyan R., Smith J.E., Granzier H. (2020). Phosphodiesterase 9a Inhibition in Mouse Models of Diastolic Dysfunction. Circ. Heart Fail..

[114] Minhas K.M., Saraiva R.M., Schuleri K.H., Lehrke S., Zheng M., Saliaris A.P., Berry C.E., Barouch L.A., Vandegaer K.M., Li D. (2006). Xanthine oxidoreductase inhibition causes reverse remodeling in rats with dilated cardiomyopathy. Circ. Res..

[115] Mishra M., Muthuramu I., Aboumsallem J.P., Kempen H., De Geest B. (2018). Reconstituted HDL (Milano) Treatment Efficaciously Reverses Heart Failure with Preserved Ejection Fraction in Mice. Int. J. Mol. Sci..

[116] Mishra M., Muthuramu I., Kempen H., De Geest B. (2020). Administration of apo A-I (Milano) nanoparticles reverses pathological remodelling cardiac dysfunction and heart failure in a murine model of HFpEF associated with hypertension. Sci. Rep..

[117] Morgan L.A., Olzinski A.R., Upson J.J., Zhao S., Wang T., Eisennagel S.H., Hoang B., Tunstead J.R., Marino J.P., Willette R.N. (2013). Soluble epoxide hydrolase inhibition does not prevent cardiac remodeling and dysfunction after aortic constriction in rats and mice. J. Cardiovasc. Pharmacol..

[118] Muñoz-Pacheco P., Ortega-Hernández A., Caro-Vadillo A., Casanueva-Eliceiry S., Aragoncillo P., Egido J., Fernández-Cruz A., Gómez-Garre D. (2013). Eplerenone enhances cardioprotective effects of standard heart failure therapy through matricellular proteins in hypertensive heart failure. J. Hypertens..

[119] Naruse G., Kanamori H., Yoshida A., Minatoguchi S., Kawaguchi T., Iwasa M., Yamada Y., Mikami A., Kawasaki M., Nishigaki K. (2019). The intestine responds to heart failure by enhanced mitochondrial fusion through glucagon-like peptide-1 signalling. Cardiovasc. Res..

[120] Nguyen T.D., Shingu Y., Amorim P.A., Schenkl C., Schwarzer M., Doenst T. (2018). GLP-1 Improves Diastolic Function and Survival in Heart Failure with Preserved Ejection Fraction. J. Cardiovasc. Transl. Res..

[121] Nie J., Duan Q., He M., Li X., Wang B., Zhou C., Wu L., Wen Z., Chen C., Wang D.W. (2019). Ranolazine prevents pressure overload-induced cardiac hypertrophy and heart failure by restoring aberrant Na^+^ and Ca^2+^ handling. J. Cell. Physiol..

[122] Nordén E.S., Bendiksen B.A., Andresen H., Bergo K.K., Espe E.K., Hasic A., Hauge-Iversen I.M., Veras I., Hussain R.I., Sjaastad I. (2021). Sacubitril/valsartan ameliorates cardiac hypertrophy and preserves diastolic function in cardiac pressure overload. ESC Heart Fail..

[123] Ohtani T., Ohta M., Yamamoto K., Mano T., Sakata Y., Nishio M., Takeda Y., Yoshida J., Miwa T., Okamoto M. (2007). Elevated cardiac tissue level of aldosterone and mineralocorticoid receptor in diastolic heart failure: Beneficial effects of mineralocorticoid receptor blocker. Am. J. Physiol. Regul. Integr. Comp. Physiol..

[124] Oishi S., Suzuki N., Hasui Y., Homma T., Obana M., Nagayama T., Fujio Y. (2017). Sustained Activation of Guanylate Cyclase-A with TDT a Natriuretic Peptide Derivative Exhibits Cardiorenal Protection in Dahl Salt-Sensitive Hypertensive Rats. J. Pharmacol. Exp. Ther..

[125] O’Shea K.M., Chess D.J., Khairallah R.J., Hecker P.A., Lei B., Walsh K., Des Rosiers C., Stanley W.C. (2010). ω-3 Polyunsaturated fatty acids prevent pressure overload-induced ventricular dilation and decrease in mitochondrial enzymes despite no change in adiponectin. Lipids Health Dis..

[126] Park S.H., Farooq M.A., Gaertner S., Bruckert C., Qureshi A.W., Lee H.H., Benrahla D., Pollet B., Stephan D., Ohlmann P. (2020). Empagliflozin improved systolic blood pressure, endothelial dysfunction and heart remodeling in the metabolic syndrome ZSF1 rat. Cardiovasc. Diabetol..

[127] Perlini S., Ferrero I., Palladini G., Tozzi R., Gatti C., Vezzoli M., Cesana F., Janetti M.B., Clari F., Busca G. (2006). Survival benefits of different antiadrenergic interventions in pressure overload left ventricular hypertrophy/failure. Hypertension.

[128] Plante E., Menaouar A., Danalache B.A., Broderick T.L., Jankowski M., Gutkowska J. (2014). Treatment with brain natriuretic peptide prevents the development of cardiac dysfunction in obese diabetic db/db mice. Diabetologia.

[129] Pozder Geb Gehlken C., Rogier van der Velde A., Meijers W.C., Silljé H.H.W., Muntendam P., Dokter M.M., van Gilst W.H., Schols H.A., de Boer R.A. (2022). Pectins from various sources inhibit galectin-3-related cardiac fibrosis. Curr. Res. Transl. Med..

[130] Primessnig U., Bracic T., Levijoki J., Otsomaa L., Pollesello P., Falcke M., Pieske B., Heinzel F.R. (2019). Long-term effects of Na^+^/Ca^2+^ exchanger inhibition with ORM-11035 improves cardiac function and remodelling without lowering blood pressure in a model of heart failure with preserved ejection fraction. Eur. J. Heart Fail..

[131] Qiu Z., Zhang W., Fan F., Li H., Wu C., Ye Y., Du Q., Li Z., Hu X., Zhao G. (2012). Rosuvastatin-attenuated heart failure in aged spontaneously hypertensive rats via PKCα/β2 signal pathway. J. Cell. Mol. Med..

[132] Randriamboavonjy J.I., Loirand G., Vaillant N., Lauzier B., Derbré S., Michalet S., Pacaud P., Tesse A. (2016). Cardiac Protective Effects of Moringa oleifera Seeds in Spontaneous Hypertensive Rats. Am. J. Hypertens..

[133] Reddy S.S., Agarwal H., Barthwal M.K. (2018). Cilostazol ameliorates heart failure with preserved ejection fraction and diastolic dysfunction in obese and non-obese hypertensive mice. J. Mol. Cell. Cardiol..

[134] Richards D.A., Aronovitz M.J., Liu P., Martin G.L., Tam K., Pande S., Karas R.H., Bloomfield D.M., Mendelsohn M.E., Blanton R.M. (2021). CRD-733, a Novel PDE9 (Phosphodiesterase 9) inhibitor, Reverses Pressure Overload-Induced Heart Failure. Circ. Heart Fail..

[135] Rimbaud S., Ruiz M., Piquereau J., Mateo P., Fortin D., Veksler V., Garnier A., Ventura-Clapier R. (2011). Resveratrol improves survival, hemodynamics and energetics in a rat model of hypertension leading to heart failure. PLoS ONE.

[136] Russell-Hallinan A., Neary R., Watson C.J., Baugh J.A. (2021). Repurposing From Oncology to Cardiology: Low-Dose 5-Azacytidine Attenuates Pathological Cardiac Remodeling in Response to Pressure Overload Injury. J. Cardiovasc. Pharmacol. Ther..

[137] Salah E.M., Bastacky S.I., Jackson E.K., Tofovic S.P. (2018). Captopril Attenuates Cardiovascular and Renal Disease in a Rat Model of Heart Failure With Preserved Ejection Fraction. J. Cardiovasc. Pharmacol..

[138] Satoh S., Ueda Y., Koyanagi M., Kadokami T., Sugano M., Yoshikawa Y., Makino N. (2003). Chronic inhibition of Rho kinase blunts the process of left ventricular hypertrophy leading to cardiac contractile dysfunction in hypertension-induced heart failure. J. Mol. Cell. Cardiol..

[139] Satoh S., Ueda Y., Suematsu N., Oyama J., Kadokami T., Sugano M., Yoshikawa Y., Makino N. (2003). Beneficial effects of angiotensin-converting enzyme inhibition on sarcoplasmic reticulum function in the failing heart of the Dahl rat. Circ. J..

[140] Schauer A., Adams V., Augstein A., Jannasch A., Draskowski R., Kirchhoff V., Goto K., Mittag J., Galli R., Männel A. (2021). Sacubitril/Valsartan Improves Diastolic Function But Not Skeletal Muscle Function in a Rat Model of HFpEF. Int. J. Mol. Sci..

[141] Seymour E.M., Singer A.A., Bennink M.R., Parikh R.V., Kirakosyan A., Kaufman P.B., Bolling S.F. (2008). Chronic intake of a phytochemical-enriched diet reduces cardiac fibrosis and diastolic dysfunction caused by prolonged salt-sensitive hypertension. J. Gerontol. A Biol. Sci. Med. Sci..

[142] Shang L., Weng X., Wang D., Yue W., Mernaugh R., Amarnath V., Weir E.K., Dudley S.C., Xu Y., Hou M. (2019). Isolevuglandin scavenger attenuates pressure overload-induced cardiac oxidative stress, cardiac hypertrophy, heart failure and lung remodeling. Free Radic. Biol. Med..

[143] Shao S., Zhang Y., Gong M., Yang Q., Yuan M., Yuan M., Suo Y., Wang X., Li Y., Bao Q. (2021). Ivabradine Ameliorates Cardiac Function in Heart Failure with Preserved and Reduced Ejection Fraction via Upregulation of miR-133a. Oxid. Med. Cell. Longev..

[144] Shea C.M., Price G.M., Liu G., Sarno R., Buys E.S., Currie M.G., Masferrer J.L. (2020). Soluble guanylate cyclase stimulator praliciguat attenuates inflammation, fibrosis, and end-organ damage in the Dahl model of cardiorenal failure. Am. J. Physiol.-Renal Physiol..

[145] Shiraki A., Oyama J.I., Nishikido T., Node K. (2019). GLP-1 analog liraglutide-induced cardiac dysfunction due to energetic starvation in heart failure with non-diabetic dilated cardiomyopathy. Cardiovasc. Diabetol..

[146] Signore P.E., Guo G., Wei Z., Zhang W., Lin A., Del Balzo U. (2021). A small-molecule inhibitor of hypoxia-inducible factor prolyl hydroxylase improves obesity, nephropathy and cardiomyopathy in obese ZSF1 rats. PLoS ONE.

[147] Stolina M., Luo X., Dwyer D., Han C.Y., Chen R., Zhang Y., Xiong Y., Chen Y., Yin J., Shkumatov A. (2020). The evolving systemic biomarker milieu in obese ZSF1 rat model of human cardiometabolic syndrome: Characterization of the model and cardioprotective effect of GDF15. PLoS ONE.

[148] Sukumaran V., Tsuchimochi H., Sonobe T., Waddingham M.T., Shirai M., Pearson J.T. (2020). Liraglutide treatment improves the coronary microcirculation in insulin resistant Zucker obese rats on a high salt diet. Cardiovasc. Diabetol..

[149] Sung Y.L., Lin T.T., Syu J.Y., Hsu H.J., Lin K.Y., Liu Y.B., Lin S.F. (2020). Reverse electromechanical modelling of diastolic dysfunction in spontaneous hypertensive rat after sacubitril/valsartan therapy. ESC Heart Fail..

[150] Tamayo M., Martín-Nunes L., Val-Blasco A., G.M-Piedras M.J., Navarro-García J.A., Lage E., Prieto P., Ruiz-Hurtado G., Fernández-Velasco M., Delgado C. (2020). Beneficial effects of paricalcitol on cardiac dysfunction and remodelling in a model of established heart failure. Br. J. Pharmacol..

[151] Tang X., Chen X.F., Wang N.Y., Wang X.M., Liang S.T., Zheng W., Lu Y.B., Zhao X., Hao D.L., Zhang Z.Q. (2017). SIRT2 Acts as a Cardioprotective Deacetylase in Pathological Cardiac Hypertrophy. Circulation.

[152] Thandapilly S.J., Wojciechowski P., Behbahani J., Louis X.L., Yu L., Juric D., Kopilas M.A., Anderson H.D., Netticadan T. (2010). Resveratrol prevents the development of pathological cardiac hypertrophy and contractile dysfunction in the SHR without lowering blood pressure. Am. J. Hypertens..

[153] Thomas T.A., Kuzman J.A., Anderson B.E., Andersen S.M., Schlenker E.H., Holder M.S., Gerdes A.M. (2005). Thyroid hormones induce unique and potentially beneficial changes in cardiac myocyte shape in hypertensive rats near heart failure. Am. J. Physiol. Heart Circ. Physiol..

[154] Tian J., Zhang M., Suo M., Liu D., Wang X., Liu M., Pan J., Jin T., An F. (2021). Dapagliflozin alleviates cardiac fibrosis through suppressing EndMT and fibroblast activation via AMPKα/TGF-β/Smad signalling in type 2 diabetic rats. J. Cell. Mol. Med..

[155] Travers J.G., Wennersten S.A., Peña B., Bagchi R.A., Smith H.E., Hirsch R.A., Vanderlinden L.A., Lin Y.H., Dobrinskikh E., Demos-Davies K.M. (2021). HDAC Inhibition Reverses Preexisting Diastolic Dysfunction and Blocks Covert Extracellular Matrix Remodeling. Circulation.

[156] Valero-Munoz M., Li S., Wilson R.M., Boldbaatar B., Iglarz M., Sam F. (2016). Dual Endothelin-A/Endothelin-B Receptor Blockade and Cardiac Remodeling in Heart Failure With Preserved Ejection Fraction. Circ. Heart Fail..

[157] Venardos K., De Jong K.A., Elkamie M., Connor T., McGee S.L. (2015). The PKD inhibitor CID755673 enhances cardiac function in diabetic db/db mice. PLoS ONE.

[158] Verma S., Rawat S., Ho K.L., Wagg C.S., Zhang L., Teoh H., Dyck J.E., Uddin G.M., Oudit G.Y., Mayoux E. (2018). Empagliflozin Increases Cardiac Energy Production in Diabetes: Novel Translational Insights Into the Heart Failure Benefits of SGLT2 inhibitors. JACC Basic Transl. Sci..

[159] Wang D., Luo Y., Myakala K., Orlicky D.J., Dobrinskikh E., Wang X., Levi M. (2017). Serelaxin improves cardiac and renal function in DOCA-salt hypertensive rats. Sci. Rep..

[160] Wang J., Li Z., Wang Y., Zhang J., Zhao W., Fu M., Han X., Zhou J., Ge J. (2017). *Qiliqiangxin* Enhances Cardiac Glucose Metabolism and Improves Diastolic Function in Spontaneously Hypertensive Rats. Evid.-Based Complement. Alternat. Med..

[161] Wang L., Halliday G., Huot J.R., Satoh T., Baust J.J., Fisher A., Cook T., Hu J., Avolio T., Goncharov D.A. (2020). Treatment With Treprostinil and Metformin Normalizes Hyperglycemia and Improves Cardiac Function in Pulmonary Hypertension Associated With Heart Failure With Preserved Ejection Fraction. Arterioscler. Thromb. Vasc. Biol..

[162] Wei H., Qu H., Wang H., Ji B., Ding Y., Liu D., Duan Y., Liang H., Peng C., Xiao X. (2017). 1,25-Dihydroxyvitamin-D3 prevents the development of diabetic cardiomyopathy in type 1 diabetic rats by enhancing autophagy via inhibiting the β-catenin/TCF4/GSK-3β/mTOR pathway. J. Steroid Biochem. Mol. Biol..

[163] Westermann D., Becher P.M., Lindner D., Savvatis K., Xia Y., Fröhlich M., Hoffmann S., Schultheiss H.P., Tschöpe C. (2012). Selective PDE5A inhibition with sildenafil rescues left ventricular dysfunction, inflammatory immune response and cardiac remodeling in angiotensin II-induced heart failure in vivo. Basic Res. Cardiol..

[164] Westermann D., Riad A., Richter U., Jäger S., Savvatis K., Schuchardt M., Bergmann N., Tölle M., Nagorsen D., Gotthardt M. (2009). Enhancement of the endothelial NO synthase attenuates experimental diastolic heart failure. Basic Res. Cardiol..

[165] Westermann D., Rutschow S., Jäger S., Linderer A., Anker S., Riad A., Unger T., Schultheiss H.P., Pauschinger M., Tschöpe C. (2007). Contributions of inflammation and cardiac matrix metalloproteinase activity to cardiac failure in diabetic cardiomyopathy: The role of angiotensin type 1 receptor antagonism. Diabetes.

[166] Wilck N., Markó L., Balogh A., Kräker K., Herse F., Bartolomaeus H., Szijártó I.A., Gollasch M., Reichhart N., Strauss O. (2018). Nitric oxide-sensitive guanylyl cyclase stimulation improves experimental heart failure with preserved ejection fraction. JCI Insight.

[167] Williams S., Pourrier M., McAfee D., Lin S., Fedida D. (2014). Ranolazine improves diastolic function in spontaneously hypertensive rats. Am. J. Physiol. Heart Circ. Physiol..

[168] Wu B., Lin J., Luo J., Han D., Fan M., Guo T., Tao L., Yuan M., Yi F. (2017). Dihydromyricetin Protects against Diabetic Cardiomyopathy in Streptozotocin-Induced Diabetic Mice. BioMed Res. Int..

[169] Wu F., Qiu Y., Ye G., Luo H., Jiang J., Yu F., Zhou W., Zhang S., Feng J. (2015). Treatment with hydrogen molecule attenuates cardiac dysfunction in streptozotocin-induced diabetic mice. Cardiovasc. Pathol..

[170] Wu L., Mei L., Chong L., Huang Y., Li Y., Chu M., Yang X. (2016). Olmesartan ameliorates pressure overload-induced cardiac remodeling through inhibition of TAK1/p38 signaling in mice. Life Sci..

[171] Wu X., Zhang T., Lyu P., Chen M., Ni G., Cheng H., Xu G., Li X., Wang L., Shang H. (2021). Traditional Chinese Medication Qiliqiangxin Attenuates Diabetic Cardiomyopathy via Activating PPARγ. Front. Cardiovasc. Med..

[172] Xiao L., Gu Y., Gao L., Shangguan J., Chen Y., Zhang Y., Li L. (2017). Sanggenon C protects against pressure overload-induced cardiac hypertrophy via the calcineurin/NFAT2 pathway. Mol. Med. Rep..

[173] Xiao Y., Yang Z., Wu Q.Q., Jiang X.H., Yuan Y., Chang W., Bian Z.Y., Zhu J.X., Tang Q.Z. (2017). Cucurbitacin B Protects Against Pressure Overload Induced Cardiac Hypertrophy. J. Cell. Biochem..

[174] Xu C.N., Kong L.H., Ding P., Liu Y., Fan Z.G., Gao E.H., Yang J., Yang L.F. (2020). Melatonin ameliorates pressure overload-induced cardiac hypertrophy by attenuating Atg5-dependent autophagy and activating the Akt/mTOR pathway. Biochim. Biophys. Acta Mol. Basis Dis..

[175] Xu X., Hu X., Lu Z., Zhang P., Zhao L., Wessale J.L., Bache R.J., Chen Y. (2008). Xanthine oxidase inhibition with febuxostat attenuates systolic overload-induced left ventricular hypertrophy and dysfunction in mice. J. Card. Fail..

[176] Xu X., Zhang L., Liang J. (2013). Rosuvastatin prevents pressure overload-induced myocardial hypertrophy via inactivation of the Akt, ERK1/2 and GATA4 signaling pathways in rats. Mol. Med. Rep..

[177] Xu X., Zhao L., Hu X., Zhang P., Wessale J., Bache R., Chen Y. (2010). Delayed treatment effects of xanthine oxidase inhibition on systolic overload-induced left ventricular hypertrophy and dysfunction. Nucleosides Nucleotides Nucleic Acids.

[178] Xue M., Li T., Wang Y., Chang Y., Cheng Y., Lu Y., Liu X., Xu L., Li X., Yu X. (2019). Empagliflozin prevents cardiomyopathy via sGC-cGMP-PKG pathway in type 2 diabetes mice. Clin. Sci..

[179] Yamamoto M., Ishizu T., Seo Y., Suto Y., Sai S., Xu D., Murakoshi N., Kimura T., Kawakami Y., Aonuma K. (2018). Teneligliptin Prevents Cardiomyocyte Hypertrophy, Fibrosis and Development of Hypertensive Heart Failure in Dahl Salt-Sensitive Rats. J. Card. Fail..

[180] Yan P., Mao W., Jin L., Fang M., Liu X., Lang J., Jin L., Cao B., Shou Q., Fu H. (2020). Crude Radix Aconiti Lateralis Preparata (Fuzi) with Glycyrrhiza Reduces Inflammation and Ventricular Remodeling in Mice through the TLR4/NF-κB Pathway. Mediators Inflamm..

[181] Yan X., Zhang Y.L., Zhang L., Zou L.X., Chen C., Liu Y., Xia Y.L., Li H.H. (2019). Gallic Acid Suppresses Cardiac Hypertrophic Remodeling and Heart Failure. Mol. Nutr. Food Res..

[182] Yang L., Wu Q.Q., Liu Y., Hu Z.F., Bian Z.Y., Tang Q.Z. (2015). Cinnamaldehyde attenuates pressure overload-induced cardiac hypertrophy. Int. J. Clin. Exp. Pathol..

[183] Yin J., Kukucka M., Hoffmann J., Sterner-Kock A., Burhenne J., Haefeli W.E., Kuppe H., Kuebler W.M. (2011). Sildenafil preserves lung endothelial function and prevents pulmonary vascular remodeling in a rat model of diastolic heart failure. Circ. Heart Fail..

[184] Youcef G., Olivier A., Nicot N., Muller A., Deng C., Labat C., Fay R., Rodriguez-Guéant R.M., Leroy C., Jaisser F. (2016). Preventive and chronic mineralocorticoid receptor antagonism is highly beneficial in obese SHHF rats. Br. J. Pharmacol..

[185] Yu J., Chen R., Tan Y., Wu J., Qi J., Zhang M., Gu W. (2016). Salvianolic Acid B Alleviates Heart Failure by Inactivating ERK1/2/GATA4 Signaling Pathway after Pressure Overload in Mice. PLoS ONE.

[186] Yuan X., Xiao Y.C., Zhang G.P., Hou N., Wu X.Q., Chen W.L., Luo J.D., Zhang G.S. (2016). Chloroquine improves left ventricle diastolic function in streptozotocin-induced diabetic mice. Drug. Des. Devel. Ther..

[187] Yuan Y., Zong J., Zhou H., Bian Z.Y., Deng W., Dai J., Gan H.W., Yang Z., Li H., Tang Q.Z. (2014). Puerarin attenuates pressure overload-induced cardiac hypertrophy. J. Cardiol..

[188] Zhang B., Zhang J., Zhang C., Zhang X., Ye J., Kuang S., Sun G., Sun X. (2018). Notoginsenoside R1 Protects Against Diabetic Cardiomyopathy Through Activating Estrogen Receptor α and Its Downstream Signaling. Front. Pharmacol..

[189] Zhang N., Yang Z., Xiang S.Z., Jin Y.G., Wei W.Y., Bian Z.Y., Deng W., Tang Q.Z. (2016). Nobiletin attenuates cardiac dysfunction, oxidative stress and inflammatory in streptozotocin: Induced diabetic cardiomyopathy. Mol. Cell. Biochem..

[190] Zhang W.W., Bai F., Wang J., Zheng R.H., Yang L.W., James E.A., Zhao Z.Q. (2017). Edaravone inhibits pressure overload-induced cardiac fibrosis and dysfunction by reducing expression of angiotensin II AT1 receptor. Drug. Des. Devel. Ther..

[191] Zhang Y., Edgley A.J., Cox A.J., Powell A.K., Wang B., Kompa A.R., Stapleton D.I., Zammit S.C., Williams S.J., Krum H. (2012). FT011, a new anti-fibrotic drug, attenuates fibrosis and chronic heart failure in experimental diabetic cardiomyopathy. Eur. J. Heart Fail..

[192] Zhang Y., Lin X., Chu Y., Chen X., Du H., Zhang H., Xu C., Xie H., Ruan Q., Lin J. (2021). Dapagliflozin: A sodium-glucose cotransporter 2 inhibitor, attenuates angiotensin II-induced cardiac fibrotic remodeling by regulating TGFβ1/Smad signaling. Cardiovasc. Diabetol..

[193] Zhao H., Liao Y., Minamino T., Asano Y., Asakura M., Kim J., Asanuma H., Takashima S., Hori M., Kitakaze M. (2008). Inhibition of cardiac remodeling by pravastatin is associated with amelioration of endoplasmic reticulum stress. Hypertens. Res..

[194] Zhao M., Zhang J., Xu Y., Liu J., Ye J., Wang Z., Ye D., Feng Y., Xu S., Pan W. (2021). Selective Inhibition of NLRP3 Inflammasome Reverses Pressure Overload-Induced Pathological Cardiac Remodeling by Attenuating Hypertrophy, Fibrosis and Inflammation. Int. Immunopharmacol..

[195] Zhao T., Chen H., Xu F., Wang J., Liu Y., Xing X., Guo L., Zhang M., Lu Q. (2019). Liraglutide alleviates cardiac fibrosis through inhibiting P4hα-1 expression in STZ-induced diabetic cardiomyopathy. Acta Biochim. Biophys. Sin..

[196] Zhao T., Kee H.J., Kee S.J., Jeong M.H. (2022). Hdac8 inhibitor Alleviates Transverse Aortic Constriction-Induced Heart Failure in Mice by Downregulating Ace1. Oxid. Med. Cell. Longev..

[197] Zhao Y., Wang C., Wang C., Hong X., Miao J., Liao Y., Zhou L., Liu Y. (2018). An essential role for Wnt/β-catenin signaling in mediating hypertensive heart disease. Sci. Rep..

[198] Zhao Z., Liu H., Li Y., Tian J., Deng S. (2020). Wnt-C59 Attenuates Pressure Overload-Induced Cardiac Hypertrophy via Interruption of Wnt Pathway. Med. Sci. Monit..

[199] Zheng H., Pu S.Y., Fan X.F., Li X.S., Zhang Y., Yuan J., Zhang Y.F., Yang J.L. (2015). Treatment with angiotensin-(1-9) alleviates the cardiomyopathy in streptozotocin-induced diabetic rats. Biochem. Pharmacol..

[200] Zheng R.H., Bai X.J., Zhang W.W., Wang J., Bai F., Yan C.P., James E.A., Bose H.S., Wang N.P., Zhao Z.Q. (2019). Liraglutide attenuates cardiac remodeling and improves heart function after abdominal aortic constriction through blocking angiotensin II type 1 receptor in rats. Drug Des. Devel. Ther..

[201] Zhou G.F., Jiang Y.H., Ma D.F., Wang Y.C., Yang J.L., Chen J.Y., Chi C.Y., Han X.W., Li Z.Y., Li X. (2019). Xiao-Qing-Long Tang Prevents Cardiomyocyte Hypertrophy, Fibrosis and the Development of Heart Failure with Preserved Ejection Faction in Rats by Modulating the Composition of the Gut Microbiota. BioMed Res. Int..

[202] Zuo G., Ren X., Qian X., Ye P., Luo J., Gao X., Zhang J., Chen S. (2019). Inhibition of JNK and p38 MAPK-mediated inflammation and apoptosis by ivabrfoisadine improves cardiac function in streptozotocin-induced diabetic cardiomyopathy. J. Cell. Physiol..

[203] Hooijmans C.R., Rovers M.M., de Vries R.B., Leenaars M., Ritskes-Hoitinga M., Langendam M.W. (2014). SYRCLE’s risk of bias tool for animal studies. BMC Med. Res. Methodol..

[204] Seferović P.M., Coats A., Ponikowski P., Filippatos G., Huelsmann M., Jhund P.S., Polovina M.M., Komajda M., Seferović J., Sari I. (2020). European Society of Cardiology/Heart Failure Association position paper on the role and safety of new glucose-lowering drugs in patients with heart failure. Eur. J. Heart Fail..

[205] Tofovic S.P., Kusaka H., Kost C.K., Bastacky S. (2000). Renal function and structure in diabetic, hypertensive, obese ZDFxSHHF-hybrid rats. Ren. Fail..

[206] Roh J., Rhee J., Chaudhari V., Rosenzweig A. (2016). The Role of Exercise in Cardiac Aging: From Physiology to Molecular Mechanisms. Circ. Res..

[207] Jasińska-Stroschein M. (2022). Training programs in preclinical studies. The example of pulmonary hypertension. Systematic review and meta-analysis. PLoS ONE.

[208] Halliday B.P., Senior R., Pennell D.J. (2021). Assessing left ventricular systolic function: From ejection fraction to strain analysis. Eur. Heart J..

[209] Roh J., Houstis N., Rosenzweig A. (2017). Why Don’t We Have Proven Treatments for HFpEF?. Circ. Res..

[210] Ciccarelli M., Dawson D., Falcao-Pires I., Giacca M., Hamdani N., Heymans S., Hooghiemstra A., Leeuwis A., Hermkens D., Tocchetti C.G. (2021). Reciprocal organ interactions during heart failure: A position paper from the ESC Working Group on Myocardial Function. Cardiovasc. Res..

[211] Withaar C., Lam C.S.P., Schiattarella G.G., de Boer R.A., Meems L.M.G. (2021). Heart failure with preserved ejection fraction in humans and mice: Embracing clinical complexity in mouse models. Eur. Heart J..

