# Supplementary material for: Searching for Effective Treatments in HFpEF: Implications for Modeling the Disease in Rodents
Source: Pharmaceuticals (Basel). 2023 Oct 12;16(10):1449. doi: 10.3390/ph16101449 (PMC10610318; doi:10.3390/ph16101449)

Supplementary Material S1. Table S1. Study characteristics report.

First author (year)	Species	Animal Model	Animal Model – details	Pharmacologic class/ therapeutic group	Medication	Dose (/kg bw)	Drug administration period (weeks)
Abdellatif M (2021)	rat	ZSF1 Obese		Vitamin	Nicotinamide	40 mM*; i.p.*	12
Abdurrachim D (2019)	rat	SHHF		SGLT-2 inhibitor	Empagliflozin	25 mg; *i.g.*	25
Adams V (2022)	rat	ZSF1 Obese		MuRF1inhibitor	MyoMed-205	0.10%; *per os*	12
Akahori H (2014)	rat	Dahl salt sensitive	High-salt (HS, 8%)	HMGR inhibitor	Atorvastatin	20 mg; *i.g.*	7
Akinwumi BC (2017)	rat	SHHF		Plant-derivative	Gnetol<br>Pterostilbene<br>Resveratrol	2.5 mg; *i.g.*<br>2.5 mg; *i.g.*<br>2.5 mg; *i.g.*	8
Altara R (2020)	rat	Dahl salt sensitive	High-salt (HS, 8%)	ANP – derivative (RAAS compund)	Vastiras	100 ng; *s.c.*	6
Aroor AR (2021)	rat	ZSF1 Obese		AT1 antagonist (ARB)<br>NEP inhibitor/AT1 receptor antagonist (ARNI)<br>Vasodilator	Valsartan<br>Sacubitril/Valsartan<br>Hydralazine	31 mg; *i.g.*<br>68 mg; *i.g.*<br>30 mg; *i.g.*	10
Asensio Lopez MDC (2020)	mouse	STZ	50 mg *i.p.;* for three consecutive days	SGLT-2 inhibitor	Empagliflozin	10 mg; *per os*	8
Awwad ZM (2019)	rat	Pregabalin	10 mg/kg; *per os*	ACE inhibitor (RAAS compound)<br>AT1 receptor antagonist (ARB)	Captopril<br>Telmisartan	30 mg; *i.g.*<br>10 mg; *i.g.*	3
Bai XJ (2021)	rat	AAB	Pressure overload by AAB	GLP-1 receptor agonist	Liraglutide	0.3 mg; *s.c.* b.i.d	16
Bartoli F (2020)	mouse	TAC	Pressure overload by TAC	Inhibitor of ORAI1 ion channel subunit	C-dnO1	0.72 mg; *s.c.*	3
Benes J (2011)	rat	ACF	Pressure overload by ACF	Biguanide	Metformin	300 mg; *per os*	21
Biala A (2011)	rat	Dahl salt sensitive	High-salt (HS, 8%)	AT1 antagonist (ARB)<br>Calcium sensitizer	Valsartan<br>Levosimendan	1 mg; *per os*<br>1 mg; *per os*	8
Bryson TD (2020)	mouse	ANG II inf	1.4 µg/g/day *s.c.*, 14 days by an osmotic minipump	EP3 antagonist	L798,106	0.04 mg; *s.c.*	2
Bugyei-Twum A (2018)	mouse	TAC	Pressure overload by TAC	SIRT1 activator	SRT1720	100 mg; *i.g.*	5
Burke RM (2019)	mouse	TAC	Pressure overload by TAC	NEP inhibitor/AT1 receptor antagonist (ARNI)	Sacubitril/Valsartan	52/62 mg; *i.g.*	4
Byrne NJ (2020)	rat	Dahl salt sensitive	High-salt (HS, 8%)	SGLT-2 inhibitor	Empagliflozin	10 mg; *i.g.*	2

Cao HJ (2019)	mouse	uninephrectomized DOCA salt	After the left kidney was surgically removed, DOCA – as subcutaneous pellets (50 mg/pellet) plus 0.9% NaCl with drinking water for 21 days	β5i inhibitor	PR-957	50 mg*; i.p.*	3
Cappetta D (2020)	rat	Dahl salt sensitive	High-salt (HS, 8%)	SGLT-2 inhibitor	Dapagliflozin	0.1 mg; *i.g.*	11
Cezar MD (2015)	rat	SHR		MR antagonist (MRA)	Spironolactone	20 mg; *per os*	24
Chan V (2006)	rat	Uninephrectomized DOCA salt	After the left kidney was surgically removed, DOCA – as subcutaneous pellets (25 mg/pellet, every 4th day) plus 1% NaCl with drinking water for 28 days	-	Aminoguanidine	182 mg; *per os*	3
Chang D (2021)	rat	SNX		AT1 receptor antagonist (ARB)	Telmisartan	8 mg; *i.g.*	12
Chang SA (2009)	rat	Dahl salt sensitive	High-salt (HS, 8%)	HMGR inhibitor	Rosuvastatin	20 mg; *i.g.*	8
Chang X (2021)	mouse	TAC	Pressure overload by TAC	Plant-derivative	Quercetin	50 mg*; i.p.*	2
Chen C (2019)	mouse	TAC	Pressure overload by TAC	Plant-derivative	Resveratrol	50 mg; *i.g.*	2
Chen F (2015)	rat	STZ	70 mg/kg *i.p.*	Plant-derivative	Qiliqiangxin	80 mg; *i.g.*	8
Chen H (2022)	mouse	TAC	Pressure overload by TAC	Plant-derivative	Thymoquinone	50 mg; *per os*	6
Chen X (2022)	mouse	TAC	Pressure overload by TAC	Plant-derivative	Paeonol	50 mg; *i.g.*	4
Chen Y (2021)	mouse	ANG II inf	1.4 μg/g/day *s.c.*, 28 days by an osmotic minipump	Hormone	Lutein	100 mg	4
Chi L (2016)	rat	Dahl salt sensitive	High-salt (HS, 8%)	Late I(Na) inhibitor	GS-967	1 mg; *per os*	5
Chinnakkannu P (2018)	mouse	ANG II inf	2.1 μg/g/day *s.c.*, 14 days by an osmotic minipump	-	CSD peptide	0.05 mg; *s.c.*	2
Choudhary R (2008)	rat	AAB	Pressure overload by AAB	Vitamin	Retinoic acid	30 mg; *i.g.*	22
Davila A (2019)	rat	ZSF1 Obese		AK inhibitor	ABT-702	1.5 mg*; i.p.*	8 and 20
De Angelis (2016)	rat	Dahl salt sensitive	High-salt (HS, 8%)	Ranolazine	Ranolazine	20 mg*; i.p.*	8
Duda MK (2009)	rat	AAB	Pressure overload by AAB	-	ω-3 PUFA	7% energy ; *per os*	12
Dulce RA (2022)	mouse	ANG II inf	0.2 mg/kg/day *s.c.*, 28 or 56 days, by an osmotic minipump	Hormone	GHRH-A (MR-356)	0.2 mg; *s.c.*	4
Esposito G (2017)	rat	Dahl salt sensitive	High-salt (HS, 8%)	DPP-4 inhibitor	Sitagliptin	10 mg; *per os*	8
Evaristi MF (2022)	rat	ZSF1 Obese Adult<br>ZSF1 Obese Aged	Adult – 31 weeks;<br>Aged – 64 weeks	G-protein-biased S1P1 agonist	SAR247799	0.025%; *per os*	4
Fenning A (2005)	rat	Uninephrectomized DOCA salt	After the left kidney was surgically removed, DOCA – as subcutaneous pellets (25 mg/pellet, every	-	l-arginine	3400 mg; *per os*	4

			4th day) plus 1% NaCl with drinking water for 28 days				
Ge Q (2020)	mouse	TAC	Pressure overload by TAC	NEP inhibitor/AT1 receptor antagonist (ARNI)	Sacubitril/Valsartan	60 mg; *per os*	4
Gimenes R (2018)	rat	STZ	50 mg/kg *i.p.*	Plant-derivative	Apocynin	16 mg; *per os*	8
Giri SR (2016)	rat	ZDF +aortic constriction		PPAR-gamma agonist	Rosiglitazone	0.3 mg; *per os*<br>3 mg; *per os*<br>10 mg; *per os*	2
Gladden JD (2013)	rat	ACF	Pressure overload by ACF	XO inhibitor	Allopurinol	100 mg; *per os*	8
Goltsman I (2019)	rat	ACF	Pressure overload by ACF	PPAR-gamma agonist	Rosiglitazone	30 mg; *i.g.*	4
Gómez-Garre D (2010)	mouse	SHHF		HMGR inhibitor	Rosuvastatin	10 mg; *per os*	7
Gómez-Hurtado N (2017)	rat	TAC	Pressure overload by TAC	Hormone	Leptin	0.36 mg; *s.c.*	3
Gong W (2013)	rat<br>mouse	ISO + TAC	Isoprenaline at 5 mg/kg *s.c.*; 14 days	PDE-5 inhibitor	Sildenafil	50 mg *per os*<br>100 mg; *per os*	3
Gong W (2014)	rat	TAC	Pressure overload by TAC	PDE-5 inhibitor	Sildenafil	100 mg; *per os*	4
Gonzalez L (2018)	mouse	uninephrectomized DOCA salt	After the left kidney was surgically removed, DOCA at 60 mg/kg (two times per week; *i.m.*) plus 1% NaCl and 0.4% KCl in drinking water for 28 days	a Mas receptor blocker<br>RAAS compound	PD123319<br>ANG-(1–9)	28 ng/kg/min; *s.c.*<br>600 ng/kg/min; *s.c.*	4
Grune J (2016)	mouse	TAC	Pressure overload by TAC	MR antagonist (MRA)	Finerenone	10 mg*; i.p.*	4
Guan X (2020)	rat	SHR		BB<br>RAAS compound	Nebivolol<br>HB4	2.5 mg; *per os*<br>5 mg; *per os*<br>12.5 mg; *i.g.*<br>25 mg; *i.g.*	1
Habibi J (2017)	mouse	db/db		SGLT-2 inhibitor	Empagliflozin	10 mg; *per os*	5
Hamdani N (2014)	mouse	db/db		DPP-4 inhibitor	Sitagliptin	300 mg; *per os*	4
Hammoudi N (2017)	mouse	ob/ob		SGLT-2 inhibitor	Empagliflozin	10 mg; *per os*	6
Han X (2022)	mouse	TAB	Pressure-overload by TAB	Plant-derivative	EGCG	50 mg*; i.p.*	12
Henderson BC (2007)	mouse	AAB	Pressure overload by AAB	PPAR-gamma agonist	Ciglitazone	2 mg; *per os*	4
Horvath O (2021)	rat	SHR		Insulin sensitizer	BGP-15	25 mg; *per os*	18
Hou N (2019)	mouse	db/db+STZ	STZ at 45 mg/kg *i.p.* injections for five consecutive days	Plant-derivative	Carvacrol	20 mg*; i.p.*	6
Huang JP (2010)	rat	STZ	65 mg/kg *i.p.*	Plant-derivative	Resveratrol	0.1 mg; *i.g.*<br>1 mg; *i.g.*	3
Huang S (2021)	mouse	STZ	50 mg/kg *i.p.;* for five consecutive days	P2X7R inhibitor	A438079	10 mg*; i.p.*<br>20 mg*; i.p.*	16

Huang Y (2020)	mouse	L-NAME + HFD	L-NAME at 0.5g/l in drinking water; 60% kcal fat	Plant-derivative<br>SGLT-2 inhibitor	QiShenYiQi (QSYQ)<br>Dapagliflozin	1.16 mg; *i.g.*<br>1.52 mg; *i.g.*	14
Huang Y (2021)	mouse	TAC	Pressure overload by TAC	AT1 antagonist (RAAS compound)	Valsartan	10.4 mg; *i.g.*	4
Huc T (2018)	rat	SHR		Seafood derivative	Trimethylamine oxide	333 mg/l; *per os*	4
Huo S (2021)	mouse	TAC	Pressure overload by TAC	Raloxifene	Raloxifene	15 mg; *i.g.*	4<br>8
Ikeda J (2016)	rat	Isoproterenol	1 mg/kg *s.c.*, 14 days by an osmotic pump	DPP-4 inhibitor	Saxagliptin	17.5 mg; *per os*	2
Jackson MR (2022)	rat	TAC	Pressure overload by TAC	Inhibitor of human SQOR	STI1	10 mg*; i.p.*	12
Jeong EM (2013)	mouse	uninephrectomized DOCA salt	After the left kidney was surgically removed, DOCA – as subcutaneous pellets (0.7 mg/pellet) plus 1.05% NaCl in drinking water for 14-18 days	RAAS compound	HB4	200 mg; *per os*	1
Jeong MY (2018)	rat	Dahl salt sensitive	High-salt (HS, 4%)	HDAC inhibitor	ITF2357	3 mg; *per os*<br>30 mg; *per os*	10
Jiang X (2022)	mouse	TAC	Pressure overload by TAC	NLRP3 inflammasome inhibitor	HF-MCC950	10 mg*; i.p.*	3
Johnson JA (2011)	mouse	PAB		RAAS compund	rhACE2	1.8 mg; *s.c.*	2
Joubert M (2017)	mouse	seipin knockout (SKO)		PPAR-gamma agonist<br>SGLT-2 inhibitor	Pioglitazone<br>Dapagliflozin	300 mg; *per os*<br>1 mg; *i.g.*	4
Juric D (2007)	rat	AAB	Pressure overload by AAB	Plant-derivative	Resveratrol	2.5 mg; *i.g.*	2
Kakehi K (2019)	rat	Dahl salt sensitive	High-salt (HS, 8%)	BB<br>I(f) channel inhibitor	Bisoprolol<br>Ivabradine	4 mg; *per os*<br>10 mg; *per os*	16
Kamiya M (2021)	mouse	ANG II inf	1.4 mg/kg/day *s.c.*, 28 days by an osmotic minipump	BB	BRL 37344	2.4 mg; *s.c.*	4
Katare RG (2010)	mouse<br>rat	db/db<br>STZ	STZ at 40 mg/kg; *i.p.*	Vitamin	Benfotiamin	70 mg; *per os*	8<br>16
Khong FL (2011)	rat	STZ	55 mg/kg; *i.p.*	Plant-derivative	3',4'-Dihydroxyflavonol	1 mg; *i.g.*	6
Kim S (2001)	rat	Dahl salt sensitive	High-salt (HS, 7%)	ACE inhibitor (RAAS compound)<br>AT1 receptor antagonist (ARB)	Benazepril<br>Valsartan	10 mg; *i.g.*<br>30 mg; *i.g.*	10
Kim-Mitsuyama S (2004)	rat	Dahl salt sensitive	High-salt (HS, 8%)	ACE inhibitor (RAAS compound)<br>AT1 receptor antagonist (ARB)<br>CCB	Temocapril<br>Olmesartan<br>Azelnidipine	10 mg; *per os*<br>5 mg; *per os*<br>1 mg; *per os*	6

Lapinskas T (2020)	mouse	TAC	Pressure overload by TAC	Recombinant human relaxin1 hormone	Serelaxin	0.5 mg; *s.c.*	4
Lee HC (2019)	rat	SHR		SGLT-2 inhibitor	Empagliflozin	20 mg; *per os*	12
Leite S (2021)	rat	ZSF1		PDE-5 inhibitor	Sildenafil	100 mg; *per os*	20
Li L (2019)	mouse	STZ	50 mg/kg *i.p.;* for three consecutive days	Plant-derivative	Luteolin	20 mg; *s.c.*	15
Li X (2021)	mouse	TAC	Pressure overload by TAC	SGLT-2 inhibitor	Empagliflozin	10 mg; *i.g.*	4
Li Z (2020)	rat	Dahl salt sensitive	High-salt (HS, 8%)	Plant-derivative	Xiao-Qing-Long-Tang	1.2 mg; *i.g.*	4
Liao HH (2015)	mouse	AB	Pressure overload by AB	Plant derivative	Oleanolic acid	25 mg; *per os*<br>100 mg; *per os*	8
Liao Y (2008)	mouse	TAC	Pressure overload by TAC	HMGR inhibitor	Atorvastatin	5 mg; *i.g.*	4
Liu L (2010)	mouse	AAB	Pressure overload by AAB	AT1 receptor antagonist (ARB)	Telmisartan	5 mg; *i.g.*	12
Liu L (2018)	rat	TAC	Pressure overload by TAC	Plant-derivative	Epigallocatechin-3 gallate	50 mg*; i.p.*	8
Liu W (2013)	mouse	TAC	Pressure overload by TAC	Immunomodulator	FTY720	0.01 mg*; i.p.*	3
Liu XY (2018)	mouse	AAB	Pressure overload by AAB	Plant-derivative	Icariside II	10 mg; *i.g.*	6
Liu Y (2017)	rat	AAB	Pressure overload by AAB	-	Valproic acid	300 mg; *i.v.*	4
Loch D (2009)	rat	SHR		HMGR inhibitor	Rosuvastatin	20 mg; *i.g.*	24
Lou T (2021)	mouse	TAC	Pressure overload by TAC	Plant-derivative	Nuanxin	640 mg; *i.g.*	4
Louhelainen M (2007)	rat	Dahl salt sensitive	High-salt (HS, 7%)	Calcium sensitizer	Levosimendan	0.3 mg; *per os*<br>3 mg; *per os*	3.5 and 7
Louhelainen M (2009)	rat	Dahl salt sensitive	High-salt (HS, 8%)	Calcium sensitizer	OR-1986	0.05 mg; *per os*<br>0.5 mg; *per os*	3.5 and 7
Lovelock JD (2012)	mouse	uninephrectomized DOCA salt	After the left kidney was surgically removed, DOCA – as subcutaneous pellets (0.7 mg/pellet) plus 1.05% NaCl in drinking water for 14-18 days	Ranolazine	Ranolazine	5 mg; *per os*	1
Lu J (2013)	rat	STZ	55 mg/kg *i.p.*	Cu-selective chelator	Trientine	20 mg; *per os*	8
Luk FS (2016)	mouse	ApoE-/-<br>ApoE-/-+ HFD	16% kcal fat	Immunomodulator	FTY720	0.05 mg; *per os*	3.5
Ma S (2021)	mouse	TAC	Pressure overload by TAC	-	Nicotinamide Riboside	400 mg; *i.g.*	8
Ma XL (2019)	mouse	TAC	Pressure overload by TAC	CD20 antibody	Rituximab	75 µg/20 g *i.v.*	2
Ma Y (2016)	mouse	AAB	Pressure overload by AAB	Ang-(1-7) analog (RAAS compound)	AVE0991	20 mg; *per os*	4
Madonna R (2020)	mouse	STZ	150 mg/kg *i.p.*	SGLT-2 inhibitor	Empagliflozin	30 mg; *i.g.*	4
Mátyás C (2017)	rat	ZDF		PDE-5 inhibitor	Vardenafil	10 mg; *per os*	25
Methatham T (2021)	mouse	TAC	Pressure overload by TAC	β-catenin/CBP inhibitor	ICG001	50 mg*; i.p.*	1.5

Methawasin M (2020)	mouse	db/db<br>DOCA salt + TAC	DOCA at 0.7 mg *s.c.*, 28 days, by an osmotic minipump	PDE-9 inhibitor	PF-4449613	5 mg; *s.c.*<br>8 mg; *s.c.*	4
Minhas KM (2006)	rat	SHHF		XO inhibitor	Oxypurinol	1 mmol/L; *per os*	4
Mishra M (2018)	mouse	CC diet	28% kcal fat	Recombinant ApoA-I Milano	MDCO-216	100 mg*; i.p.*	26
Mishra M (2020)	mouse	ANG II inf	0.864 ug/g/day *s.c.*, 28 days by an osmotic minipump	-	Apo A-I (Milano) nanoparticles	100 mg; *s.c.*	4
Morgan LA (2013)	rat	TAC	Pressure overload by TAC	sEH inhibitor	GSK2188931	80 mg; *per os*	4
Muñoz-Pacheco P (2013)	rat	SHHF adult<br>SHHF aged	Adult – 16 weeks;<br>Aged – 80 weeks	MR antagonist (MRA)	Eplerenone	100 mg; *per os*	72
Naruse G (2019)	rat	Dahl salt sensitive	High-salt (HS, 8%)	alpha-glucosidase inhibitor<br>GLP-1	Miglitol<br>Exendin-4	100 mg; *per os*<br>2.2 nmol; *s.c.*	11
Nguyen TD (2018)	rat	AB	Pressure overload by AB	GLP-1 receptor agonist	GLP1	2 pmol/kg/min; *s.c.*	4
Nie J (2019)	mouse	TAC	Pressure overload by TAC	Ranolazine	Ranolazine	40 mg; *s.c.*	9
Nordén ES (2021)	rat	AB	Pressure overload by AB	NEP inhibitor/AT1 receptor antagonist (ARNI)	Sacubitril/Valsartan	68/31 mg; *i.g.*	8
Ohtani T (2007)	rat	Dahl salt sensitive	High-salt (HS, 8%)	MR antagonist (MRA)	Eplerenone	12.5 mg; *per os*<br>40 mg; *per os*	14
Oishi S (2017)	rat	Dahl salt sensitive	High-salt (HS, 8%)	NEP-resistant (NP) derivative	TDT	8 nmol; *s.c.*<br>40 nmol; *s.c.*	8
O'Shea KM (2010)	mouse	TAC	Pressure overload by TAC	-	ω-3 PUFA	*per os*	6
Park SH (2020)	rat	ZSF1 Obese		SGLT-2 inhibitor	Empagliflozin	30 mg; *per os*	6
Perlini S (2006)	rat	AAB	Pressure overload by AAB	Alpha 1 Adrenergic Receptor Antagonist<br>BB	Doxazosin<br>Propranolol	5 mg; *per os*<br>40 mg; *per os*	10
Plante E (2014)	mouse	db/db		NP	BNP	625 ng/kg h; *s.c.*	12
Pozder Geb Gehlken (2022)	mouse	ANG II inf	2.5 μg/g/day *s.c.*, 14 days by an osmotic minipump	Fruit derivative	*Rhubarb* pectin	1%; *per os*	2
Primessnig U (2019)	rat	SNX		Na+ /Ca2+ exchanger inhibitor	ORM-11035	1 mg; *i.g.*	16
Qiu Z (2012)	rat	SHR		HMGR inhibitor	Rosuvastatin	20 mg; *i.g.*<br>40 mg; *i.g.*	8
Randriamboavonjy JI (2016)	rat	SHR		Plant-derivative	*Moringa oleifera* seed powder	750 mg; *per os*	8
Reddy SS (2018)	mouse	HFD + ANG II inf	45% kcal fat; mice were infused with AngII (1500	PDE-3 inhibitor	Cilostazol	50 mg; *i.g.*	1

			ng/kg/min *s.c.*, 7 days) by an osmotic minipump				
Richards DA (2021)	mouse	TAC	Pressure overload by TAC	PDE-9 inhibitor	CRD-733	600; mg *i.g.*	2
Rimbaud S (2011)	rat	Dahl salt sensitive	High-salt (HS, 8%)	Plant-derivative	Resveratrol	18 mg; *per os*	8
Russell-Hallinan A (2021)	mouse	TAC	Pressure overload by TAC	Cytosine nucleoside analogue	5-Azacytidine	5 mg*; i.p.*	8
Salah EM (2018)	rat	ZSF1 Obese		ACE inhibitor (RAAS compound)<br>Loop diuretic	Captopril<br>Furosemide	100 mg; *per os*<br>50 mg; *per os*	24
Satoh S (2003)	rat	Dahl salt resistant	High-salt (HS, 8%)	ACE inhibitor (RAAS compound)	Temocapril	10 mg; *per os*	8
Satoh S (2003)	rat	Dahl salt sensitive	High-salt (HS, 8%)	ROCK inhibitor	Y-27632	5 mg; *per os*	11
Schauer A (2021)	rat	ZSF1 Obese		NEP inhibitor/AT1 receptor antagonist (ARNI)	Sacubitril/Valsartan	60 mg; *i.g.*	12
Seymour EM (2008)	rat	Dahl salt sensitive	High-salt (HS, 6%)	-<br>Vasodilator	Phytochemical-enriched diet<br>Hydralazaine	-<br>20 mg; *per os*	18
Shang L (2019)	mouse	TAC	Pressure overload by TAC	-	4-hydroxybenzylamine	1g/l; *per os*	6
Shao S (2021)	mouse	TAC	Pressure overload by TAC	I(f) channel inhibitor	Ivabradine	10 mg; *i.g.*<br>20 mg; *i.g.*	4
Shea CM (2020)	rat	Dahl salt sensitive	High-salt (HS, 8%)	sGC activator	Praliciguat	10 mg; *per os*	5
Shiraki A (2019)	hamster	J2N-k	J2N-k	GLP-1 receptor agonist	Liraglutide	0.02 mg; *s.c.*<br>0.1mg; *s.c.*	6
Signore PE (2021)	rat	NxZSF1 Obese	unilateral nephrectomy	PHD2 inhibitor	HIF-PHI FG-2216	40 mg; *i.g.*	18
Stolina M (2020)	rat	ZSF1 Obese		-	GDF15	1.5 mg; *s.c.*	12
Sukumaran V (2020)	rat	Zucker fatty rat+6%NaCl		GLP-1 receptor agonist	Liraglutide	0.1mg; *s.c.*	8
Sung YL (2020)	rat	SHR		AT1 antagonist (RAAS compound)<br>NEP inhibitor/AT1 antagonist (RAAS compound)	Valsartan<br>Sacubitril/Valsartan	160 mg; *i.g.*<br>98/102 mg; *i.g.*	2
Tamayo M (2020)	mouse	TAC	Pressure overload by TAC	Vitamin	Paricalcitol	300 mg*; i.p.*	5
Tang X (2017)	mouse	ANG II inf	1.3 µg/g/day *s.c.*, 28 days by an osmotic minipump	Biguanide	Metformin	200 mg; *per os*	4
Thandapilly SJ (2010)	rat	SHR	10 and 20 weeks of age	Plant-derivative	Resveratrol	2.5 mg; *i.g.*	10
Thomas TA (2005)	rat	SHHF		Hormone	TH	0.05%; *per os*<br>0.10%; *per os*<br>0.20%; *per os*	4

Tian J (2021)	rat	STZ+HFD	STZ at 35 mg/kg *i.p.*;<br>16.5% kcal fat	Biguanide<br>SGLT2	Metformin<br>Dapagliflozin	200 mg; *per os*<br>1 mg; *per os*	8
Travers JG (2021)	mouse	uninephrectomized DOCA salt	After the left kidney was surgically removed, DOCA – as subcutaneous pellets (25 mg/pellet) plus 0.9% NaCl and 0.2% KCl in drinking water for 28 days	HDAC inhibitor	ITF2357	50 mg; *per os*	4
Valero-Munoz M (2016)	mouse	uninephrectomized DOCA salt	After the left kidney was surgically removed, continuous infusion of *d*-aldosterone at 0.30 µg/h plus 0.9%NaCl in drinking water for 28 days	ERA	Macitentan	30 mg; *per os*	2
Venardos K (2015)	mouse	db/db		PKD inhibitor	CID755673	1 mg*; i.p.*<br>10 mg*; i.p.*	2
Verma S (2018)	mouse	db/db		SGLT-2 inhibitor	Empagliflozin	10 mg; *i.g.*	4
Wang D (2017)	rat	uninephrectomized DOCA salt	After the left kidney was surgically removed, DOCA – as subcutaneous pellets (50 mg/pellet) plus 0.9%NaCl and 0.2% KCl in drinking water for 28 days	Hormone	Serelaxin	16 mg; *s.c.*	4
Wang J (2017)	rat	SHR		AT1 receptor antagonist (ARB)<br>Plant-derivative	Olmesartan<br>Qiliqiangxin	2.5 mg; *i.g.*<br>500 mg; *i.g.*	8
Wang L (2020)	mouse	HFD	60% kcal fat	PGI2 analogue	Treprostinil	40 ng/min/kg; *s.c.*	16
Wei H (2017)	rat	STZ	60 mg/kg; *i.p.*	Vitamin	1,25D3	150 ng; *s.c.*	8
Westermann D (2007)	rat	STZ	50 mg/kg *i.p.;* for five consecutive days	eNOS enhancer	AVE3085	10 mg; *per os*	8
Westermann D (2009)	mouse	Dahl salt sensitive	High-salt (HS, 8%)	PDE-5 inhibitor	Sildenafil	100 mg; *per os*	8
Westermann D (2012)	mouse	ANG II inf	1.8 µg/g/day *s.c.*, 21 days by an osmotic minipump	AT1 receptor antagonist (ARB)	Irbesartan	20 mg; *per os*	2
Wilck N (2018)	rat	dTGR		sGC activator	BAY 41-8543	3 mg; *i.g.*	4
Williams S (2014)	rat	SHR		Ranolazine	Ranolazine	30 mg*; i.p.*	2
Wu B (2017)	mouse	STZ	50 mg/kg *i.p.;* for five consecutive days	Plant-derivative	Dihydromyricetin	100 mg; *i.g.*	14
Wu F (2015)	mouse	STZ	50 mg/kg *i.p.;* for five consecutive days	-	Hydrogen water	0.8 mg/l; *per os*	8

Wu L (2016)	mouse	TAC	Pressure overload by TAC	AT1 receptor antagonist (ARB)	Olmesartan	5.5 mg; *i.g.*	2
Wu X (2021)	mouse	STZ	50 mg/kg *i.p.;* for five consecutive days	Plant-derivative	Qiliqiangxin	500 mg; *i.g.*	9
Xiao L (2017)	mouse	AAB	Pressure overload by AAB	Plant-derivative	Sanggenon C	10 mg*; i.p.*<br>20 mg*; i.p.*	3
Xiao Y (2017)	mouse	TAC	Pressure overload by TAC	Plant-derivative	Cucurbitacin B	0.2 mg; *i.g.*	3
Xu CN (2020)	mouse	TAC	Pressure overload by TAC	Hormone	Melatonin	100 mg; *s.c.*	8
Xu X (2008)	rat	TAC	Pressure overload by TAC	XO inhibitor	Febuxostat	5 mg; *per os*	1
Xu X (2010)	mouse	TAC	Pressure overload by TAC	XO inhibitor	Allopurinol<br>Febuxostat	15 mg; *per os*<br>5 mg; *i.g.*	3
Xu X (2013)	mouse	AAB	Pressure overload by AAB	HMGR inhibitor	Rosuvastatin	10 mg; *i.g.*<br>20 mg; *i.g.*	4
Xue M (2019)	mouse	db/db		SGLT-2 inhibitor	Empagliflozin	10 mg; *i.g.*	8
Yamamoto M (2018)	rat	Dahl salt sensitive	High-salt (HS, 8%)	DPP-4 inhibitor	Teneligliptin	10 mg; *per os*	8 and 12
Yan P (2020)	mouse	TAC	Pressure overload by TAC	Plant-derivative	CFZ+GC	1.8 g/ml; *per os*	8
Yan X (2019)	mouse	TAC	Pressure overload by TAC	Plant-derivative	Gallic acid	5 mg; *i.g.*<br>20 mg; *i.g.*	8
Yang L (2015)	mouse	AAB	Pressure overload by AAB	Plant-derivative	Cinnamaldehyde	50 mg; *per os*	7
Yin J (2011)	rat	SAB		PDE-5 inhibitor	Sildenafil	60 mg; *per os*	9
Youcef G (2016)	rat	SHHF cp/cp obese		MR antagonist (MRA)	Eplerenone	100 mg; *per os*	44
Yu J (2016)	mouse	TAC	Pressure overload by TAC	BB<br>Plant-derivative	Metoprolol<br>Salvianolic acid	100 mg; *i.g.*<br>240 mg; *i.g.*	2
Yuan X (2016)	mouse	STZ	60 mg/kg *i.p.;* for three consecutive days	-	Chloroquine	60 mg*; i.p.*	2
Yuan Y (2014)	mouse	AB	Pressure overload by AB	Plant-derivative	Puerarin	65 mg; *per os*	7
Zhang B (2018)	mouse	db/db		Biguanide<br>Plant-derivative	Metformin<br>Notoginsenoside	200 mg; *i.g.*<br>7.5 mg; *i.g.*<br>15 mg; *i.g.*<br>30 mg; *i.g.*	20
Zhang N (2016)	mouse	STZ	50 mg/kg *i.p.;* for five consecutive days	Plant derivative	Nobiletin	50 mg; *i.g.*	11
Zhang WW (2017)	rat	TAC	Pressure overload by TAC	Antioxidant<br>AT1 receptor antagonist (ARB)	Edaravone<br>Telmisartan	10 mg*; i.p.*<br>10 mg*; i.p.*	8
Zhang Y (2012)	rat	(mRen-2)27 + STZ	STZ at 55 mg/kg *i.v.*	SGLT-2 inhibitor	Dapagliflozin	5 mg; *i.g.*	6
Zhang Y (2021)	rat	ANG II inf	0.75 µg/g/day *s.c.*, 28 days by an osmotic minipump	Anti-fibrotic	FT011	20 mg; *i.g.*	4
Zhao H (2008)	mouse	TAC	Pressure overload by TAC	HMGR inhibitor	Pravastatin	5 mg; *per os*	4
Zhao M (2021)	mouse	TAC	Pressure overload by TAC	NLRP3 inflammasome inhibitor	HF-MCC950	10 mg*; i.p.*	4
Zhao T (2019)	rat	STZ	pressure overload by TAC	GLP-1 receptor agonist	Liraglutide	0.09 mg; *s.c.*	16

Zhao T (2022)	mouse	TAC	Pressure overload by TAC	HDAC inhibitor	PCI34051	3 mg; *i.p.*<br>10 mg; *i.p.*<br>30 mg; *i.p.*	2
Zhao Y (2018)	rat	ANG II inf	0.6 μg/g/day *s.c.*, 28 days, by an osmotic minipump	AT1 receptor antagonist (ARB)<br>β-catenin/CBP inhibitor	Losartan<br>ICG-001	10 mg; *per os*<br>5 mg; *i.p.*	4
Zhao Z (2020)	mouse	TAC	Pressure overload by TAC	Porcupine inhibitor	Wnt-C59	5 mg; *i.g.*	4
Zheng H (2015)	rat	STZ	55 mg/kg *i.p.*	RAAS compund	ANG-(1–9)	200 ng; *s.c.*	4
Zheng RH (2019)	rat	AAB	Pressure overload by AAB	AT1 receptor antagonist (ARB)<br>GLP1	Telmisartan<br>Liraglutide	10 mg; *i.g.*<br>0.3 mg; *s.c.* b.i.d	16
Zhou GF (2019)	rat	Dahl salt sensitive	High-salt (HS, 8%)	Plant-derivative	Xiao-Qing-Long-Tang	4 g; per os	4
Zuo G (2019)	mouse	STZ	55 mg/kg *i.p.;* for five consecutive days	I(f) channel inhibitor	Ivabradine	20 mg; *i.g.*	12

1,25 D3 – 1,25-Dihydroxyvitamin-D3; AAB – Abdominal Aortic Banding; AB – Aortic Banding; ACE – Angiotensin Converting Enzyme; ACF – Aortocaval Fistula; AK – Adenosine Kinase; Ald – Aldosterone; Ang – Angiotensin; ANP – Atrial Natriuretic Peptide; ApoA – Apolipoprotein A; ARB – Angiotensin Receptor Blocker; AT1 – Angiotensin II Receptor Type 1; b.i.d – twice daily; BNP – Brain Natriuretic Peptide; CC – coconut oil; CSD – Caveolin-1 scaffolding domain; DM − Diabetes Mellitus; DOCA − Deoxycorticosterone Acetate; DPP-4 – Dipeptidyl Peptidase-4; dTGR – double-transgenic rats harboring the human renin and angiotensinogen genes; EGCG – Epigallocatechin-3-gallate; eNOS – Endothelial Nitric Oxide Synthase; EP3 – Prostaglandin Receptor; ERA – Endothelin-1 (ET-1) Receptor Antagonist; GDF15 – Growth/differentiation factor 15; GLP-1 – Glucagon-Like Peptide 1; HB4 – Tetrahydrobiopterin; HDAC – Histone Deacetylase; HFD − High Fat Diet; HMGR – (HMG-CoA) Reductase; ISO – Isoprenaline; L-NAME – L-N^{G}-Nitro arginine methyl ester; MR − Mineralocorticoid-Receptor; MS − Metabolic Syndrome; MuRF1 − Muscle RING-finger protein-1; NEP – Neprilysin; NLRP3 – Nod-Like Receptor Family Pyrin Domain-Containing 3; NP – Natriuretic Peptide; P2X7R – P2X purinoceptor 7; PAB – Pulmonary Aortic Banding; PDE – Phosphodiesterase; PGI2 – Prostacyclin; PHD2 – HIF prolyl hydroxylase-2; PKD – Protein Kinase D; PPAR – Peroxisome Proliferator-Activated Receptor; PUFA – Polyunsaturated Fatty Acids; RAAS – Renin–Angiotensin–Aldosterone System; RD − Regular Diet; rhACE2 – Recombinant Human Angiotensin-Converting Enzyme 2; ROCK – Rho-Associated Protein Kinase; S1P1 – Sphingosine-1-Phosphate Receptor 1; SAB – Supracoronary Aortic Banding; sEH – Soluble Epoxide Hydrolase; sGC – Soluble Guanylyl Cyclase; SGLT-2 – Sodium-glucose co-transporter-2; SHHF – Spontaneously Hypertensive Heart Failure; SHR − Spontaneously Hypertensive Rat; SIRT1 – Sirtuin 1; SNX – subtotal nephrectomy; SQOR – Sulfide:quinone oxidoreductase; STZ − Streptozotocin; TAB – Thoracic Aortic Banding; TAC − Transverse Aortic Constriction; TH – Thyroid Hormone; XO – Xanthine Oxidase; ZDF – Zucker Diabetic Fatty

Supplementary Material S1. Table S2. Results from publication bias funnel plot and 'trim and fill analysis'

**Active substance/class**	**Animal model of HF***	**Parameter (effect size)**	**Egger regression P-value**	**Imputed (trim and fill)**
All	All	Active relaxation (R, response ratio)	0.006	0
		Passive stiffness (R, response ratio)	NS	0
		LVESd (D, difference in means)	NS	0
		LVEDs (D, difference in means)	NS	0
		LVSP (D, difference in means)	NS	1
		EF (D, difference in means)	NS	0
		Lung congestion (R, response ratio)	NS	0
		LVH (R, response ratio)	NS	0
		Fibrosis (R, response ratio)	<0.0001	0
AT1 receptor antagonist (ARB)	Ang II, Dahl salt sensitive, pressure-overload by AAB (TAC), SHR, STZ, ZSF1 Obese	Active relaxation (R, response ratio)	0.068	1
Beta-blocker (BB)	Ang II		0.045	0
DPP-4 inhibitor	Dahl salt sensitive, db/db		NS	0
GLP-1 agonist	Dahl salt sensitive, pressure-overload by AAB, STZ, Zucker fatty		NS	0
HMGR inhibitor	Dahl salt sensitive, pressure overload by AAB (TAC), SHHF, SHR		NS	0
Plant-derivative	Pressure overload by AAB (TAC), SHR, STZ		NS	0
SGLT-2 inhibitor	Dahl salt sensitive, db/db (ob/ob), pressure-overload by TAC, SHR, STZ (+HFD)		NS	0
DPP-4 inhibitor	Dahl salt sensitive, db/db	Passive stiffness (R, response ratio)	NS	0
GLP-1 receptor agonist	Dahl salt sensitive, pressure-overload by AAB, STZ		NS	0
PDE3-, PDE5-, PDE9-inhibitor	db/db, DOCA salt (+TAC), ZSF1, ZDF		NS	0
AT1 receptor antagonist (ARB)	Dahl salt sensitive, pressure-overload by AAB (TAC), SHR	LVEDd (D, difference in means)	NS	0
Calcium sensitizer	Dahl salt sensitive		NS	0
DPP-4 inhibitor	Dahl salt sensitive, Isoproterenol-induced HF		NS	0
HMGR inhibitor	Dahl salt sensitive, pressure overload by AAB (TAC), SHHF, SHR		NS	0
MR antagonist (MRA)	Dahl salt sensitive, SHHF aged, SHHF cp/cp obese, SHR		0.028	0
Plant-derivative	Dahl salt sensitive, db/db, pressure overload by AAB (TAC), STZ, SHR		NS	0
Calcium sensitizer	Dahl salt sensitive	LVESd (D, difference in means)	0.067	1
Plant-derivative	pressure overload by AAB (TAC), STZ, SHR		NS	0

AT1 receptor antagonist	Ang II, Dahl salt sensitive, pressure-overload by AAB (TAC), SHR, STZ, ZSF1 Obese	EF (D, difference in means)	NS	0
NEP inhibitor/AT1 receptor antagonist (ARNI)	Pressure-overload by AB (TAC), SHR, ZSF1 Obese		NS	0
Beta-blocker (BB)	Ang II, Dahl salt sensitive, Pressure-overload by AB (TAC),		NS	0
Calcium sensitizer	Dahl salt sensitive		0.0002	0
DPP-4 inhibitor	Dahl salt sensitive, Isoproterenol-induced HF		0.017	0
GLP-1 receptor agonist	Dahl salt sensitive, pressure overload by AAB (TAC), STZ, Zucker fatty rat		NS	0
HMGR inhibitor	Dahl salt sensitive, pressure overload by AAB (TAC), SHHF, SHR		NS	0
MR antagonist (MRA)	Dahl salt sensitive, SHR, SHHF aged (SHHF cp/cp obese)		NS	0
PDE3-, PDE5-, PDE9-inhibitor	Ang II(+HFD), db/db, DOCA+TAC, ISO + TAC, pressure overload by TAC, ZSF1, ZDF		NS	0
SGLT-2 inhibitor	Ang II, Dahl salt sensitive, db/db (ob/ob), L-Name+HFD, pressure-overload by TAC, SHR, STZ (+HFD), SHHF, ZSF1 Obese		NS	0
ACE inhibitor	Dahl salt sensitive	LVH (R, response ratio)	NS	0
AT1 receptor antagonist (ARB)	Ang II, Dahl salt sensitive, pressure-overload by AAB (TAC), STZ, ZSF1 Obese		NS	0
DPP-4 inhibitor	Dahl salt sensitive, db/db		NS	0
GLP-1 receptor agonist	Dahl salt sensitive, STZ, Zucker fatty rat		NS	0
HMGR inhibitor	Dahl salt sensitive, pressure overload by AAB (TAC), SHHF		NS	0
PDE3-i, PDE5-i, PDE9-i	Ang II(+HFD), ISO + TAC, pressure overload by TAC, ZSF1, ZDF		NS	0
Plant-derivative	Dahl salt sensitive, db/db, L-Name+HFD, pressure overload by AAB (TAC), STZ		NS	0
SGLT-2 inhibitor	Ang II, Dahl salt sensitive, db/db (ob/ob), pressure-overload by TAC, SHR, STZ (+HFD), SHHF		NS	0
AT1 receptor antagonist (ARB)	Dahl salt sensitive, pressure-overload by AAB (TAC), SHR, STZ	Fibrosis (R, response ratio)	NS	0
DPP-4 inhibitor	Dahl salt sensitive, Isoproterenol-induced HF		0.013	1
Plant-derivative	Dahl salt sensitive, pressure overload by AAB (TAC), SHHF, SHR, STZ		NS	3
Plant-derivative	Dahl salt sensitive, pressure overload by AAB (TAC), STZ	Lung congestion (R, response ratio)	NS	0

* – Baseline mean LV ejection fraction was equal or higher than 50% in heart failure group (Vehicle).

Supplementary Material S1. Table S3. Detailed characteristics of animal models used for experimental protocols to evaluate efficacy of particular medicine agent for HF. Increased D (R>1) values for comparison Vehicle vs Sham indicate worsening (**W**) of HF in relation to healthy subjects (Sham); decreased D (R<1) values – indicate weaker potential of animal model to promote disease development, according to particular parameter. D – difference in means; R – response ratio; $\bar{T}$ – mean EF in Vehicle group. * – studies where baseline mean ejection fraction was equal or higher than 50% were considered, only.

**Animal model ***	**Parameter**	**Effect size (95%CI)**
Ald II inf	Baseline LVEF%*	$\bar{T}$=67.24 (65.04; 69.44)
	↑ LVSP (**W**)	D=11.86 (9.93; 13.79); P<0.0001
	↔ LVESd	D=-0.1 (-0.23; 0.03); NS
	↔ LVEDd	D=-0.02 (-0.15; 0.11); NS
	↔ Passive stiffness	R=1.29 (0.77; 2.16); P<0.0001
Ang II inf (+HFD)	Baseline LVEF%	$\bar{T}$ = 62.47 (54.27; 70.67)
	↔ LVSP	D=9.61 (-0.78; 20.00); NS
	↓ dP/dt_{max}	D=-525.32 (-804.12; -246.52); P=0.0002
	↔ LVEDd	D=-0.55 (-1.69; 0.60); NS
	↔ Active relaxation	R=1.04 (0.93; 1.17); NS
	↑ Passive stiffness (**W**)	R=1.29 (0.98; 1.69); P=0.066
	↑ LVH (**W**)	R=1.31 (1.25; 1.37); P<0.0001
	↑ Fibrosis (**W**)	R=3.83 (2.55; 5.74); P<0.0001
	↑ Lung congestion (**W**)	R=1.06 (1.02; 1.09); P=0.0006
Dahl-salt sensitive	Baseline LVEF%	$\bar{T}$ = 70.48 (68.00; 72.97)
	↔ LVSP	D=7.94 (-25.33; 41.21); NS
	↔ dP/dt_{max}	D=-126.91 (-2577.77; 2323.94); NS
	↑ LVESd (**W**)	D=0.44 (-0.0004; 0.87); P=0.05
	↔ LVEDd	D=-0.19 (-0.53; 0.15); NS
	↑ Active relaxation	R=1.37 (1.13; 1.67); P=0.001
	↑ Passive stiffness (**W**)	R=1.07 (1.02; 1.13); P=0.005
	↑ LVH (**W**)	R=1.45 (1.39; 1.51); P<0.0001
	↑ Fibrosis (**W**)	R=3.00 (1.98; 4.56); P<0.0001
	↑ Lung congestion (**W**)	R=1.55 (1.41; 1.71); P<0.0001
db/db (ob./ob.)	Baseline LVEF%	$\bar{T}$ = 60.59 (56.12; 65.06)
	↔ dP/dt_{max}	D=282 (-400.44; 964.44); NS
	↑ LVEDd (**W**)	D=0.4 (0.31; 0.49); P<0.0001
	↔ Active relaxation	R=1.06 (0.90; 1.24); NS
	↑ Passive stiffness (**W**)	R=1.47 (1.27; 1.71); P<0.0001
	↑ Fibrosis (**W**)	R=3.00 (1.98; 4.56); P<0.0001
	↑ Lung congestion (**W**)	R=1.06 (1.06; 1.07); P<0.0001
DOCA-salt infused to uninephrectomized subjects	Baseline LVEF%	$\bar{T}$ = 76.01 (67.02; 84.99)
	↔ dP/dt_{max}	D=-47.48 (-880.45; 785.49); NS
	↔ LVESd	D=0.35 (-0.22; 0.92); NS
	↔ LVEDd	D=0.44 (-0.15; 1.04); NS
	↔ Active relaxation	R=0.86 (0.73; 1.01); NS
	↑ Passive stiffness (**W**)	R=1.16 (1.08; 1.25); P<0.0001
	↑ LVH (**W**)	R=1.36 (1.14; 1.63); P=0.0005
	↑ Fibrosis (**W**)	R=1.93 (1.55; 2.40); P<0.0001
	↑ Lung congestion (**W**)	R=1.12 (1.10; 1.15); P<0.0001
Isoproterenol	Baseline LVEF%	$\bar{T}$ = 70.22 (62.14; 78.31)
	↑ Active relaxation	R=1.14 (1.11; 1.17); P<0.0001
	↑ LVESd (**W**)	D=0.38 (0.26; 0.50); P<0.0001
	↑ LVEDd (**W**)	D=2.11 (1.99; 2.23); P<0.0001
	↑ Passive stiffness (**W**)	R=1.05 (1.03; 1.07); P<0.0001
	↑ Fibrosis (**W**)	R=1.14 (1.07; 1.22); P<0.0001
L-Name+HFD	Baseline LVEF%	$\bar{T}$ = 67.99 (67.01; 68.97)

	↑ LVH (**W**)	R=1.67 (1.43;1.94); P<0.0001
Nephrectomy	Baseline LVEF%	Ŧ = 76.88 (72.97; 80.80)
	↑ Active relaxation	R=1.71 (1.53; 1.92); P<0.0001
	↑ Fibrosis (**W**)	R=8.00 (2.63; 15.23); P<0.0001
	↑ LVH (**W**)	R=1.34 (1.28; 1.40); P<0.0001
	↑ Lung congestion (**W**)	R=1.50 (1.43; 1.57); P<0.0001
Pressure overload by AB (TAB/TAC)	Baseline LVEF%	Ŧ = 62.13 (59.77; 64.48)
	↔LVSP	D=7.21 (-18.91; 33.32); NS
	↓ dP/dt_{max}	D=-1179.03 (-1738.46; -619.59); P<0.0001
	↑ LVESd (**W**)	D=1.12 (0.91; 1.34); P<0.0001
	↑ LVEDd (**W**)	D=1.13 (0.96; 1.31); P<0.0001
	↓ Active relaxation (**W**)	R=0.78 (0.72; 0.85); P<0.0001
	↑ Passive stiffness (**W**)	R=1.25 (1.19; 1.30); P<0.0001
	↑ LVH (**W**)	R=1.54 (1.46; 1.62); P<0.0001
	↑ Fibrosis (**W**)	R=3.90 (2.98; 5.11); P<0.0001
	↑ Lung congestion (**W**)	R=1.56 (1.41; 1.71); P<0.0001
SHHF	BaselineLVEF%	Ŧ = 72.61 (67.04; 78.17)
	↓ dP/dt_{max}	D=-2062 (-2923.25; -1200.74); P<0.0001
	↑ LVEDs (**W**)	D=1.32 (0.44; 2.20); P=0.003
	↔ LVEDd	D=0.45 (-0.35; 1.26); NS
	↔ Active relaxation	R=0.91 (0.79; 1.05); NS
	↔ Passive stiffness	R=1.07 (0.95; 1.21); NS
	↑ LVH (**W**)	R=1.74 (1.5; 1.95); P<0.0001
	↑ Fibrosis (**W**)	R=4.70 (1.93; 11.48); P=0.0007
	↔ Lung congestion	R=1.19 (0.94; 1.51); NS
SHR	Baseline LVEF%	Ŧ =76.00 (71.69; 80.31)
	↑ LVSP (**W**)	D=45.05 (40.17; 49.93); P<0.0001
	↔ LVESd	D=0.0016 (-0.04; 0.045); NS
	↔ LVEDd	D=0.36 (-0.12; 0.85); NS
	↔ Active relaxation	D=1.07 (0.95; 1.21); NS
	↑ Passive stiffness (**W**)	R=1.08 (1.00; 1.17); P=0.049
	↑ LVH (**W**)	R=1.47 (1.18; 1.84); P=0.0005
	↑ Fibrosis (**W**)	R=2.61 (2.07; 3.29); P<0.0001
	↑ Lung congestion (**W**)	R=1.56 (1.22; 1.99); P=0.0003
STZ	Baseline LVEF%	Ŧ = 64.64 (60.13; 69.15)
	↓ LVSP	D=-30.24 (-42.18; -18.30); P<0.0001
	↓ dP/dt_{max}	D=-3244.16 (-6057.78; -430.55); P=0.023
	↑ LVESd (**W**)	D=0.91 (0.63; 1.18; P<0.00001
	↑ LVEDd (**W**)	D=0.50 (0.21; 0.91); P=0.016
	↓ Active relaxation (**W**)	R=0.78 (0.66; 0.93); P=0.005
	↑ Passive stiffness (**W**)	R=1.26 (0.98; 1.63); P=0.07
	↑ LVH (**W**)	R=1.23 (1.05; 1.45); P=0.01
	↑ Fibrosis (**W**)	R=2.01 (1.12; 3.59); P=0.019
	↔ Lung congestion	R=1.90 (0.69; 5.27); NS
ZDF	Baseline EF%	Ŧ = 69.12 (67.70; 70.54)
	↓ dP/dt_{max}	D=-948 (-1306.59; -589.41); P<0.0001
	↔ Active relaxation	R=0.97 (0.89; 1.07); NS
	↑ Passive stiffness(**W**)	R=1.86 (1.79; 1.93); P<0.0001
	↑ LVH (**W**)	R=1.07 (1.01; 1.14); P=0.012
ZSF1 Obese	Baseline LVEF%	Ŧ = 72.10 (69.57; 74.67)
	↔ LVSP	D=28.71 (-3.17; 60.59); NS
	↔ dP/dt_{max}	D=2072.45 (-191.75; 4336.64); P=0.07
	↑ LVEDd (**W**)	D=1.3 (1.19; 1.41); P<0.0001
	↑ Active relaxation	R=1.13 (1.04; 1.23); P=0.003

	↑ Passive stiffness (**W**)	R=1.88 (1.45; 2.44); P<0.0001
	↑ LVH (**W**)	R=1.29 (1.22; 1.37); P<0.0001
	↑ Fibrosis (**W**)	R=1.76 (1.14; 2.73); P=0.01
	↑ Lung congestion (**W**)	R=1.58 (1.48; 1.69); P<0.0001
Zucker fatty	Baseline LVEF%	$\bar{X}$ = 67.24 (65.04; 69.44)
	↑ Fibrosis (**W**)	R=4.14 (3.63; 4.72); P<0.0001

Supplementary Material S1. Table S4. The impact of baseline ejection fraction in Vehicle animals on the worsening/normalization of HF-related parametrs in subjects with HF receiving placebo (Vehicle) and medicine agents (Treatment). Increased D (R>1) values for comparison: Vehicle vs Sham indicate worsening (**W**) of HF in relation to healthy subjects (Sham); decreased D (R<1) values for comparison Treatment vs Vehicle indicate more normalization (**N**) of the disease in relation to HF subjects receiving placebo (Vehicle). For the majority of parameters except from active relaxation, fibrosis and LVSP, the development of more deleterious disease was accompanied by the baseline LVEF below threshold of 50%; similarly, the medicine agents normalized the disease more in subjects with LVEF<50%. Statistically significant Q measure (P<0.05) indicates the difference between groups (i.e., <50% vs ≥50%).

**Animal model (baseline LVEF)**	**Animal group**	**Parameter (Effect size)**	**Effect size (95%CI)**	**Comparison**
<50%	Vehicle vs Sham	LVEDd (D, difference in means)	1.33 (0.93; 1.73); P<0.0001 (**W**)	Q=16.49; df=1; P<0.0001
≥50%			0.42 (0.25; 0.59); P<0.0001 (**W**)	
<50%	Treatment vs Vehicle		-0.57 (-0.83; -0.31); P<0.0001 (**N**)	Q=5.00; df=1; P=0.025
≥50%			-0.24 (-0.37; -0.11); P=0.0002 (**N**)	
<50%	Vehicle vs Sham	LVESd (D, difference in means)	2.16 (1.53; 2.80); P<0.0001 (**W**)	Q=19.57; df=1; P<0.0001
≥50%			0.70 (0.54; 0.85); P<0.0001 (**W**)	
<50%	Treatment vs Vehicle		-0.94 (-1.33; -0.55); P<0.0001 (**N**)	Q=4.08; df=1; P=0.043
≥50%			-0.50 (-0.67; -0.33); P<0.0001 (**N**)	
<50%	Vehicle vs Sham	Active relaxation (R, response ratio)	0.84 (0.70; 1.01); P=0.059 (**W**)	Q=3.52 df=1; P=0.043
≥50%			1.01 (0.95; 1.07); NS	
<50%	Treatment vs Vehicle		1.10 (0.96; 1.25); NS	Q=0.034; df=1; NS
≥50%			1.08 (1.04; 1.13); P<0.0001 (**N**)	
<50%	Vehicle vs Sham	Passive stiffness (R, response ratio)	1.39 (1.29; 1.50); P<0.0001 (**W**)	Q=10.07; df=1; P=0.0015
≥50%			1.21 (1.17; 1.25); P<0.0001 (**W**)	
<50%	Treatment vs Vehicle		0.62 (0.57; 0.68); P<0.0001 (**N**)	Q=4.70; df=1; P=0.030
≥50%			0.70 (0.66; 0.74); P<0.0001 (**N**)	
<50%	Vehicle vs Sham	Lung congestion (R, response ratio)	1.42 (1.14; 1.76); P<0.0001 (**W**)	Q=0.078; df=1; NS
≥50%			1.47 (1.39; 1.55); P<0.0001 (**W**)	
<50%	Treatment vs Vehicle		0.71 (0.59; 0.84); P=0.0001 (**N**)	Q=4.21; df=1; P=0.040
≥50%			0.86 (0.82; 0.89); P<0.0001 (**N**)	
<50%	Vehicle vs Sham	LVH (R, response ratio)	1.66 (1.49; 1.85); P<0.0001 (**W**)	Q=34.69; df=1; P<0.0001
≥50%			1.13 (1.07; 1.20); P<0.0001(**W**)	
<50%	Treatment vs Vehicle		0.82 (0.80; 0.86); P<0.0001 (**N**)	Q=8.67; df=1; P=0.0030
≥50%			0.91 (0.86; 0.96); P=0.0007 (**N**)	
<50%	Vehicle vs Sham	Fibrosis (R, response ratio)	5.49 (3.97; 7.58); P<0.0001 (**W**)	Q=9.90; df=1; P=0.0016
≥50%			3.01 (2.50; 3.63); P<0.0001 (**W**)	
<50%	Treatment vs Vehicle		0.56 (0.45; 0.68); P<0.0001 (**N**)	Q=0.26; df=1; NS
≥50%			0.91 (0.86; 0.96); P<0.0001 (**N**)	
<50%	Vehicle vs Sham	LVSP (D, difference in means)	1.17 (0.92; 1.48); NS	Q=0.26; df=1; NS
≥50%			1.09 (1.01; 1.19); P=0.031 (**W**)	
<50%	Treatment vs Vehicle		0.97 (0.91; 1.03); NS	Q=2.11; df=1; NS
≥50%			1.027 (0.99; 1.07); NS	

Supplementary Material S1. Table S5. PRISMA Checklist.

Section and Topic	Item #	Checklist item	Location where item is reported
**TITLE**			
Title	1	Identify the report as a systematic review.	Page 1
**ABSTRACT**			
Abstract	2	See the PRISMA 2020 for Abstracts checklist.	Page 1
**INTRODUCTION**			
Rationale	3	Describe the rationale for the review in the context of existing knowledge.	Page 1-2
Objectives	4	Provide an explicit statement of the objective(s) or question(s) the review addresses.	
**METHODS**			
Eligibility criteria	5	Specify the inclusion and exclusion criteria for the review and how studies were grouped for the syntheses.	Page 2, Appendix A
Information sources	6	Specify all databases, registers, websites, organisations, reference lists and other sources searched or consulted to identify studies. Specify the date when each source was last searched or consulted.	Page 2
Search strategy	7	Present the full search strategies for all databases, registers and websites, including any filters and limits used.	Page 2; Appendix A
Selection process	8	Specify the methods used to decide whether a study met the inclusion criteria of the review, including how many reviewers screened each record and each report retrieved, whether they worked independently, and if applicable, details of automation tools used in the process.	Page 2
Data collection process	9	Specify the methods used to collect data from reports, including how many reviewers collected data from each report, whether they worked independently, any processes for obtaining or confirming data from study investigators, and if applicable, details of automation tools used in the process.	Page 4
Data items	10a	List and define all outcomes for which data were sought. Specify whether all results that were compatible with each outcome domain in each study were sought (e.g. for all measures, time points, analyses), and if not, the methods used to decide which results to collect.	Page 2-3
	10b	List and define all other variables for which data were sought (e.g. participant and intervention characteristics, funding sources). Describe any assumptions made about any missing or unclear information.	Page 2-3
Study risk of bias assessment	11	Specify the methods used to assess risk of bias in the included studies, including details of the tool(s) used, how many reviewers assessed each study and whether they worked independently, and if applicable, details of automation tools used in the process.	Page 4
Effect measures	12	Specify for each outcome the effect measure(s) (e.g. risk ratio, mean difference) used in the synthesis or presentation of results.	Page 3
Synthesis methods	13a	Describe the processes used to decide which studies were eligible for each synthesis (e.g. tabulating the study intervention characteristics and comparing against the planned groups for each synthesis (item #5)).	Page 3
	13b	Describe any methods required to prepare the data for presentation or synthesis, such as handling of missing summary statistics, or data conversions.	Page 3-4
	13c	Describe any methods used to tabulate or visually display results of individual studies and syntheses.	Page 3-4
	13d	Describe any methods used to synthesize results and provide a rationale for the choice(s). If meta-analysis was performed, describe the model(s), method(s) to identify the presence and extent of statistical heterogeneity, and software package(s) used.	Page 3-4
	13e	Describe any methods used to explore possible causes of heterogeneity among study results (e.g. subgroup analysis, meta-regression).	Page 4
	13f	Describe any sensitivity analyses conducted to assess robustness of the synthesized results.	-

Section and Topic	Item #	Checklist item	Location where item is reported
Reporting bias assessment	14	Describe any methods used to assess risk of bias due to missing results in a synthesis (arising from reporting biases).	Page 4
Certainty assessment	15	Describe any methods used to assess certainty (or confidence) in the body of evidence for an outcome.	-
**RESULTS**			
Study selection	16a	Describe the results of the search and selection process, from the number of records identified in the search to the number of studies included in the review, ideally using a flow diagram.	Page 5, Fig 1, Table S1
	16b	Cite studies that might appear to meet the inclusion criteria, but which were excluded, and explain why they were excluded.	Page 5
Study characteristics	17	Cite each included study and present its characteristics.	Table S3; Appendix B
Risk of bias in studies	18	Present assessments of risk of bias for each included study.	Page 6; Table 2
Results of individual studies	19	For all outcomes, present, for each study: (a) summary statistics for each group (where appropriate) and (b) an effect estimate and its precision (e.g. confidence/credible interval), ideally using structured tables or plots.	Table 1; Fig 3
Results of syntheses	20a	For each synthesis, briefly summarise the characteristics and risk of bias among contributing studies.	Page 5, 6; Fig 2
	20b	Present results of all statistical syntheses conducted. If meta-analysis was done, present for each the summary estimate and its precision (e.g. confidence/credible interval) and measures of statistical heterogeneity. If comparing groups, describe the direction of the effect.	Fig 4, 5, Table 1, 2; Table S3, S4
	20c	Present results of all investigations of possible causes of heterogeneity among study results.	Page 7, 9
	20d	Present results of all sensitivity analyses conducted to assess the robustness of the synthesized results.	-
Reporting biases	21	Present assessments of risk of bias due to missing results (arising from reporting biases) for each synthesis assessed.	Table S2; Page 6
Certainty of evidence	22	Present assessments of certainty (or confidence) in the body of evidence for each outcome assessed.	-
**DISCUSSION**			
Discussion	23a	Provide a general interpretation of the results in the context of other evidence.	Page 20-24
	23b	Discuss any limitations of the evidence included in the review.	Page 23
	23c	Discuss any limitations of the review processes used.	Page 23
	23d	Discuss implications of the results for practice, policy, and future research.	Page 24
**OTHER INFORMATION**			
Registration and protocol	24a	Provide registration information for the review, including register name and registration number, or state that the review was not registered.	-
	24b	Indicate where the review protocol can be accessed, or state that a protocol was not prepared.	-
	24c	Describe and explain any amendments to information provided at registration or in the protocol.	Page 25
Support	25	Describe sources of financial or non-financial support for the review, and the role of the funders or sponsors in the review.	Page 25
Competing interests	26	Declare any competing interests of review authors.	Page 25
Availability of	27	Report which of the following are publicly available and where they can be found: template data collection forms; data extracted	Page 25

Section and Topic	Item #	Checklist item	Location where item is reported
data, code and other materials		from included studies; data used for all analyses; analytic code; any other materials used in the review.	

Supplementary Material S1. Figure S1. The relationship between improvement in particular HF-related parameters- by tested medicine agents and drug administration period. Meta-regression lines have been fitted to indicate effect size.

No significant correlation between length of medicine agent administration and alterations in particular parameters in treated animals with HF as compared to the Vehicle (animals with HF reviving placebo) was not denoted. Overall active diastolic function (a); passive stiffness (b); left ventricular end-diastolic dimension (c); pulmonary edema/lung congestion (d); left ventricle hypertrophy (e); fibrosis (f) (N=74 studies). Mean ejection fraction was equal or higher than 50% in heart failure group (Vehicle).

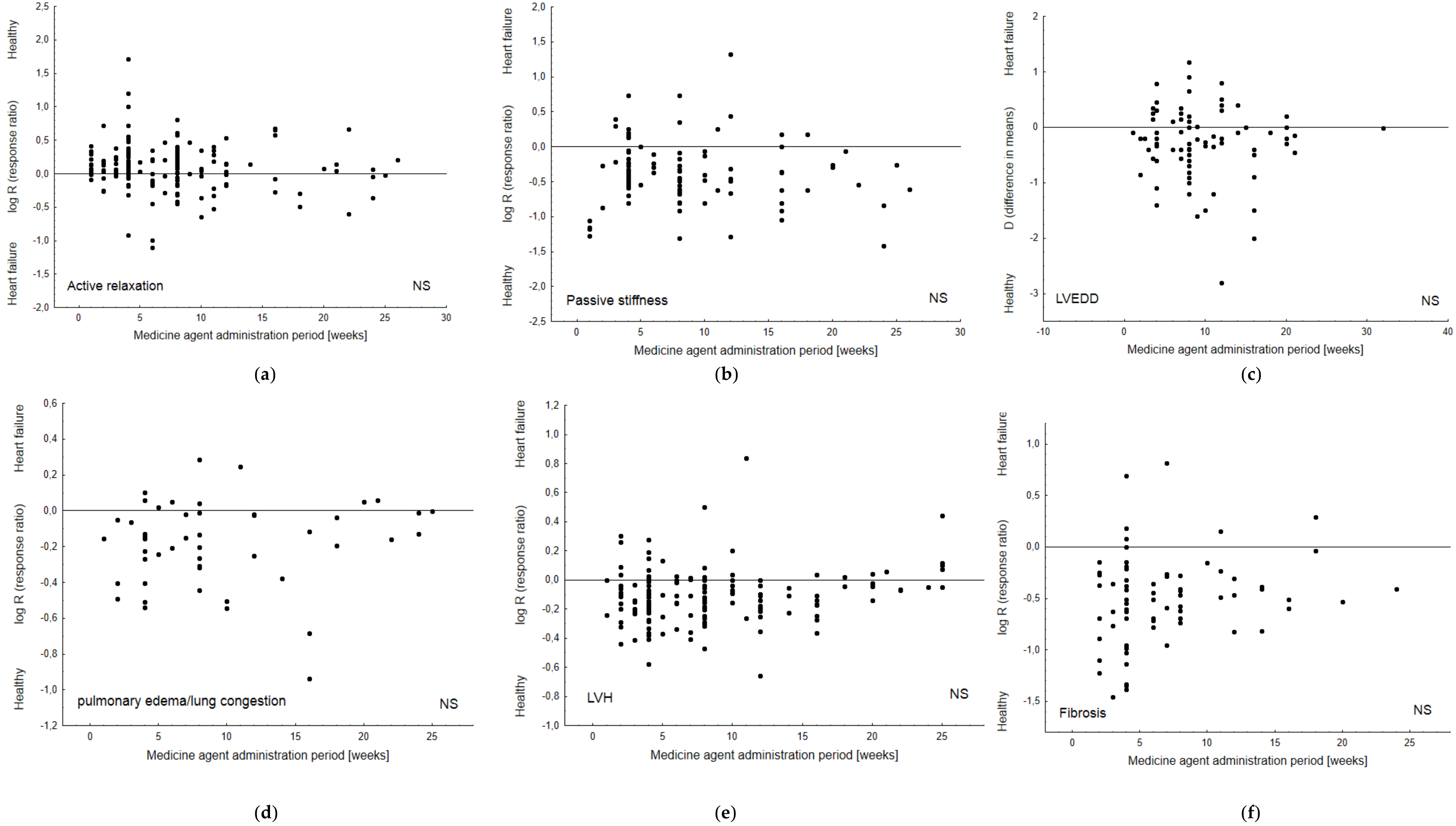

Supplement: Supplementary file 1 [file pharmaceuticals-16-01449-s001.zip › Supplementary Material S1.pdf]
