# Supplementary material for: Searching for Effective Treatments in HFpEF: Implications for Modeling the Disease in Rodents
Source: Pharmaceuticals (Basel). 2023 Oct 12;16(10):1449. doi: 10.3390/ph16101449 (PMC10610318; doi:10.3390/ph16101449)
Supplement: Supplementary file 1 [file pharmaceuticals-16-01449-s001.zip › Supplementary Material S2.pdf]

Supplementary Material S2: Search Criteria.

The databases were searched with no language restrictions using the following search terms in titles and abstracts:

(HEART FAILURE OR SYSTOLIC DYSFUNCTION OR DIASTOLIC DYSFUNCTION)
AND
(ANIMAL OR MOUSE OR MICE OR RAT)
AND
(TREATMENT OR MEDICAL AGENT OR MEDICINE OR DRUG)
AND
(AGEING OR AGING OR FISCHER OR FISHER OR FISCHER344 OR FISCHER 344 OR FVB/N OR F344 OR F344/BN OR FISCHER 344/BROWN NORWAY HYBRID)
OR
(AGNIOTENSIN II INFUSION OR ANG II INFUSION OR ANG II INFUSED OR ANGIOTENSIN II INFUSED)
OR
(ALDOSTERONE INFUSION OR ALD INFUSED OR ALDOSTERONE INFUSED OR ALD INFUSION)
OR
(APOE OR APOLIPOPROTEIN E)
OR
(DAHL-SALT SENSITIVE OR DAHL RAT OR DAHL SALT OR DAHL SALT SENSITIVE)
OR
(DB/DB OR DB DB OR DB MOUSE OR DB MICE OR DIABETIC CARDIOMYOPATHY)
OR
(DOCA SALT OR DEOXYCORTICOSTERONE OR DOCA-SALT OR DOCA-SALT HYPERTENSION OR DOCA HYPERTENSION OR DOCA SALT HYPERTENSION)
OR
(HIGH FAT DIET OR HFD OR WESTERN DIET OF MICE HIGH FAT)
OR
(OB/OB OR OB OB OR OB MOUSE OR OB MICE OR OB OBESITY)
OR
(SHHF OR SPONTANEOUS HYPERTENSION-HEART FAILURE)
OR
(SHR OR SPONTANEOUS HYPERTENSIVE RAT)
OR
(STZ OR STREPTOZOTOCIN OR STZ DIABETES)
OR
(ZUCKER RAT OR ZUCKER OBESE OR ZSF1 OR ZUCKER FATTY OR ZDF OR ZUCKER DIABETIC FATTY OR ZUCKER DIABETIC)
OR
(AORTIC BANDING OR AORTIC CONSTRICTION)
